# Major Psychiatric Disorders, Substance Use Behaviors, and Longevity

**DOI:** 10.1001/jamapsychiatry.2024.1429

**Published:** 2024-06-18

**Authors:** Daniel B. Rosoff, Ali M. Hamandi, Andrew S. Bell, Lucas A. Mavromatis, Lauren M. Park, Jeesun Jung, Josephin Wagner, Falk W. Lohoff

**Affiliations:** 1 Section on Clinical Genomics and Experimental Therapeutics, National Institute on Alcohol Abuse and Alcoholism, National Institutes of Health, Bethesda, Maryland; 2 Radcliffe Department of Medicine, NIH-Oxford-Cambridge Scholars Program, University of Oxford, United Kingdom

## Abstract

**Importance:**

Observational studies suggest that major psychiatric disorders and substance use behaviors reduce longevity, making it difficult to disentangle their relationships with aging-related outcomes.

**Objective:**

To evaluate the associations between the genetic liabilities for major psychiatric disorders, substance use behaviors (smoking and alcohol consumption), and longevity.

**Design, Settings, and Participants:**

This 2-sample mendelian randomization (MR) study assessed associations between psychiatric disorders, substance use behaviors, and longevity using single-variable and multivariable models. Multiomics analyses were performed elucidating transcriptomic underpinnings of the MR associations and identifying potential proteomic therapeutic targets. This study sourced summary-level genome-wide association study (GWAS) data, gene expression, and proteomic data from cohorts of European ancestry. Analyses were performed from May 2022 to November 2023.

**Exposures:**

Genetic susceptibility for major depression (n = 500 199), bipolar disorder (n = 413 466), schizophrenia (n = 127 906), problematic alcohol use (n = 435 563), weekly alcohol consumption (n = 666 978), and lifetime smoking index (n = 462 690).

**Main Outcomes and Measures:**

The main outcome encompassed aspects of health span, lifespan, and exceptional longevity. Additional outcomes were epigenetic age acceleration (EAA) clocks.

**Results:**

Findings from multivariable MR models simultaneously assessing psychiatric disorders and substance use behaviorsm suggest a negative association between smoking and longevity in cohorts of European ancestry (n = 709 709; 431 503 [60.8%] female; β, −0.33; 95% CI, −0.38 to −0.28; *P* = 4.59 × 10^−34^) and with increased EAA (n = 34 449; 18 017 [52.3%] female; eg, PhenoAge: β, 1.76; 95% CI, 0.72 to 2.79; *P* = 8.83 × 10^−4^). Transcriptomic imputation and colocalization identified 249 genes associated with smoking, including 36 novel genes not captured by the original smoking GWAS. Enriched pathways included chromatin remodeling and telomere assembly and maintenance. The transcriptome-wide signature of smoking was inversely associated with longevity, and estimates of individual smoking-associated genes, eg, *XRCC3* and *PRMT6*, aligned with the smoking-longevity MR analyses, suggesting underlying transcriptomic mediators. Cis-instrument MR prioritized brain proteins associated with smoking behavior, including *LY6H* (β, 0.02; 95% CI, 0.01 to 0.03; *P* = 2.37 × 10^−6^) and *RIT2* (β, 0.02; 95% CI, 0.01 to 0.03; *P* = 1.05 × 10^−5^), which had favorable adverse-effect profiles across 367 traits evaluated in phenome-wide MR.

**Conclusions:**

The findings suggest that the genetic liability of smoking, but not of psychiatric disorders, is associated with longevity. Transcriptomic associations offer insights into smoking-related pathways, and identified proteomic targets may inform therapeutic development for smoking cessation strategies.

## Key Points

**Question:**

Are the genetic liabilities of psychiatric disorders, substance use behaviors, or their comorbidity associated with reduced longevity?

**Findings:**

In this cohort study, multivariable mendelian randomization (MR) found that the genetic liability for smoking had a deleterious association with longevity, while corresponding genetic liabilities for major psychiatric disorders had no independent associations when accounting for comorbid substance use in cohorts of European ancestry. Transcriptomic imputation identified 249 smoking-associated genes, including several with associations between smoking and aging, and cis-instrument MR prioritized brain proteins to inform therapeutic development for smoking cessation.

**Meaning:**

The findings in this genetics-based study suggest that reduced healthy aging associated with major psychiatric disorders may be primarily driven by the negative impact of smoking; novel transcriptomic and proteomic targets might offer opportunities for therapeutic drug development for smoking cessation.

## Teaser

This cohort study assesses associations between psychiatric disorders, substance use behaviors, and longevity.

## Introduction

Psychiatric disorders have been associated with morbidity and mortality, including an estimated reduction in life expectancy by 13.5 to 32.2 years.^1^ Unnatural causes contribute greatly,^2^ but chronic physical health conditions also play a substantial role.^3,4^ Tobacco smoking and alcohol consumption have also been associated with decreased lifespans^5,6^ and the comorbidity between psychiatric disorders and substance use behaviors is high.^7^ Given the comorbidity between psychiatric disorders, alcohol consumption, and smoking, elucidating potential factors associated with reduced longevity is important for informing prevention strategies. Though observational findings indicate associations between substance use, psychiatric disorders, and aspects of longevity related to chronic disease and aging, causal inference is limited by residual confounding and reverse causality.^8^ While randomized clinical trials are important for accurately establishing causal inferences by minimizing the limitations of observational research,^9^ conducting randomized clinical trials to assess and disentangle the impact of psychiatric disorders or substance use behaviors on longevity would be complicated by preexisting comorbidities and long durations. Mendelian randomization (MR) uses genetic variants to assess associations between exposures and health outcomes,^10^ and multivariable MR (MVMR) facilitates the simultaneous assessment of correlated exposures by incorporating genetic variants from each risk factor into the same model,^11,12^ making it possible to disentangle the contribution of individual risk factors.^11,12^

We used genome-wide association study (GWAS) data to analyze associations between the genetic liabilities for psychiatric disorders, alcohol consumption, smoking, and longevity outcomes related to chronic, age-related diseases and epigenetic age acceleration (EAA) as measured by DNA methylation.^13,14,15,16^ We implemented 2-sample MR^10^ (eFigure 1 in Supplement 1) to assess genetics-based associations of psychiatric disorders and substance use behaviors with longevity. We next found gene-level associations underlying smoking behavior and connected these genes with longevity and EAA using transcriptomic imputation.^17^ The targets of most approved drugs are proteins,^18^ and identifying smoking-related proteins could inform drug development for smoking cessation. Therefore, we performed cis-instrument MR of brain proteins,^19^ followed by phenome-wide MR studies evaluating adverse-effect profiles to prioritize targets for future smoking cessation therapeutic development.

## Methods

### Data Sources

**Figure 1.**
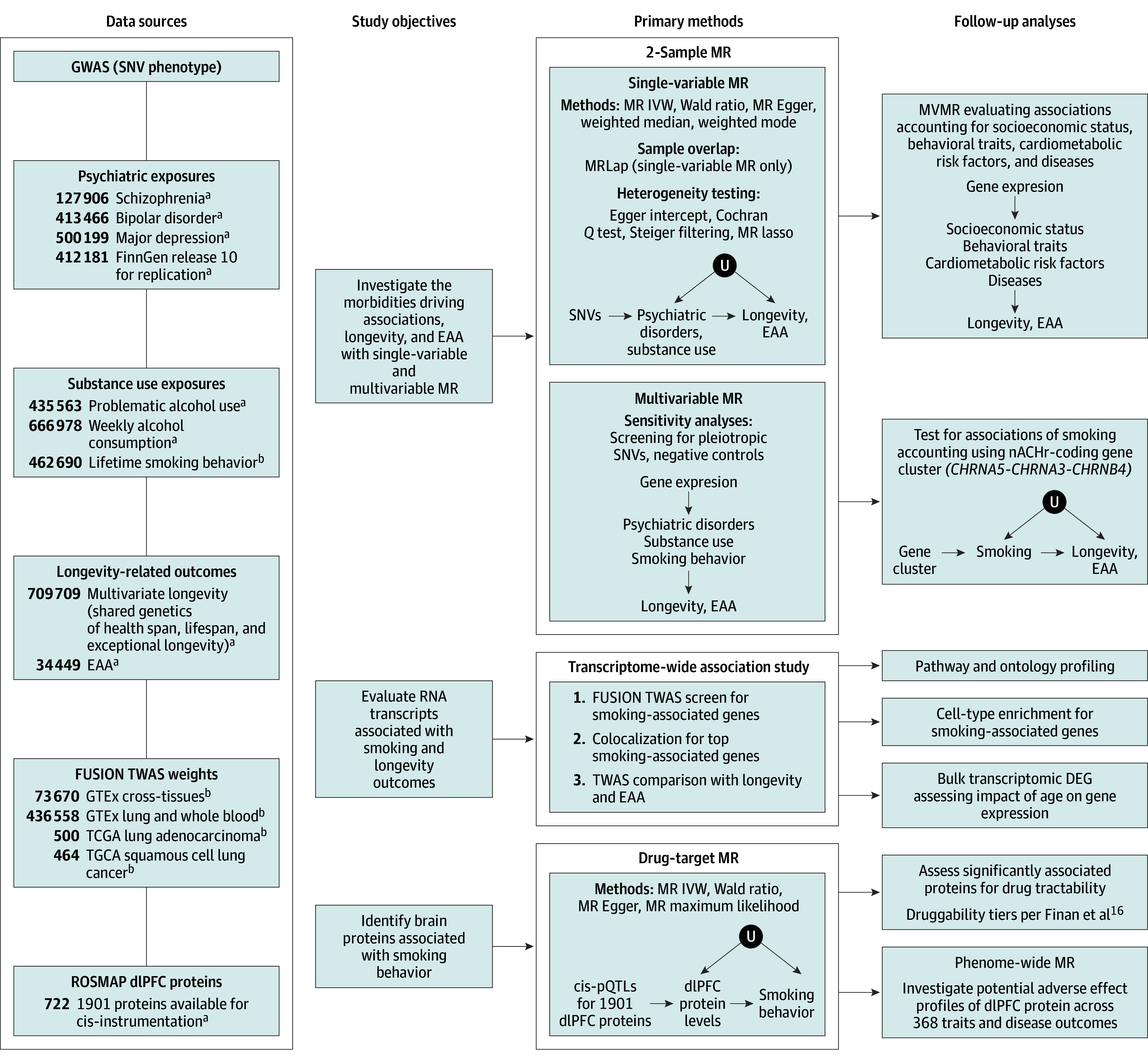
Study Overview Presented is the study flow diagram outlining the data sources (categorized by their primary roles in the analyses), methods, and follow-up analyses. dlFPC indicates dorsolateral prefrontal cortex; EAA, epigenetic age acceleration; GTEx, genotype-tissue expression; GWAS, genome-wide association study; IVW, inverse variance weighted; MR, mendelian randomization; MVMR, multivariable MR; nACHr, neuronal nicotinic acetylcholine receptor; pQTL, protein quantitative trait locus; ROSMAP, Religious Orders Study and Rush Memory and Aging Project; SNV, single-nucleotide variant; TCGA, The Cancer Genome Atlas; TWAS, transcriptome-wide association study. ^a^Meta-analysis GWAS or QTL study. ^b^Single-cohort GWAS or QTL study.

This study examined the genetic basis of psychiatric disorders, substance use behaviors, and longevity outcomes using data from genome-wide association studies (GWASs) in European populations (Figure 1). Data included schizophrenia,^20,21^ bipolar disorder,^21,22^ major depression,^21,23^ smoking behaviors,^24^ and alcohol use,^21,25,26^ along with a multivariate GWAS on health span, lifespan, and longevity^27^ and epigenetic clocks (first and second generation).^13,14^ Additional sources included postmortem brain protein data,^19^ blood gene expression,^28^ and GWASs of various biomarkers and diseases (eTable 1 in Supplement 2). Datasets have existing ethical permissions from their respective institutional review boards and participant informed consent with rigorous quality control. MR analyses are reported according to the Strengthening the Reporting of Observational Studies in Epidemiology using Mendelian Randomization (STROBE-MR) reporting guideline (eAppendix in Supplement 1).

### MR Instruments

Primary single-variable MR (SVMR) instruments included genome-wide significant single-nucleotide variants (SNVs) (*P* < 5 × 10^−8^; linkage disequilibrium *R*^2^ < 0.001). Prior to constructing MVMR, we calculated cross-trait genetic correlations evaluating whether these exposures share common genetic factors using LDSC,^29^ which would suggest potential pleiotropic associations, motivating the MVMR to better account for pleiotropy and distinguish between direct and indirect associations of the exposures. Psychiatric disorders, smoking, and alcohol consumption SNVs were concatenated to generate MVMR instruments. We constructed MVMR instruments evaluating all psychiatric disorders and substance use behaviors simultaneously and separate MVMR instruments assessing the independent associations of psychiatric disorders and alcohol consumption while accounting for smoking behavior (eMethods in Supplement 1; eTables 2-8 in Supplement 2). We constructed another lifetime smoking instrument using *CHRNA5-CHRNA3-CHRNB4* gene cluster, encoding nicotinic acetylcholine receptors^30^ (eMethods and eFigure 2 in Supplement 1). Cortical protein quantitative trait locus cis*-*instruments included independent SNVs (*P* < 5 × 10^−8^; ±100 kilobases of the gene locus) (eMethods in Supplement 1; eTable 9 in Supplement 2).

### MR Assumptions

MR uses genetic variants from GWAS as instruments to analyze associations between exposures and outcomes, and relies on 3 core assumptions: relevance, independence, and exclusion restriction (eMethods in Supplement 1).^31^ Relevance requires the genetic instruments are linked to the exposure. Independence requires that the instruments are not connected with any confounders that might impact the exposure-outcome relationship. Exclusion restriction requires the instruments impact the outcome only through the exposure, not through other paths.^31^ When satisfied, MR can test for a causal association without bias from unobserved confounding.^31^ An additional assumption of homogeneity must be satisfied for accurate average causal effect (ACE) estimations in MR,^31^ which requires the association of the exposure on the outcome is consistent, and not varied by unobserved factors; otherwise, the ACE estimates might not be valid for the general population.^32^ To assess the plausibility of the assumptions, including homogeneity, we fine-tuned our instruments, removing SNVs incrementally, and assessing the consistency of our results across these changes, providing evidence against violations of the assumptions.^33^

### Transcriptomic imputation

We used the Functional Summary-Based Imputation (FUSION) method^17^ to identify tissue-specific transcript-level associations with lifetime smoking. Transcriptomic imputation integrated the lifetime smoking GWAS with gene expression–level FUSION weights from cross-tissue data, whole blood Genotype-Tissue Expression project,^34^ and The Cancer Genome Atlas^35^ lung cancer biopsies.^35^ We used the coloc package version 5.1.0 in R version 4.0.2 (R Foundation).^36^ Genes with colocalization posterior probabilities (PP.H4) greater than 0.8 were taken forward for comparison with longevity and EAA, performing bioannotation, including ontology and pathway analysis, cell-type enrichment, and age-related expression change (eMethods in Supplement 1). Comparing identified gene-tissue features with the lifetime smoking GWAS lead SNVs, we indexed prioritized features as novel signals if located 1 or more megabase (Mb) from lead smoking SNVs.

### Reporting and Interpreting Results

MR estimates correspond to the outcome change per unit increase in exposure (eg, SD of the lifetime smoking index) of the lifetime smoking index; we emphasize their interpretation as reflecting the genetic liabilities for the psychiatric disorders or substance use behaviors, given their time-varying and multifactorial nature.^37,38^ Genetic liability refers to the genetic predisposition toward or susceptibility to a particular trait or disease, based on the premise that SNVs influence the risk or likelihood of developing certain traits or diseases.^39^ Further, the MR findings should be interpreted in the context of the MR assumptions (eMethods in Supplement 1). We assessed SVMR results using a Bonferroni threshold (0.0083 [0.05/6 exposures]) and took forward MVMR associations surpassing this threshold for follow-up analyses. Transcriptomic imputation results are reported as *z* scores and were assessed using a Bonferroni threshold (8.31 × 10^−7^ [0.05/60 114 tests]).

### Statistical Analysis

We used inverse-variance weighted MR as the main method, complemented by MR Egger, weighted median, penalized weighted median, and weighted mode analyses. Consistency of results across methods facilitates assessment of the validity of the core MR assumptions and strengthens inference.^40^ Their multivariable extensions^12,41^ were used for MVMR analyses estimating the direct association of the genetic liabilities for each psychiatric disorder and substance use behavior with longevity and EAA. Instrument heterogeneity was assessed using the MR Egger intercept,^42,43^ the Cochran *Q* heterogeneity test, and multivariable extensions thereof.^44^ MR-Lasso^43^ and the Steiger directionality test^45^ were used to identify and remove outlier instruments, and the assumed direction between selected exposures and outcomes, respectively. For exposure-outcome pairs with sample overlap (eMethods in Supplement 1; eTable 10 in Supplement 2), we used MRLap, which accounts for sample overlap, winner’s curse, and weak instrument bias.^46^ See the eMethods in Supplement 1 for an extended description of these MR statistical analyses.

We performed additional sensitivity analyses further evaluating the validity of the MR assumptions (eMethods in Supplement 1), including negative control analyses to assess selection bias, additional screening of exposure SNVs for associations with potential confounders; extended MVMR models incorporating traits related to socioeconomic status, behavioral traits (eg, physical activity and sleep), and cardiometabolic biomarkers; replication with independent psychiatric disorder exposure data to evaluate the robustness of the associations and assess biases related to the presence of retrospective case-control cohorts in several of the GWAS meta-analyses; and iterative removal of heterogeneous SNVs to assess the homogeneity assumption. Cis-instrument MR used either the inverse-variance weighted MR or Wald ratio (1-SNV instruments), and MR Egger assessed pleiotropy for instruments with 3 or more SNVs.^47^ We performed colocalization^36^ for the proteins surpassing Bonferroni correction and replication with whole blood gene expression (eMethods in Supplement 1).

Analyses were performed using TwoSampleMR version 0.5.7, MendelianRandomization version 0.9.0, LD Score Regression version 1.0.1, Phenoscanner version 2, MRLap version 0.0.2, WebCSEA, PrismEXP version 0.2.7, and EnrichR version 3.2. The analyses using TwoSampleMR, MendelianRandomziation, and MRLap methods used *P* value thresholds outlined in the preceding paragraphs. FUSION transcriptome-wide association used a Bonferroni-corrected *P* value threshold of 8.3 × 10^−7^ (0.05/60 114 total tests across the panels) to assess genes associated with lifetime smoking behavior. Because they were follow-up annotation of the transcriptome-wide association findings, the results for the PrismEXP, EnrichR, and WebCSEA used *P* value thresholds of .05.

## Results

### Cross-Trait Genetic Correlations of Psychiatric Disorders and Substance Use Behaviors

Smoking and longevity associations were assessed in a cohort of 709 709 (431 503 [60.8%] female and 278 206 [39.2%] male). Significant genetic correlations were found between smoking and several exposures, including bipolar disorder (*r*, 0.17; SE, 0.02; *P* = 1.89 × 10^−14^), drinks per week (*r*, 0.34; SE, 0.02; *P* = 6.59 × 10^−63^), major depression (*r*, 0.32; SE, 0.03, *P* = 9.64 × 10^−31^), problematic alcohol use (*r*, 0.52; SE, 0.03; *P* = 3.94 × 10^−80^), and schizophrenia (*r*, 0.21; SE, 0.02; *P* = 5.61 × 10^−21^). Among the traits, the strongest correlation was found between bipolar disorder and schizophrenia (*r*, 0.70; SE, 0.03; *P* = 8.87 × 10^−147^),  while the weakest correlation was between drinks per week and major depression (*r*, 0.07; SE, 0.02; *P* = 2.77 × 10^−3^), underscoring the shared genetic architecture across these traits and motivating MVMR (eFigure 3 and eResults in Supplement 1; eTable 11 in Supplement 2).

### Associations of Psychiatric Disorders and Substance Use Behaviors With Longevity

**Figure 2.**
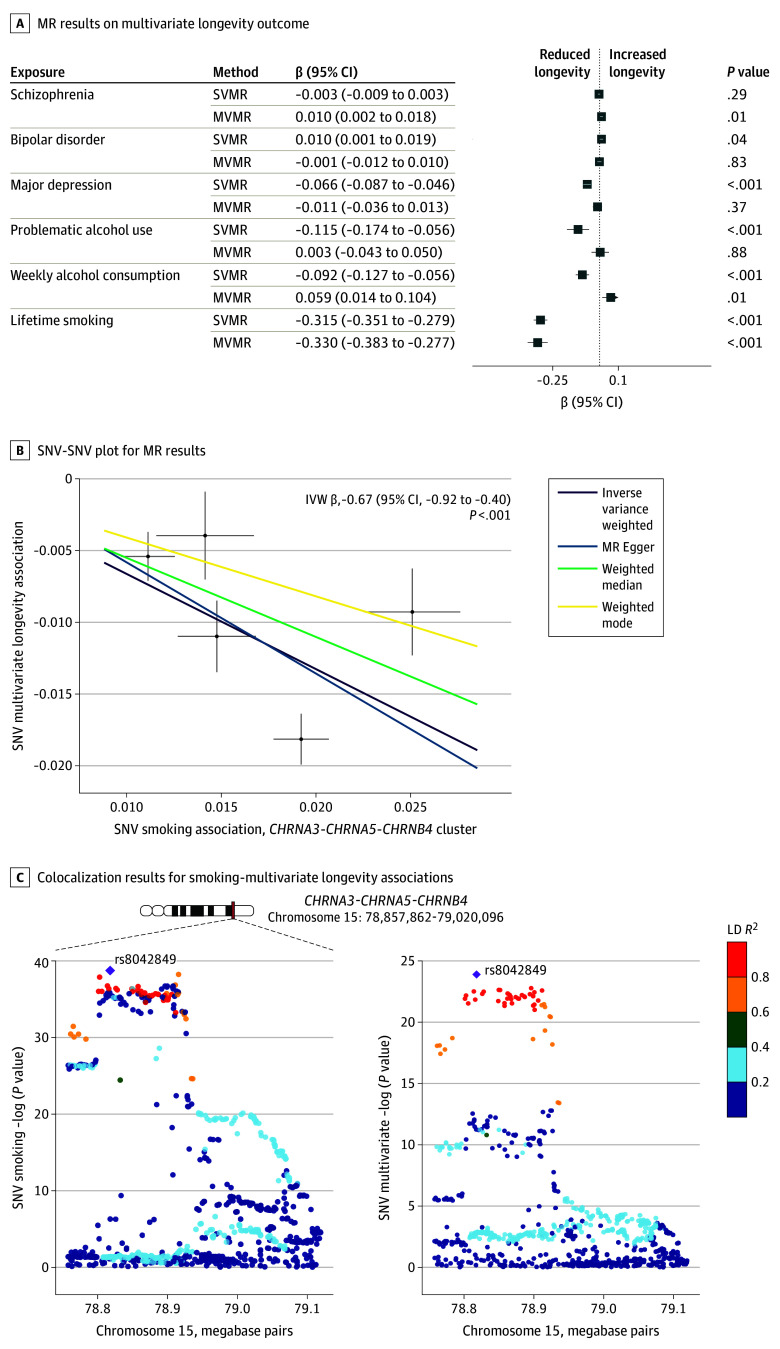
Mendelian Randomization (MR) Results of Association of Neuropsychiatric Disorders and Substance Use Behaviors With Human Longevity A, Estimated associations reported are MR estimates with 95% CIs. Multivariable MR (MVMR) results reported are MR estimates from the final 2 MVMR models incorporating all psychiatric disorders, smoking behavior, and 1 of the alcohol consumption exposures simultaneously. B, Points plotted are the associations statistics for the 5 variants comprising the *CHRNA5-CHRNA3-CHRNB4* gene cluster smoking instrument. Regression lines shown correspond to the main inverse variance–weighted (IVW) and complementary MR methods. C, Single-nucleotide variants (SNVs) are colored by their linkage disequilibrium (LD) *R*^2^, and the labeled SNV rs8042849 is the candidate causal SNV shared between smoking behavior and the multivariate longevity outcome. SVMR indicates single-variable MR.

SVMR estimates showed negative associations between longevity and the genetic liability for major depression, lifetime smoking (longevity β, −0.33; 95% CI, −0.38 to −0.28; *P* = 4.59 × 10^−34^), and drinks per week, but not bipolar disorder or schizophrenia (Figure 2; eTable 12 in Supplement 2). Inverse-variance weighted estimates were consistent with estimates from MRLap^46^ (eTable 13 in Supplement 2), which was used to assess bias due to sample overlap (eMethods in Supplement 1), and replication analyses using the FinnGen cohort^21^ diagnoses of schizophrenia, bipolar disorder, major depression, and alcohol use disorder (eTable 14 in Supplement 2). SVMR estimates were consistent with instruments that removed SNVs with associations with other GWAS data (eTable 15 in Supplement 2), supporting the plausibility of the MR exclusion restriction assumption. Estimates aligned with the univariate lifespan outcome (eTable 16 in Supplement 2) used to assess bias related to the multivariate longevity outcome construction (eMethods in Supplement 1). SVMR estimates were directionally consistent across the primary and revised instrument sets with heterogenous SNVs removed (eTable 17 in Supplement 2), supporting the plausibility of the homogeneity assumption.^33^

Instrumenting smoking with the *CHRNA5-CHRNA3-CHRNB4* gene cluster SNVs corroborated smoking-longevity associations (Figure 2B; eTable 18 in Supplement 2), including colocalization evidence (PP.H4 = 99.8%) (eFigure 4 in Supplement 1; eTable 19 in Supplement 2). MVMR assessing the association of smoking behavior with each of the psychiatric disorders and alcohol use behaviors in turn found that, while smoking continued to have an adverse association with longevity, the adverse impact of major depression, problematic alcohol use, and drinks per week on longevity attenuated with MVMR, suggesting that the genetic liabilities of these exposures had no direct associations with longevity (eTable 20 in Supplement 2). The smoking-longevity association was robust in the MVMR models simultaneously assessing the impact of the genetic liabilities for major psychiatric disorders, lifetime smoking, and drinking behavior on longevity, showing an independent adverse impact of smoking on longevity (Figure 2A; eTables 21-22 in Supplement 2). SVMR and MVMR estimates were consistent across the MR methods, further supporting genetics-based inference; associations were also robust across a range of MVMR models adjusting for other risk factors and cardiometabolic phenotypes (eTables 23-24 in Supplement 2). Negative control analyses assessing the impact of the psychiatric disorders and substance use behaviors on self-reported tanning ability were null (eTable 25 in Supplement 2), suggesting the results were not biased by hypothesized predictors of selection.^48,49^

### Associations of Psychiatric Disorders and Substance Use Behaviors With EAA

**Figure 3.**
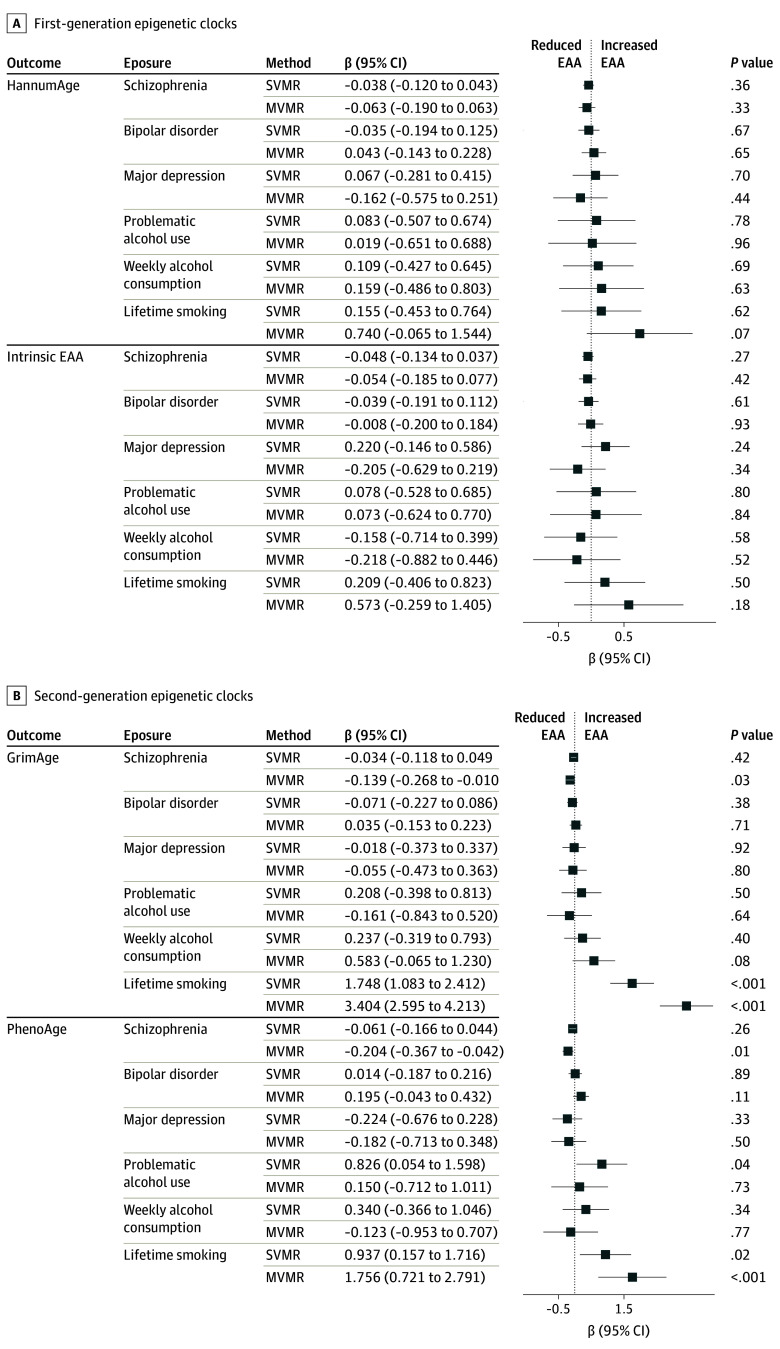
Mendelian Randomization (MR) Results of the Association of Neuropsychiatric Disorders and Substance Use Behaviors With Epigenetic Age Acceleration (EAA) Associations reported are MR estimates with 95% CIs. Multivariable MR (MVMR) results reported are MR estimates from the final 2 MVMR models incorporating all psychiatric disorders, smoking behavior, and 1 of the alcohol consumption exposures simultaneously. SVMR indicates single-variable MR.

Associations of psychiatric disorders and substance use behaviors with EAA were assessed in a cohort of 34 449 (18 017 [52.3%] female and 16 432 [47.7%] male). Associations were found only with second-generation clocks, including an association of smoking and PhenoAge at *P* < .05 (β, 1.76; 95% CI, 0.72-2.79; *P* = 8.83 × 10^−4^)] (Figure 3; eTable 12 in Supplement 2), consistent with the association between smoking and decreased longevity. Problematic alcohol use was also associated with PhenoAge at *P* < .05 (β, 0.83; 95% CI, 0.05-1.60; *P* = .04). The EAA associations with the genetic liability of smoking accounting for the 3 psychiatric disorders and alcohol behaviors, individually and jointly, surpassed correction for multiple comparisons (Figure 3). Estimates were consistent after removing SNVs associated with other GWASs, and robust in the replication FinnGen data, and extended MVMR models adjusting for socioeconomic, lifestyle factors, and cardiometabolic outcomes (eTables 14, 15, 17, and 20-24 in Supplement 2). Further, the associations between smoking and GrimAge in analyses with the *CHRNA5-CHRNA3-CHRNB4* gene cluster remained robust, while the association with PhenoAge was directionally consistent, but attenuated to the null (eTable 18 in Supplement 2). SVMR associations were not observed between either the psychiatric disorders or the alcohol behaviors and EAA.

### Transcriptomic Imputation and Novel Genes for Lifetime Smoking

**Figure 4.**
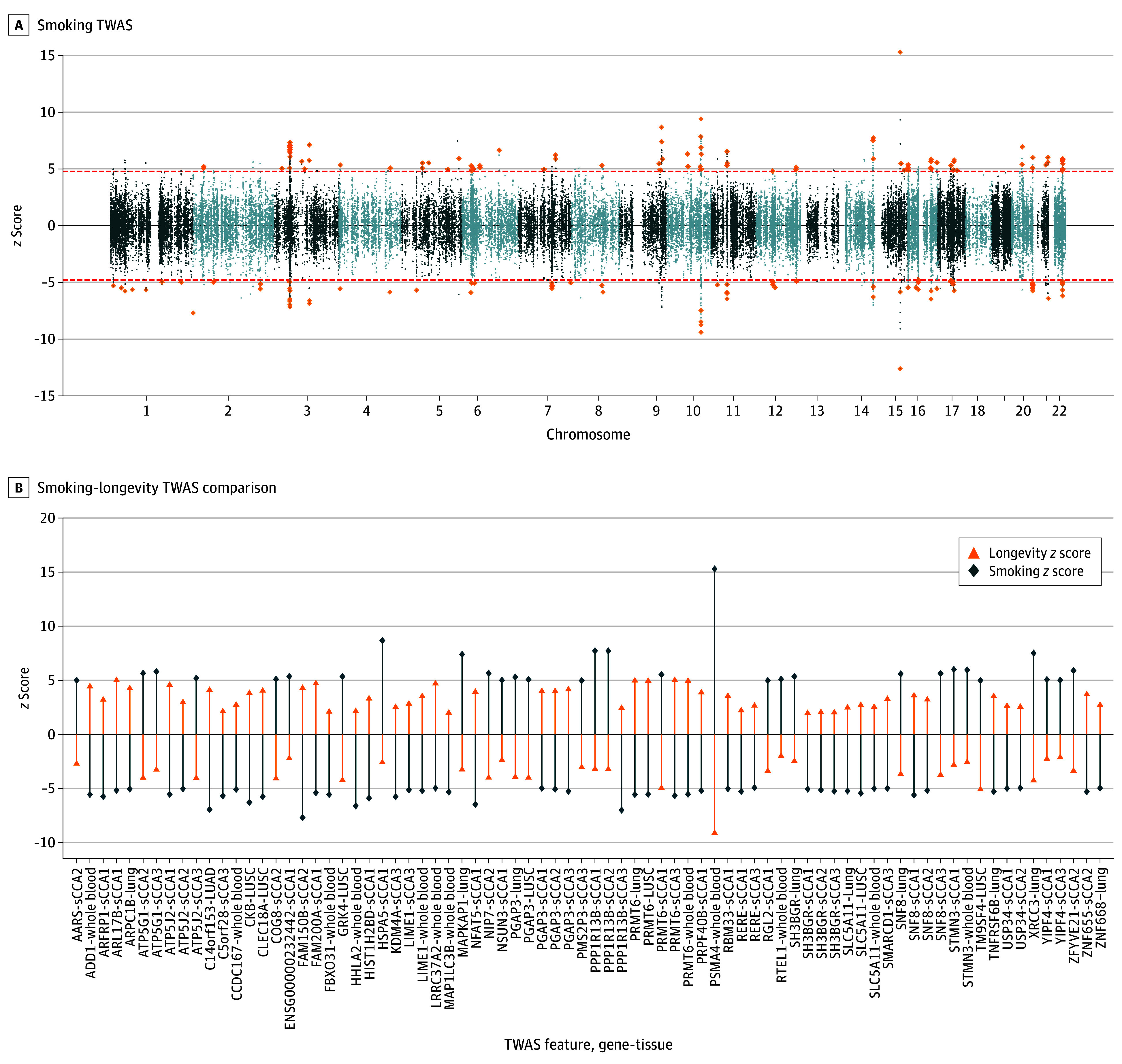
Transcriptomic Imputation Results Finding Genes Associated With Smoking Behavior and Comparison With Human Longevity Panel A presents the FUSION transcriptome-wide association study (TWAS) identifying genes associated with lifetime smoking behavior. The y-axis shows the TWAS *z* scores for smoking behavior for each gene-tissue feature analyzed and the x-axis shows the genomic position of the gene. Highlighted genes are the high-confidence smoking-associated genes that surpassed correction for multiple comparisons (*P* = 8.31 × 10^−7^) and further demonstrated evidence of colocalization (posterior probability >0.8) with smoking behavior. Panel B presents the comparison of the high-confidence smoking-associated TWAS features (gene-tissue combinations) with the multivariate longevity outcome that were directionally consistent with the mendelian randomization analyses (ie, had an inverse association with smoking and longevity) and further had TWAS *P* < .05 for longevity (eMethods in Supplement 1). The y-axis shows the TWAS *z* scores for smoking behavior and longevity for each gene-tissue feature analyzed.

Of 249 unique genes identified (Figure 4A; eFigures 5-9 in Supplement 1; eTables 26-30 in Supplement 2), 36 were novel (eTable 31 in Supplement 2). Colocalization was found between 150 unique genes and lifetime smoking behavior, suggesting these genes act as transcriptome-level mediators (eTable 32 in Supplement 2). High-confidence genes were implicated in biological processes, such as DNA repair, chromatin remodeling, and telomere maintenance (eFigure 10 in Supplement 1; eTable 33 in Supplement 2). Further investigation across 1355 cell types revealed enrichment in white blood cells and cardiac muscle cells (eFigures 11-12 in Supplement 1; eTable 34 in Supplement 2**)**, and look-up of smoking-associated genes in the Open Genes Aging database^50^ identified *SHC1*, *XRCC6*, and *DGKZ* as having moderate to high involvement with aging processes (eTable 35 in Supplement 2).

We next evaluated the associations of colocalized genes shared between smoking and longevity, revealing 31 genes with consistent associations surpassing correction for multiple comparisons with smoking and longevity outcomes (eTable 36 in Supplement 2); for example, increased *PRMT6* expression was associated with increased smoking behavior and reduced longevity in both healthy whole blood, healthy lung, and cancerous lung tissue. Forty-six additional genes had directionally consistent associations with longevity or EAA at *P* < .05 (eg, *PSMA4 z* score on longevity, −9.11; *P* = 7.90 × 10^−20^; *ADD1 z* score on longevity, 4.43; *P* = 9.43 × 10^−6^; *TOP2B z* score on GrimAge, 2.63; *P* = .009) (Figure 4B; eFigure 13 in Supplement 1**)**, suggesting transcriptomic links between smoking and longevity. Differential expression analysis using age-dependent RNA sequencing Genotype-Tissue Expression data found 25 genes showing evidence of aging-related expression changes across several tissues (eTable 37 in Supplement 2); for example, *PRMT6* and *XRCC3* expression was lower in older individuals in 3 tissues (muscle, blood, and stomach), aligning with transcriptomic imputation direction of associations with longevity. Transcriptome-wide correlations between smoking and the aging-related traits showed significant, gene-level correlations directionally consistent with MR analyses in each of the cancerous and healthy tissues (eFigures 14-16 and eResults in Supplement 1).

### Druggable Genome Cis-Instrument MR and Smoking Cessation Targets

**Figure 5.**
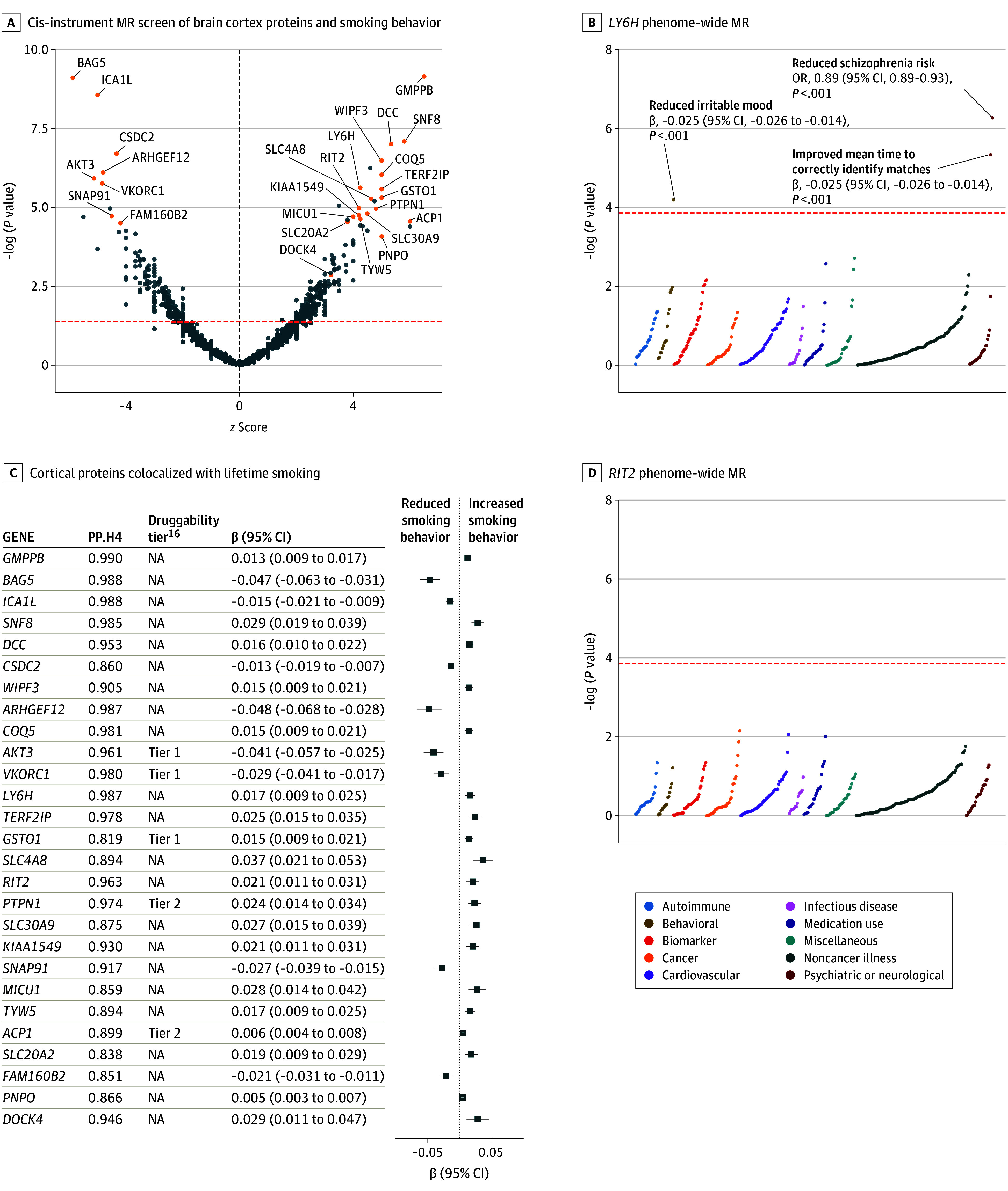
Results of Cis-Instrument Mendelian Randomization (MR) Screen for Brain Proteins Associated With Smoking Behavior A, Labeled proteins are those that surpassed false discovery rate correction for multiple comparisons and also demonstrated evidence of colocalization with smoking behavior (PP.H4 > 0.8; eMethods in Supplement 1). B, There were 27 cortical proteins that colocalized with lifetime smoking, including the cis-MR estimates, colocalization PP.H4 value, and druggability tiers (eMethods in the Supplement). C and D, *LY6H* and *FIT2* were 2 of the proteins that surpassed false discovery rate correction for multiple comparisons and also demonstrated evidence of colocalization with smoking behavior, plotted here against 367 diseases and biomarkers (eMethods in Supplement 1; eTable 1 in Supplement 2). The red dotted lines indicated the adjusted *P* value threshold of 1.36 × 10^−4^ (0.05/367 outcomes tested), and labeled outcomes in (C) surpassed correction for multiple comparisons. The direction of the MR estimates for each of these labeled outcomes (schizophrenia, irritable mood, and mean time to correctly identify matches) suggested a beneficial association directionally consistent with the direction that would be therapeutically beneficial to reduce smoking behavior (eg, for *LY6H*, this corresponds to inhibition).

The cis-instrument MR screen identified 135 cortical proteins associated with smoking behavior surpassing false discovery rate correction (Figure 5A; eTable 38 in Supplement 2). Twenty-one of the proteins are considered druggable^18^ (eTable 39 in Supplement 2). Twenty-seven of the 135 proteins also colocalized with smoking behavior, including tier 1 druggable genes (*AKT3*, *GSTO1*, and *VKORC1*) (Figure 5C; eTable 40 in Supplement 2). MR estimates suggested that for 8 of the colocalized proteins, the correct therapeutic direction is increased protein levels, while the other 19 would require inhibition (eTable 41 in Supplement 2). As the postmortem cortical quantitative trait locus data may be subject to confounders related to selection biases and tissue preservation,^51^ we assessed evidence of replication using whole blood expression quantitative trait locus data collected from living donors. Of the 18 proteins available for assessment in whole blood (eTable 42 in Supplement 2), we replicated 6 (MICU1, CSDC2, COQ5, AKT3, SNF8, and SLC20A2) at the same stringent screen *P* threshold and additional 3 at *P* < .05 (BAG5, GSTO1, and FAM160B3) (eTable 43 in Supplement 2).

Phenome-wide MR results suggested favorable adverse-effect profiles (ie, any MR estimates surpassing correction for multiple comparisons aligned with the therapeutic direction for smoking behavior) for most of the 27 proteins (eTable 44 in Supplement 2). For example, *LY6H *(lifetime smoking β, 0.02; 95% CI, 0.01-0.03; *P* = 2.37 × 10^−6^), *RIT2* (lifetime smoking β, 0.02; 95% CI, 0.01-0.03, *P* = 1.05 × 10^−5^) (Figure 5B and D), *AKT5*, *SNF8*, and *DOCK4*, while targets, like *VKORC1* and *ACP1*, had several phenome-wide MR associations suggesting possible adverse effects, such as increased risk of Alzheimer disease (*VKORC1*) and malignant melanoma (*ACP1).*

## Discussion

This cohort study used genetic approaches to disentangle the associations of the genetic liabilities for psychiatric disorders and substance use behaviors with genetically predisposed longevity. Multiomics analyses elucidated underlying transcriptomic mediators of these associations and prioritized proteomic targets. SVMR analyses found the genetic liabilities for smoking, alcohol consumption, and major depression were linked with reduced longevity related to chronic and age-related diseases; however, our MVMR analyses found that the genetic liability for smoking was associated with a reduction in longevity related to chronic and age-related diseases, suggesting that smoking is an important mediator for reduced healthy aging and increased chronic illness observed in psychiatric populations. Findings were consistent across complementary MR methods and robust to sensitivity analyses, including filtering variants for alternate potential pathways, alternate instruments using variants in the *CHRNA5-CHRNA3-CHRNB4* gene cluster, and additional MVMR models accounting for socioeconomic factors, biomarkers, and cardiometabolic diseases (eDiscussion in Supplement 1; eFigure 17 in Supplement 2). However, it is important to underscore that our findings do not negate the potential impact of the genetic liabilities of psychiatric disorders or alcohol consumption on healthy aging and longevity. Instead, they underscore the importance of considering the genetic liability for smoking. For example, the lack of adverse genetic-based associations between schizophrenia, bipolar disorder, and longevity raises intriguing questions about the complex genetic architecture of neuropsychiatric disorders; for example, certain genetic aspects may provide adaptive mechanisms to enhance resilience and healthy behaviors while others, like smoking, may reduce health (eDiscussion in Supplement 1). More broadly, MR assumes variations in the results linked to the genetically estimated amounts of exposure would be similar to those observed if there were direct interventions on the exposure.^52^ As the psychiatric disorders and substance use behaviors and corresponding genetic liabilities are multifactorial, their MR instruments may reflect different potential interventions having different associations with longevity and biological aging, which would lead to bias from deviations from the consistency assumption.^52,53^ While MR estimates were derived after removal of outlier SNVs and screening for pleiotropic pathways that may be more likely to reflect instruments of other interventions and pathways, we nevertheless emphasize that causal inference requires triangulating lines of evidence,^54,55^ including studies capable of defining specific interventions,^53^ improving our understanding of how mental health impacts aging.

Transcriptomic imputation prioritized 150 unique genes, including 27 high-confidence novel genes, while downstream annotation revealed connections with the longevity-related outcomes and underlying biological aging processes; for example, the pathway annotation implicated DNA repair, chromatin remodeling, and telomere assembly and maintenance. DNA repair and maintenance of chromatin structure are crucial for preserving genomic integrity, while telomere maintenance is intricately linked to cellular stress and aging.^56^ Given the strong potential of smoking to cause DNA damage in exposed tissues,^57^ our results suggest possible targets and pathways to ameliorate the adverse aging-related effects of smoking. Transcriptome-wide association study results also indicated that smoking and longevity phenotypes had transcriptome-wide correlations, aligning with MR findings and suggesting a complex interplay between genetic factors, smoking behavior, transcriptomic mediators, and aging. High-confidence genes, such as *XRCC3*, *SHC1*, *PRMT6*, *TOP2B*, and *ARL17B*, particularly those involved in DNA repair and telomere maintenance, are insightful for understanding how smoking affects aging processes. For instance, the role of *XRCC3* in genomic integrity maintenance^58^ and the influence of its polymorphisms on smoking-related cancer risk^59^ highlight its importance. SHC1, an adaptor protein linked to survival and stress response pathways and known for its aging-related properties,^60^ could be pivotal in addressing smoking-induced cellular dysfunction and age-related decline (eDiscussion in Supplement 1).

Cis-instrument MR identified 135 cortical proteins linked to lifetime smoking, with colocalization prioritizing 27 for potential smoking cessation strategies. Phenome-wide MR evaluated their adverse-effect profiles, highlighting LY6H and RIT2 as promising candidates. The regulatory effect of LY6H on neuronal nicotinic acetylcholine receptors suggests its potential in cholinergic signaling pathways related to smoking.^61^ Additionally, phenome-wide MR indicated potential neuropsychiatric benefits of LY6H without adverse effects. For RIT2, preclinical studies suggest an important role in dopaminergic signaling pathways (eg, requisite for dopamine transporter internalization),^62^ offering a promising approach aimed at addressing the dysregulation of dopaminergic transmission underlying nicotine dependence,^63^ in turn offering a novel approach to addressing nicotine dependence.^63^

### Limitations

There are study limitations. First, while results from extensive sensitivity analyses suggested that the MR assumptions are plausible for these analyses, we underscore the importance of interpreting the MR results through the lens of all MR assumptions (ie, core assumptions and homogeneity, consistency, and same population assumptions^38^) (eDiscussion in Supplement 1). For example, while negative control analyses and MVMR with predictors of selection suggested that the results were not impacted by selection bias, there may still be differences in the construction of the cohorts that may influence the results. The cis-instrument MR using cortical protein data from postmortem data may be susceptible to selection bias; however, we were able to analyze the top targets in additional whole blood data from living donors, increasing confidence in the findings. Second, we emphasize consideration of the inherent complex characteristics of the study exposures and outcomes, including the time-varying nature of the exposures, such as changes in lifetime smoking patterns, which may not be fully captured by the corresponding genetic instruments reflecting lifelong liabilities for these traits.^37^ Third, analyses were performed using data derived from cohorts of European ancestry—caution is necessary before generalizing the findings to other populations (eDiscussion in Supplement 1). Fourth, while differences in the smoking behaviors of men and women have decreased over time,^64^ differences still exist, suggesting there may be a sex effect on smoking-longevity associations; however, there are currently no sex-stratified longevity data available to assess this potential effect using MR. Fifth, while the longevity outcome captured the genetics of health span and lifespan, important to facilitate assessment of impaired aging related to chronic illness, aging is a complex, multifaceted process,^65^ and the outcome does not capture all age-related processes (eDiscussion in Supplement 1). Additionally, while the MVMR models may inform the associations outlined in Figure 1 and eFigure 17 in Supplement 1, they cannot assess potential biases due to sources of pleiotropy via all pathways influencing longevity.^11,12^ Sixth, sample overlap across sourced GWASs may bias results; however, we incorporated replication and recently developed MR methods adjusting estimates for sample overlap,^46^ and observed that, confounding due to correlation notwithstanding, 2-sample MR methods may be safely used with single-sample MR using GWASs generated from large biobanks.^66^ Seventh, regarding the transcriptomic imputation findings, given the cross-sectional nature of our primary data sources, we acknowledge the challenge of the direction of effect between smoking behavior and gene expression changes, underscoring the need for future replication of our findings using longitudinal transcriptomic data, which would offer a more dynamic perspective on these associations.

## Conclusions

We used genomic methods facilitating simultaneous investigation of the major psychiatric disorders and substance use behaviors and found the genetic liability for smoking, but not drinking, major depression, bipolar disorder, or schizophrenia, had an adverse independent association with longevity and EAA. Transcriptomic analyses prioritized 249 smoking-related genes, which were enriched in important aging-related processes, providing insight into the pathways between smoking and healthy aging and longevity. Prioritized targets may inform future investigation into therapeutic development to add smoking cessation efforts.

## Notes

Supplement 1. eMethods eResults eDiscussion eAppendix. STROBE-MR Reporting Guidelines eFigure 1. Mendelian Randomization Model and Assumptions eFigure 2. Mendelian Randomization (MR) Model and Assumptions applied to CHRNA5-CHRNA3-CHRNB4 gene cluster eFigure 3. Cross-trait genetic correlations between the psychiatric disorders and substance use behaviors used as Mendelian randomization exposures eFigure 4. SNV-SNV plot for Mendelian Randomization (MR) results assessing the relationship of smoking on GrimAge using the smoking instrument comprised of variants in the CHRNA5-CHRNA3-CHRNB4 gene cluster eFigure 5. FUSION transcriptome-wide association study (TWAS) of lifetime smoking using the sparse canonical correlation GTEx weights eFigure 6. FUSION transcriptome-wide association study (TWAS) of lifetime smoking using weights from GTEx lung tissue eFigure 7. FUSION transcriptome-wide association study (TWAS) of lifetime smoking using whole blood gene expression (from GTEx) eFigure 8. FUSION transcriptome-wide association study (TWAS) of lifetime smoking using The Cancer Genome Atlas (TCGA) lung adenocarcinoma tissue eFigure 9. FUSION transcriptome-wide association study (TWAS) of lifetime smoking using The Cancer Genome Atlas (TCGA) lung squamous cell carcinoma tissue eFigure 10. Gene set enrichment results for smoking-associated genes eFigure 11. Adult organ system cell-type enrichment of the high-confidence genes associated with smoking behavior eFigure 12. Fetal organ system cell-type enrichment of the high-confidence genes associated with smoking behavior eFigure 13. Transcriptomic imputation comparison of the smoking-associated genes with epigenetic age acceleration eFigure 14. Correlations of the transciptomic imputation results for smoking, multivariate longevity, GrimAge, and PhenoAge eFigure 15. Correlations of the transciptomic imputation results for smoking, multivariate longevity, GrimAge, and PhenoAge eFigure 16. Correlations of the transciptomic imputation results for smoking, multivariate longevity, GrimAge, and PhenoAge eFigure 17. Potential models explaining SNV associations for neuropsychiatric disorders, substance use behaviors, and longevity endpoints eReferences

Supplement 2. eTable 1. Phenotype descriptions and sources eTable 2. Instruments for substance use and use disorders eTable 3. Instruments for schizophrenia, bipolar disorder, and major depression eTable 4. Finngen R10 neuropsychiatric exposure instruments eTable 5. Multivariable pairwise genetic instruments for lifetime smoking and substance use and DSMs eTable 6. Multivariable exposure instruments for DSMs and substance use* eTable 7. MVMR neuropsychiatric and substance use behavior instruments further adjusted for YrsSchooling, ModPhysAct, BMI, SleepDur, and SBP eTable 8. Multivariable genetic instruments for lifetime smoking and coronary artery disease, Type 2 diabetes, and obesity* eTable 9. Drug target exposure instruments for smoking eTable 10. Sample overlap eTable 11. Genetic correlations between phenotypes eTable 12. SVMR of substance use and DSM instruments on epigenetic clocks and multivariate longevity eTable 13. MR Lap analyses of substance use and DSM on multivariate longevity eTable 14. SVMR of Finngen neuropsychiatric instruments on epigenetic clocks and longevity eTable 15. SVMR of PheWAS screened exposures on epigenetic clocks and longevity eTable 16. SVMR of parental lifespan eTable 17. SVMR with removal of high variance SNVs to assess homogeneity assumption (see eMethods) eTable 18. SVMR CHRNA3-CHRNA5-CHRNA4 cis-located lifetime smoking instruments on multivariate longevity and epigenetic aging eTable 19. Colocalization analysis of the lifetime smoking-longevity and smoking-GrimAge relationships in the CHRNA3-CHRNA5-CHRNB4 locus eTable 20. MVMR pairwise analyses for lifetime smoking with alcohol use behaviors and DSMs on epigenetic clocks and multivariate longevity eTable 21. MVMR analyses of psychiatric disorders and substance use behaviors on epigenetic clocks and longevity eTable 22. MVMR analyses of Finngen Round 10 psychiatric disorders on epigenetic clocks and longevity eTable 23. MVMR analyses of psychiatric disorders and substance use behaviors on epigenetic clocks and longevity with adjustment for covariates eTable 24. MVMR analyses for lifetime smoking and CAD, T2D, and obesity on longevity eTable 25. SVMR analsyes of the psychiatric disorders and substance use behaviors on negtive controls to assess evidence of selection bias eTable 26. Full transcriptome-wide association study (TWAS) results on lifetime smoking using the FUSION method and cross-tissue GTEx weights eTable 27. Full transcriptome-wide association study (TWAS) results on lifestime smoking using the FUSION method and lung tissue (GTEx) eTable 28. Full transcriptome-wide association study (TWAS) results on lifestime smoking using the FUSION method and whole blood (GTEx) eTable 29. Full transcriptome-wide association study (TWAS) results on lifestime smoking using the FUSION method and squamous cell lung cancer eTable 30. Full transcriptome-wide association study (TWAS) results on lifetime smoking using the FUSION method and lung adenocarcinoma eTable 31. Transcriptomic imputation results for FUSION analysis on smoking behaviors (features surpassing correction for multiple comparisons [P-value < 8.31x10-7]) eTable 32. Transcriptomic imputation results of smoking-associated features (gene-tissue pairs) that surpass correction for multiple comparisions and colocalized with PP.H4 > 0.8 for all tissues analyzed (cross-tissue GTEx V8 weights, GTEx V8 whole blood, GTEx V8 lung, TGCA lung adenocarcinoma, and TGCA squamus cell lung cancer) eTable 33. Gene ontology and pathway enrichment for high-confidence smoking genes eTable 34. Cell-type enrichment results of the high-confidence genes assoicated with smoking behavior (TWAS P-values that surpassed correction for multiple comparisons and also demonstrated evidence of colocalization (PP.H4 >0.8) eTable 35. Open Gene database look up of TWAS genes associated with smoking that also colocalized at PP.H4 > 0.8 eTable 36. Comparision of high confidence smoking-associated genes (PP.H4 > 0.8 and Bonferroni correction) with aging-related outcomes in the cross-tissue GTEx weights eTable 37. Age-related gene expression differences for smoking-associated genes with colocalization PP.H4 > 0.8 and also demonstrating directionally consistent relationships with aging-related outcomes eTable 38. Cis-instrument Mendelian randomizaion screen of 1,901 cortical proteins on lifetime smoking behavior eTable 39. Druggability assessment of dlPFC proteins associated with smoking behavior surpassing correction for multiple comparisons eTable 40. Colocalization of cortical proteins associated with smoking behaviors surpassing correction for multiple comparisons eTable 41. Top cis-instrument MR results of cortical proteins associated with lifetime smoking behavior eTable 42. Genes available for replication analyses in whiole blood gene expression from the eQTLGen consortium eTable 43. Cis-MR analyses of the 18 proteins availabe for instrumentation in whole blood eTable 44. Results of Phenome-wide Mendelian randomization analyses screening impact of top cortical proteins associated with smoking behavior on 368 diseases and biomarkers

Supplement 3. Data sharing statement
