# Supplementary material for: Major Psychiatric Disorders, Substance Use Behaviors, and Longevity
Source: JAMA Psychiatry. 2024 Jun 18;81(9):889–901. doi: 10.1001/jamapsychiatry.2024.1429 (PMC11195603; doi:10.1001/jamapsychiatry.2024.1429)
Supplement: Supplement 1. — eMethods eResults eDiscussion eAppendix. STROBE-MR Reporting Guidelines eFigure 1. Mendelian Randomization Model and Assumptions eFigure 2. Mendelian Randomization (MR) Model and Assumptions applied to CHRNA5-CHRNA3-CHRNB4 gene cluster eFigure 3. Cross-trait genetic correlations between the psychiatric disorders and substance use behaviors used as Mendelian randomization exposures eFigure 4. SNV-SNV plot for Mendelian Randomization (MR) results assessing the relationship of smoking on GrimAge using the smoking instrument comprised of variants in the CHRNA5-CHRNA3-CHRNB4 gene cluster eFigure 5. FUSION transcriptome-wide association study (TWAS) of lifetime smoking using the sparse canonical correlation GTEx weights eFigure 6. FUSION transcriptome-wide association study (TWAS) of lifetime smoking using weights from GTEx lung tissue eFigure 7. FUSION transcriptome-wide association study (TWAS) of lifetime smoking using whole blood gene expression (from GTEx) eFigure 8. FUSION transcriptome-wide association study (TWAS) of lifetime smoking using The Cancer Genome Atlas (TCGA) lung adenocarcinoma tissue eFigure 9. FUSION transcriptome-wide association study (TWAS) of lifetime smoking using The Cancer Genome Atlas (TCGA) lung squamous cell carcinoma tissue eFigure 10. Gene set enrichment results for smoking-associated genes eFigure 11. Adult organ system cell-type enrichment of the high-confidence genes associated with smoking behavior eFigure 12. Fetal organ system cell-type enrichment of the high-confidence genes associated with smoking behavior eFigure 13. Transcriptomic imputation comparison of the smoking-associated genes with epigenetic age acceleration eFigure 14. Correlations of the transciptomic imputation results for smoking, multivariate longevity, GrimAge, and PhenoAge eFigure 15. Correlations of the transciptomic imputation results for smoking, multivariate longevity, GrimAge, and PhenoAge eFigure 16. Correlations of the transciptomic imputation results for s [file jamapsychiatry-e241429-s001.pdf]

Supplemental Online Content

Rosoff DB, Hamandi AM, Bell AS, et al. Major psychiatric disorders, substance use behaviors, and longevity. *JAMA Psychiatry*. Published online June 18, 2024. doi:10.1001/jamapsychiatry.2024.1429

eMethods

eResults

eDiscussion

eAppendix. STROBE-MR Reporting Guidelines

eFigure 1. Mendelian Randomization Model and Assumptions

eFigure 2. Mendelian Randomization (MR) Model and Assumptions applied to CHRNA5-CHRNA3-CHRNB4 gene cluster

eFigure 3. Cross-trait genetic correlations between the psychiatric disorders and substance use behaviors used as Mendelian randomization exposures

eFigure 4. SNV-SNV plot for Mendelian Randomization (MR) results assessing the relationship of smoking on GrimAge using the smoking instrument comprised of variants in the CHRNA5-CHRNA3-CHRNB4 gene cluster

eFigure 5. FUSION transcriptome-wide association study (TWAS) of lifetime smoking using the sparse canonical correlation GTEx weights

eFigure 6. FUSION transcriptome-wide association study (TWAS) of lifetime smoking using weights from GTEx lung tissue

eFigure 7. FUSION transcriptome-wide association study (TWAS) of lifetime smoking using whole blood gene expression (from GTEx)

eFigure 8. FUSION transcriptome-wide association study (TWAS) of lifetime smoking using The Cancer Genome Atlas (TCGA) lung adenocarcinoma tissue

eFigure 9. FUSION transcriptome-wide association study (TWAS) of lifetime smoking using The Cancer Genome Atlas (TCGA) lung squamous cell carcinoma tissue

eFigure 10. Gene set enrichment results for smoking-associated genes

© 2024 Rosoff DB et al. *JAMA Psychiatry.*

eFigure 11. Adult organ system cell-type enrichment of the high-confidence genes associated with smoking behavior

eFigure 12. Fetal organ system cell-type enrichment of the high-confidence genes associated with smoking behavior

eFigure 13. Transcriptomic imputation comparison of the smoking-associated genes with epigenetic age acceleration

eFigure 14. Correlations of the transciptomic imputation results for smoking, multivariate longevity, GrimAge, and PhenoAge

eFigure 15. Correlations of the transciptomic imputation results for smoking, multivariate longevity, GrimAge, and PhenoAge

eFigure 16. Correlations of the transciptomic imputation results for smoking, multivariate longevity, GrimAge, and PhenoAge

eFigure 17. Potential models explaining SNV associations for neuropsychiatric disorders, substance use behaviors, and longevity endpoints

eReferences

This supplemental material has been provided by the authors to give readers additional information about their work.

eMethods

Preregistration

These analyses were not preregistered.

Healthy aging, longevity, and epigenetic age acceleration background

Aging is a complex, multi-faceted process influenced by many factors.[1,2] Hallmarks of aging include the diminished maintenance of homeostatic mechanisms, resulting in age-related diseases, and death;[3] however, there exists substantial variability in aging,[3] that is not captured by conventional single-phenotype approaches to studying aging.[4] Additionally, while there has been an increase in overall lifespans, there has not been a corresponding increase in healthspan (considered the length of time considered free of chronic illness[5]), resulting in a growing healthspan-lifespan gap[6] that has, in part, motivated the general shift in the field of geroscience from a focus on increasing survival to incorporating complementary measures of age-related outcomes[4] with the goal of improving healthy aging (i.e., the maintenance of well-being in old age that includes both the absence of disease and also happiness, satisfaction, and fulfillment[7]). Given the complex relationship between healthspan (considered the length of time considered free of chronic illness[5]) and total lifespan, including the growing healthspan-lifespan gap,[6] we aimed to use an aging outcome that shared genetics underlying both healthspan and lifespan, to provide a broad genetic phenotype related to healthy aging processes.

To complement our primary healthy aging-related endpoint, we also evaluated the relationships of psychiatric disorders, substance use behaviors, and epigenetic age acceleration (EAA) as measured by epigenetic DNA methylation – epigenetic clocks (see refs.[8,9] for detailed description of epigenetic clocks). Briefly, epigenetic clocks are biomarkers used to estimate an individual's biological age based on chemical modifications to DNA (known as epigenetic changes). These clocks are derived from patterns of DNA methylation at specific sites across the genome and the underlying theory is that these epigenetic changes accumulate over time and can be used as a measure of aging, which may differ from chronological age, which is considered EAA.[8,9] Epigenetic clocks have shown promise in various fields of aging research and are used to study factors influencing aging, disease progression, and overall health outcomes. We included EAA because EAA has been found to be increased in psychiatric populations[3] and substance use behaviors,[10] and also because EAA has been shown to be reversible,[11] underscoring its potential as an endpoint to target for therapeutic development and measurable marker of potential prevention and intervention strategies, aimed, for example, at reducing aging and poor health in psychiatric populations due to smoking.

Data sources

Alcohol consumption exposure information. We obtained data from weekly alcohol consumption from the recent data from the GWAS & Sequencing Consortium of Alcohol and Nicotine use (GSCAN) meta-analysis (N=665 346), which adjusted for sex, age, age squared and genetic

principal components.[12] For problematic alcohol use (PAU), we used the Zhou et al. meta-analysis of AUD cases in the Million Veterans Program[13] and Psychiatric Genomics Consortium[14] with Alcohol Use Disorders Identification Test (AUDIT) Problem drinking questions (AUDIT-P, questions 7-10) derived from participants in the UK Biobank (N=435 563).[15] The PAU GWAS was adjusted for age, sex, and principal components.[13]

Multivariate aging outcome. We used a multivariate GWAS summary statistics from Timmers et al.[3] that was constructed to identify the genetics shared across derived from three univariate GWASs of European ancestry cohorts on healthspan (N=300 477),[5] parental lifespan (N=512 047 and 500 196 maternal and paternal lifespans, respectively),[1] and exceptional longevity (N=36 745).[16] See Timmers et al. for detailed information regarding the multivariate GWAS construction[3] and the original univariate GWAS manuscripts for additional details regarding the individual univariate GWASs. Briefly, the healthspan GWAS (N=300 477, UKB participants) was defined as the incidence of the 8 most common diseases in the UKB.[5] Cox-Gompertz survival models were calculated with clinical events in seven disease categories – cancer, cardiovascular disease, diabetes, stroke, and dementia – to determine length of healthspan. UKB participants reporting one or more of these events were considered to have completed healthspans. 84 949 UKB participants experienced an event, which completed their healthspans over the timeframe considered in the study.[5] The parental lifespan GWAS represented 512 047 and 500 196 maternal and paternal lifespans (also including UKB participants).[5] Across the meta-analyzed cohorts, Cox survival models for mothers and fathers were fitted and Martingale residuals of corresponding survival models were regressed against subject gene dosages to calculate the lifespan GWAS. We used summary statistics from Deelen et al. assessing the genetic underpinnings of exceptional old age using 11 262 unrelated participants reaching ≥90th survival percentile and performed a GWAS comparing this extreme longevity group to 25 483 participants whose age at death was ≤60th survival percentile.[16] Survival percentiles were based upon country-specific cohort life tables (e.g., the 90th survival percentile for the United States 1920 birth cohort is 89 years of age for men and 95 years of age for women and the 60th percentile is 75 and 83, respectively).[16]

Mendelian randomization instrumentation

Single variable Mendelian randomization (MR) instruments. For SVMR analyses, we included all exposure SNVs associated at conventional genome-wide significance (GWS) P-values$<5\times10^{-8}$ for all psychiatric and substance use-related exposures. All instrument SNVs were clumped at LD $R^2 = 0.001$ using reference samples comprised of participants of European ancestry.[17] See **eTables S2-S4** for all SVMR instruments, including the longevity instruments used in the bi-directional MR assessing whether the genetic predisposition for healthy aging and EAA impacts the risk for psychiatric disorders or impacts substance use behaviors. F-statistics for the unconditional instruments on average > 10 (**Tables S2-S4**), indicating minimal bias from weak instruments, which supports the plausibility of the first core MR assumption (i.e., the relevance assumption).[18,19]

Multivariable MR (MVMR) instruments. For the MVMR analyses, we concatenated independent instrument sets for psychiatric disorders, alcohol consumption, and lifetime smoking (clumping the resulting MV instrument sets to exclude intercorrelated SNVs with pairwise LD $R^2>0.001$,

© 2024 Rosoff DB et al. *JAMA Psychiatry.*

physical distance 10,000 kb). We created overall MVMR instruments comprised of each psychiatric disorder, smoking, and one of the alcohol use behaviors. Given the strong genetic correlation between PAU and DPW (**Figure 1c**), we did not incorporate those exposures into the same MVMR instrument and created separate MVMR instruments using smoking + DPW + the psychiatric disorders (not including PAU); and smoking + PAU + the psychiatric disorders. We also constructed MVMR instruments assessing each of the psychiatric disorders and alcohol use behaviors, in turn, with smoking behavior (e.g., smoking + SCZ, smoking + MDD, smoking + BD, etc.). We were unable to calculate conditional F-statistics to assess the strength of the multivariable instrument sets; SVMR statistical methods recently extended to two sample MVMR are only appropriate for non-overlapping exposure summary level data sources. When overlapping, the requisite pairwise covariances between SNV associations are only determinable using individual level data.[20] MVMR instruments are presented in **eTables 5-7**.

Additional MVMR models testing the robustness of smoking findings. We aimed to evaluate the robustness of the main smoking-related findings by including additional MVMR models accounting for cardiometabolic risk factors: obesity, coronary heart disease (CAD), and Type 2 Diabetes (T2D), which have each been previously associated with both smoking and longevity[21-23] and may, therefore, mediate the relationship of smoking and longevity. For obesity, we used three GWASs from GIANT (Genetic Investigation of ANthropometric Traits)[24]; for CAD genetics, we used the CARDIoGRAMplusC4D-UK Biobank CAD, Coronary ARtery DIsease Genome wide Replication and Meta-analysis (CARDIoGRAM), plus The Coronary Artery Disease (C4D) Genetics GWAS meta-analysis.[25] T2D data came from a recent meta-analysis of three T2D studies: DIAbetes Genetics Replication and Meta-analysis (DIAGRAM), Genetic Epidemiology Research on Aging (GERA), and the full cohort release of UKB.[26] MVMR instruments concatenating obesity, CAD and T2D with smoking were constructed by excluding intercorrelated SNVs with pairwise LD R^2>0.001 (physical distance 10 000 kb) (**eTable 7**).

Replication with psychiatric disorder diagnoses in the FinnGen cohort. We also included replication analyses with the latest public release from the FinnGen cohort[27] (Release 10, N=412 181; 230 310 females and 181 871 males). See the original publication for comprehensive cohort information,[27] but briefly, the FinnGen project is a collaborative research initiative that merges imputed genotype data from new and existing samples from Finnish biobanks with digital health records from national health registries in Finland (with details provided at https://www.finngen.fi/en).[27] FinnGen uses data sources from a comprehensive, nationwide health registry with health information of all residents in Finland since 1969. FinnGen Release 10 (made public on December 18, 2023) analyzes 2 408 electronic-health record related endpoints and its GWAS data has 21 311 942 SNVs for analysis.

This data was not included in the psychiatric disorder GWAS meta-analyses that were used as the primary exposures in this study. Therefore, we performed replication analyses using FinnGen cohort GWASs of ICD-based diagnoses of schizophrenia (6 708 cases; 398 386 controls), bipolar disorder (7 569 cases; 359 290 controls), major depression (47 696 cases; 359 290 controls), and AUD (17 197 cases; 394 984 controls) (**eTable 1**). We instrumented these exposures as described above except for these exposures we used a P-value threshold of 5×10^{-6} due to there being only few genome-wide significant SNVs. We ensured sufficient instrument strength and only included SNVs with F-statistics exceeding the conventional threshold of 10.

© 2024 Rosoff DB et al. *JAMA Psychiatry.*

As the FinnGen data is based upon electronic health records, it does not have outcomes related to lifetime smoking behavior or weekly alcohol consumption. Therefore, we were unable to perform replication of these exposures.

We performed SVMR and MVMR using these FinnGen exposures. In addition to assessing the robustness of the SVMR and MVMR of the, the FinnGen data was also used to assess potential issues related to the sample construction of the primary exposures derived from the psychiatric disorder GWAS meta-analyses, including the inclusion of retrospective case-cohorts, selection bias, and the same population assumption for two-sample MR analyses (each described in the **MR statistical and analyses** section below).

Instrumentation of the CHRNA5-CHRNA3-CHRNB4 gene cluster. In addition to evaluating the relationships of lifetime smoking behavior on healthy aging and epigenetic clocks using complementary MR to assess the MR assumptions,[19] we also performed MR[28] analyses using variants located within or near the *CHRNA5-CHRNA3-CHRNB4* gene cluster on chromosome 15 (78,857,862—79,020,096) that encode the nicotinic acetylcholine receptors (nAChRs) and have been shown to impact smoking behaviors, including smoking heaviness and smoking dependence.[29] Given the cis-acting nature of these variants and the well-known impact of *CHRNA5-CHRNA3-CHRNB4* gene cluster on the physiological effects of nicotine (i.e., nicotine produces its effects by binding to nAChRs[30]) and smoking behaviors, these MR analyses using variants within a locus of known biological function are unlikely to be influenced by common confounders.[29] Further, as there are challenges regarding MR instrumentation in studies investigating neuropsychiatric disorders and behaviors because the mechanisms through which the variants impact the exposures are frequently unknown[31,32] (i.e., versus biomarkers such as circulating lipids). Therefore, performing MR analysis of smoking on aging-related outcomes using variants located within or near the *CHRNA5-CHRNA3-CHRNB4* gene cluster provides additional support for the primary two-sample MR analyses using the polygenic smoking instrument comprised of variants located throughout the genome. We used conventional instrumentation methods and extracted variants (with lifetime smoking association statistic P-values$<5\times10^{-8}$, LD $R^2<0.1$) located within 100 kilobases of the *CHRNA5-CHRNA3-CHRNB4* gene cluster and identified 5 cis-variants for harmonization with the aging related outcomes.

Cis-instrument MR for proteomic screen of potential smoking-cessation drug targets. We used protein quantitative trait loci (pQTL) data derived from the three cortical regions (dorsolateral prefrontal cortex, orbitofrontal cortex, and parahippocampal gyrus) of 722 participants of European descent included in the ROSMAP cohort[33] to generate SNV-protein instruments necessary for MR analysis. pQTL data was filtered for GWS SNVs at $P < 5\times10^{-8}$. *Cis*-instruments were created for each protein using variants within 100 kilobases of the protein's encoding gene, giving 1,909 SNV-protein instruments. We filtered variants using the 1000 Genomes Project Phase 3 European reference panel[34] and clumped the variants at LD) $R^2<0.1$ (10,000 kb window). F-statistics used assess the MR relevance assumption by evaluating instrument strength. We only included variants with F-statistics ≥10, the conventional cutoff for determining the variant is sufficiently strong and will be unlikely to be subject to weak instrument bias.[18] Instrument SNVs were extracted from the smoking GWAS summary statistics and association directions between exposure and outcome where matched to the same allele. pQTL instrument data are presented in **eTable 9**.

© 2024 Rosoff DB et al. *JAMA Psychiatry.*

MR statistical and sensitivity analyses

MR analyses were carried out using TwoSampleMR, version 0.5.5,[31] MendelianRandomization, version 0.5.0,[35] in the R environment, version 4.0.2.

MR assumptions. Two-sample MR uses common SNVs derived from GWAS data as instrumental variables to evaluate associations between the genetic liability for an exposures-outcome pairs of interest.[36-38] MR analyses are subject to the core MR assumptions: (1) relevance, (2) independence, and (3) exclusion restriction.[39] The relevance assumption assumes that genetic variants used as instruments are strongly associated with the exposure. The independence assumption necessitates that these variants are independent of confounders of the exposure-outcome relationship. Finally, the exclusion restriction criterion assumption ensures that the variants affect the outcome solely through the exposure.[39] By employing a comprehensive suite of MR methods, each with assumptions and strengths, our analysis aims to provide a robust and nuanced understanding of the causal relationships between genetic liabilities for the psychiatric and substance use risk factors and longevity-related outcomes. This multifaceted approach allows us to account for and evaluate the influence of potential violations of MR assumptions on our findings.

We tested the relevance assumption by calculating the variance explained by the instrument (R^2) and F-statistics for each SNV comprising the exposures instruments. By convention, if SNVs with F-statistics >10 are used for instrument construction, the resultant instruments are unlikely to be subject to weak instrument bias, which may occur when the variants comprising the MR instrument explain only a small proportion of the exposure resulting in reduced statistical power to reject the null hypothesis.[40] As detailed in the **Results** section in the main manuscript, our psychiatric and substance use instruments, estimated F-statistics for the unconditional instruments generally exceeded 20 (F-statistic = 10 being the conventional threshold designating weak and strong MR SNV instruments[40]), which provides evidence that the psychiatric and substance use instruments are unlikely to be subject to bias from weak instruments.[41]

We used inverse-variance weighted MR (IVW) as the main estimates for the SVMR and MVMR. To assess the sensitivity of our analyses to potential violations of these IV assumptions and to validate the robustness of our results, we supplemented IVW with several complementary and robust techniques: MR-Egger, weighted median, penalized weighted median, and weighted mode methods. MR-Egger allows for the detection and correction of pleiotropic effects, where genetic variants might affect the outcome through pathways other than the exposure. The weighted median and mode methods offer consistent causal estimates under weaker assumptions, providing resilience against invalid instruments. Consistency of results across methods strengthens an inference of causality.[31] In the following paragraphs, we describe in more detail these complementary MR methods.

MR Egger extends the MR IVW method, allowing for the net horizontal pleiotropic MR estimate across all SNVs to be unbalanced or directional, i.e., some SNVs may be acting on the outcome via one or more pathways other than through the exposure by not fixing the intercept to the origin.[42,43] Put another way, MR Egger complements the MR IVW method by allowing for the

© 2024 Rosoff DB et al. *JAMA Psychiatry.*

net horizontal pleiotropic MR estimate across all SNVs to exhibit an unbalanced or directional nature. The relaxed assumption of MR Egger, referred to as the "Instrument Strength Independent of Direct Effect" (InSIDE) assumption, offers flexibility compared to the strict MR assumption of no pleiotropy (it may be considered a less strict version of the exclusion restriction MR assumption), which means that the magnitude of the pleiotropic effects is not correlated with the SNV-exposure associations for the genetic variants comprising the instrument of the exposure.[42,43] For more information on the InSIDE assumption we refer the interested reader to refs.[42,43] MR Egger provides unbiased MR estimates even when the MR assumption of no horizontal pleiotropy is violated for all SNVs. However, it is important to note that the precision of these estimates may be significantly lower compared to MR IVW: while the precision of the IVW estimate is influenced by the proportion of variance in the exposure explained by the genetic instruments (R^2), MR Egger's precision is contingent on the variability observed in the SNV-exposure associations.[42,43]

The weighted median MR method utilizes the median associations of all available instrumental SNVs, making it necessary for only half of the SNVs to qualify as valid instruments. Valid instruments are defined as variants that exhibit no horizontal pleiotropy, have no associations with confounding factors, and demonstrate a strong association with the exposure. This approach ensures an unbiased MR estimate.[44] Additionally, in the weighted median MR method, the influence of stronger SNVs on the estimate is greater, and each SNV's contribution is weighted by the inverse variance of its association with the outcome.[44]

The weighted mode-based MR method groups SNVs based on the similarity of their MR associations and calculates the MR estimate based on the cluster with the largest number of SNVs. In this method, the MR estimate remains unbiased as long as the SNVs within the largest cluster are valid instruments.[31] It assumes that the most common relationship is consistent with the true underlying relationship[45] and assigns weights to each variant for clustering based on the inverse variance of its association with the outcome. Assuming that the most common MR estimate is consistent, this approach ensures an overall unbiased MR relationship, even when all other instruments are invalid.[45]

While the conventional MR IVW method can yield biased results if even a minority of the instruments are invalid, the penalized weighted median method addresses a key challenge in MR studies: inference given the presence of invalid IVs, which are SNVs that violate MR assumptions, often due to pleiotropy.[44] The penalized weighted median enhances this weighted median method by incorporating a penalty for invalid IVs, effectively downweighting their influence in the MR analysis.[44] This penalization is usually based on a measure of heterogeneity or pleiotropy among the genetic instruments, and by applying this penalty, the method aims to minimize the impact of potentially invalid instruments on the final causal estimate.[44] The penalized weighted median method is particularly useful in scenarios where there may be invalid instrument SNVs, or the increased potential for pleiotropy (as may be suspected in genetic instruments for psychiatric outcomes); and by accounting for and mitigating the influence of pleiotropic SNVs, it represents an important method to include in the robustness and reliability of MR analyses.[44] The penalized weighted median estimator downweights genetic variants with heterogeneous estimates, reducing their impact on the analysis using Cochran's Q statistic, which measures heterogeneity between estimates. SNVs that significantly deviate from the group

© 2024 Rosoff DB et al. *JAMA Psychiatry.*

(indicated by a high Q value) are penalized more heavily. The penalization involves multiplying the variant's weight by a factor determined by its P-value: if the P-value is low (indicating high heterogeneity), the weight is significantly reduced. This approach ensures that most variants remain unaffected unless they show significant heterogeneity, in which case their influence on the estimate is substantially diminished.[44]

The weighted mode MR method clusters SNVs comprising the genetic instruments based on the similarity of their MR associations.[46] After clustering SNVs, it calculates the MR estimate by considering the cluster that contains the largest number of SNVs. This approach provides a robust MR estimate, unbiased as long as the SNVs within the largest cluster are valid instruments. Weighting for clustering is determined by the inverse variance of each variant's association with the outcome, ensuring a reliable MR relationship even when other instruments may be invalid.[46]

Heterogeneity and directionality tests. We used the MR Egger intercept test,[47] Cochran Q heterogeneity test,[48] and multivariable extensions thereof, to evaluate heterogeneity in instrument effects: heterogeneity may indicate violations of MR assumptions.[47,49,50] The MR Egger intercept test facilitates detection of pleiotropy, where genetic variants affect the outcome through pathways other than the exposure of interest: pleiotropy can bias MR estimates. The Cochran Q test assesses the variability in effect sizes across different genetic instruments, identifying potential outliers or inconsistent instruments that might violate the IV assumptions.

Additionally, we employed the SVMR Steiger directionality test[17] to test the causal direction between selected exposure and outcomes to help determine whether the genetic associations of the instrument SNVs are more likely to reflect the impact of the exposure on the outcome, rather than the reverse.[17] We removed SNVs from the exposure instruments failing the Steiger directionality test, which establishes the correct direction of causation for the exposure-outcome pairing, strengthening the validity of the MR findings.

MR LASSO. For both SVMR and MVMR analyses, the MR LASSO method[51] was utilized to enhance the robustness and validity of our causal estimates. MR LASSO applies lasso-type penalization to the direct effects of the exposure instruments on the outcome under analysis; this penalization is effective in refining the selection of genetic instruments, which facilitates addressing the IV relevance assumption.[51] MR LASSO attenuates the influence of weaker instruments that might not have a strong association with the exposure, or might be invalid due to pleiotropic effects; by focusing on stronger and more valid instruments, the MR LASSO method reduces the risk of bias in the causal estimates.[51] The "post-lasso" estimate, obtained by IVW using only those SNVs identified as valid, represents a more refined causal estimate, important in studies using genetic instruments, where the risk of including invalid instruments is increased.[28] However, a Cochran Q test indicating heterogeneity (Cochran's Q P-value < 0.05), notwithstanding, the MR LASSO may not identify outlier SNVs, in which case, the post-lasso MR estimate is identical to the initial IVW estimate.

For additional information regarding the MR LASSO method, we refer the interested reader to ref.[51] MR LASSO modifies the IVW model by incorporating an intercept term for each genetic variant; these intercepts signify the direct associations between the genetic variants and the

outcome, independent of the risk factor. MR estimates are obtained through weighted linear regression, applying lasso-type penalization to the intercept terms: this penalization generally reduces the intercepts of valid instruments to zero, which is effective in refining the selection of genetic instruments, which facilitates addressing the IV relevance assumption.[51] The extent of this penalization is governed by a tuning parameter that determines the level of sparsity: a heterogeneity stopping rule is the default setting for this parameter. Genetic variants are reoriented so that all associations with the risk factor are positive; the signs of associations with the outcome are adjusted to maintain this consistent orientation, which is automatically handled by MR LASSO.[51] MR LASSO proceeds in two stages: first, fitting a regularized regression model to identify valid genetic instruments, and then estimating the causal effect using IVW with these valid instruments, generating the post-lasso estimate.

MVMR analysis. In MVMR analyses, we used multivariable extensions of the IVW, MR Egger, and MR median methods[49,52] to evaluate the direct effects of each psychiatric and substance use risk factor on longevity outcomes, simultaneously considering multiple exposures, thereby accounting for potential confounding among the exposures.

MVMR assumptions. MVMR is a method used in genetic epidemiology to understand the causal relationships between multiple exposures (e.g., lifestyle factors, environmental exposures) and an outcome.[53,54] This approach relies on the use of genetic variants, typically SNVs as instrumental variables (IVs). For MVMR to be valid, it is important that these SNVs are associated with the exposure variables but do not influence the outcome directly, except through these variables. Like single-variable MR, MVMR utilizes SNVs to evaluate the exposure variables, and these predictions are then used in a multivariable regression analysis to estimate the effects of the exposures on the outcome. MVMR is based on three core IV assumptions:[53]

1. **MVMR Assumption 1**: IVs must predict the exposures, conditioned on other exposures in the model (so as to preclude multicollinearity and weak instrument bias).

2. **MVMR Assumption 2:** IVs must be independent of the outcome, given all the exposures in the model, i.e., SNVs should influence the outcome only through their effect on the exposures, and not directly.

3. **MVMR Assumption 3**: IVs should be independent of any confounders of the relationship between exposures and outcome (so as to ensure that estimated effects are not biased due to confounding factors).

MVMR allows for the inclusion of SNVs that affect multiple phenotypes (pleiotropy), as long as those phenotypes are included as exposures in the analysis.[53,54] Thus MVMR is less restrictive than univariable MR. However, heterogeneity in SNV-outcome associations is still evaluated to detect potential unaccounted pleiotropy; the multivariable extensions of the Cochran Q statistic is used to assess for pleiotropy, and the multivariable extension of MR Egger, to assess directional pleiotropy.[54]

Homogeneity assumption. As long as the three core MR assumptions are satisfied, i.e., (1) relevance, (2) independence, and (3) exclusion restriction, MR can be used to test the sharp null

hypothesis that the exposure does not have an impact on the outcome for any individual in the population.[19,39] However, an additional assumption – homogeneity – is required for point estimation of the average causal effect (ACE) in MR analyses.[19,55,56] Homogeneity implies that the causal relationship of the exposure on the outcome is constant across different levels of an unmeasured confounder. If this homogeneity assumption does not hold, while not likely to lead to the inappropriate causal inference from the MR analyses (or inflated type 1 error rates),[57] the estimated average causal effect may not accurately reflect the true causal effect in the general population.[55] For instance, in the context of the MR analyses in this study, the neuropsychiatric disorders and longevity, factors like chronic diseases or socioeconomic status could vary in their influence on different individuals, which would violate the homogeneity assumption.

To assess the plausibility of the homogeneity assumption, we used a sensitivity analysis suggested by Matthew et al. (2022): for each exposure, SNVs are ranked by instrument-exposure variance, then revised instrument sets (with reduced potential for heterogeneous effects) are constructed removing, in turn, 5%, 10%, 25%, 50%, and 75% of the total instrument SNVs.[58] We ensured that the average F-statistic of the revised instrument sets were still strong, and repeated the SVMR analyses on longevity and the epigenetic clock outcome for each of these revised instrument sets, so as to assess whether the MR estimates were robust across and directionally consistent the primary and revised instrument sets, providing evidence against violations of the homogeneity assumption.[58]

Specific application to the CHRNA5-CHRNA3-CHRNB gene cluster. As described above in the subsection "*Instrumentation of the CHRNA5-CHRNA3-CHRNB4 gene cluster*", we performed sensitivity analyses using a cis-instrument MR approach[28] to further address the MR assumption of no pleiotropy. In examining the *CHRNA5-CHRNA3-CHRNB4* gene cluster, we applied the IVW and complementary MR methods (described above), ensuring a consistent approach across MR analyses.

Additional sensitivity to assess the MR exclusion restriction assumption. In addition to using MR methods that identify pleiotropic SNVs, we also performed sensitivity analyses with genetic instruments that have been screened to identify SNV associations with other GWAS traits (which SNPv may violate the core MR exclusion restriction assumption, i.e., the "no pleiotropy" assumption).[19] Using Phenoscanner V2[59] (a curated database of publicly available GWAS results (>65 billion SNV-phenotype associations for >150 million SNVs), we screened SNVs instrumenting the psychiatric disorders and substance use behaviors to evaluate whether selected SNVs included in each genetic risk score had been associated with traits (other than the exposure of interest) in previous GWASs to minimize the potential for pleiotropic effects that could confound our results. We removed any SNVs from the instruments if the SNVs demonstrated genome-wide significant associations (P-value $< 5\times10^{-8}$) with other GWASs in the Phenoscanner V2 database and repeated the SVMR analyses on the multivariate longevity and epigenetic aging outcomes.

In addition, we conducted additional MVMR analyses that included adjustment for educational attainment, body mass index, sleep duration, physical activity, and systolic blood pressure from GWASs of European ancestry (see **eTable 1** for data source information) to further assess evidence of pleiotropy. These MVMR models were constructed using the methods described

above and simultaneously evaluated the impact of the psychiatric disorders, substance use behaviors, and additional covariates on the multivariate longevity outcome of biological aging.

Sample independence. A comprehensive overview of the cohorts included in the GWAS is presented in **eTable 10**. The percent overlap, calculated for purposes of providing insight into potential bias, was calculated according to the methodology outlined by Burgess et al.,[18] i.e., ratio of maximum potential overlap to the larger of the exposure or outcome GWAS. For DPW-epigenetic aging, 1.1% overlap was observed; drinks per week-multivariate longevity exhibits a 5.72% for diverse non-UK Biobank (UKB) cohorts, and for the UKB cohort, 51.1% overlap. Similarly, bipolar disorder-multivariate longevity displays 1.08% overlap for non-UKB cohorts, and 8.39% UKB cohort overlap. Major depression-multivariate longevity shows 5.83% overlap for non-UKB cohorts and 50.9% UKB cohort overlap. Lifetime smoking- multivariate longevity demonstrates a 65.2% overlap of the UKB cohort.

In two-sample MR, sample overlap between exposure and outcome datasets produce may bias IVW estimates.[60] It may also impact two other sources of potential bias in MR IVW estimates, namely weak instrument bias and winner's curse.[61] Winner's curse may occur when the same sample used to select the instruments is used as the exposure dataset.[61] It has been shown in simulation studies that sample overlap bias is minimal when the variants comprising the MR instruments are strong (i.e., the variants have large F-statistics [F-statistics >10][60]), and when overlapping samples come from large biobanks (i.e., the UKB),[62] suggesting that our results are minimally affected by this source of bias. Therefore, given that the potential overlap between the exposures and outcomes are primarily from data sourced in the UKB and the instruments comprising the substance use and neuropsychiatric exposures are strong, we do not expect sample overlap to bias these analyses.

For the primary psychiatric disorder and substance use behavior exposures, we use the MRLap method recently developed to account for sample overlap between exposures and outcomes in two-sample MR analyses and shown to robust in settings when the exact overlap percentage is unknown.[61] Assuming a "spike-and-slab" genetic architecture of the exposures, MRLap leverages cross-trait LD-score regression and calculates approximate sample overlap to provide an IVW estimate corrected for sample overlap.[63] MRLap also assesses weak instrument bias and winner's curse.[61] We performed MRLap for all of the exposure-outcome analyses in the single-variable MR, and per the developer guidelines, we use MRLap as a sensitivity analysis and report the MRLap-corrected estimate if it was different than the single variable IVW estimate.

For schizophrenia, bipolar disorder, major depression, and alcohol use disorder, we also repeated the main analyses with independent, non-overlapping GWAS data (i.e., GWAS data that were not included in the meta-analyses for the main psychiatric disorder exposures did not overlap with multivariate longevity outcomes or epigenetic clock GWASs) from the FinnGen release 10 (described in the above sections). FinnGen does not have outcomes related to weekly alcohol consumption or smoking behavior, and therefore, these exposures were not included in the replication.

Selection bias for MR analyses. Large-scale GWAS analyses frequently uncover minor yet significant correlations with various common genetic variants using patient or volunteer-based

© 2024 Rosoff DB et al. *JAMA Psychiatry.*

cohorts, leading to the development of polygenic risk scores (PRS) amalgamating these associations into weighted summary scores.[64] Despite these advancements, all genetics based studies, including in the field of psychiatry, face important methodological challenges, including the potential for selection bias in the GWAS samples.[64] Selection bias occurs in genetic studies when participants included in the study are not representative of the general population, which may happen if the method of participant selection is biased or if certain groups are over- or under-represented.[64] For example, if a genetic study on a particular disease only includes hospital patients, it might miss genetic variants that are relevant in the general population or overestimate the association between certain variants and the disease because hospital patients might have more severe forms of the disease.[64]

It has been shown that the UK Biobank is subject to selection bias (in particular a "healthy-volunteer bias") among its participants: only 5.5% of the approximately 9 million UK adults invited to participate enrolled in the prospective cohorts study,[65] and the participants have been shown to be more educated and have healthier lifestyles than the general UK population.[66] Similarly, the MVP is a cohort of US military veterans in the Veterans Health Administration healthcare system,[67] which by its design is subject to selection bias.

We performed a series of sensitivity analyses to assess evidence for selection bias in this study. First, we conducted extended MVMR with variables that may predict selection, including educational attainment, which has been recommended as one approach to assess evidence of selection bias,[68] and performed repeated analyses using independent psychiatric exposure data from the FinnGen cohort. We also performed negative control analyses where the assumption is made that the exposure(s) of interest cannot impact the outcome, which analyses has been previously recommended to evaluate selection bias in observational and two-sample MR studies.[68,69] Here, we performed SVMR for the psychiatric disorders and substance use behaviors on self-reported tanning ability and also skin color; tanning ability and hair color have been used previously as example outcomes to detect population stratification in two-sample MR and suggested as a sensitivity analysis for selection bias (as discussed by Sanderson et al.[70], while selection bias and population stratification are different, their resulting biases for two-sample MR analyses are similar[70]) because the traits are determined at birth and not expected to be truly affected by any of the psychiatric disorders or substance use behaviors evaluated in this study.[70] We performed negative control analyses using the Open GWAS Project GWAS of self-reported ease of tanning and skin color among UK Biobank participants of European ancestry (N (tanning) = 453 065, N (skin color) = 456 692).[71] We considered any MR IVW estimate with P-value < 0.05 as evidence of an association between the exposure and self-reported tanning ability and/or skin color, which would suggest the exposure is subject to selection bias, and P-values > 0.05 as evidence against the presence of selection bias.

Additional two-sample MR assumptions. Two-sample MR has several additional assumptions beyond the three main IV assumptions discussed above. These assumptions include that the association between the genetic IV and exposure (here psychiatric disorders and substance use behaviors) and the association between the genetic IV and outcome (here longevity and biological age acceleration, are derived from comparable underlying populations.[72] This issue is particularly evident in settings such as those testing the Developmental Origins of Health and Disease (DOHaD) hypothesis,[72] using a sex-combined exposure GWAS (e.g., GWAS of

circulating lipids which assumes that the genetic instrument-exposure relationship is consistent across both males and females) performed against sex-specific outcome (e.g., the genetic liability for breast cancer in women), or performing trans-ancestral MR using exposures and outcome GWASs of non-matching ancestries.[72]

While our current study uses only data from participants of European ancestry and performs no sex-stratified analyses (both due to limited availability of either exposure or outcome data, or both), we have included analyses from participants of European ancestry that are taken from cohorts with different sample demographics, e.g., analyses of the multivariate longevity outcome predominantly derived from the UK Biobank cohort (a large prospective study with ~500 000 participants aged 40–69 years at the age of recruitment)[73] with the PAU GWAS (exposure) data, derived from both UK Biobank and also the Million Veteran Program (MVP) cohorts, the latter being a cohort (predominantly male) of US military veterans using the US Veterans Health Administration healthcare system.[67] While age and sex were included, along with population principal components, as covariates in the linear models for the GWASs, there may still be remaining impact on the MR estimates as a result of potentially violating the two-sample MR assumption that the instrument-exposure and instrument-outcome associations are derived from comparable underlying populations.[72]

To address this potential source of bias, we performed several sensitivity analyses. First, for the psychiatric exposures, we performed replication analyses using the FinnGen cohort (described above). Additionally, for the PAU exposure using the MVP data, we evaluated whether the trait was genetically related to the other UK Biobank-derived alcohol consumption exposure traits, which would suggest a similar underlying genetic architecture (**eFigure 3**).

Additional details necessary for interpreting study findings

In the following subsections we discuss additional MR assumptions and related aspects of the analyses that are important for interpreting the study findings.

Consistency assumption. Another assumption for MR studies is the gene-environmental equivalence assumption (the "consistency assumption"),[74] i.e., the genetically-mimicked levels of the exposure represent the changes observed if there were direct interventions on the exposure.[75,76] Only the first IV assumption (the relevance assumption) in MR is fully empirically testable, while both the other two IV assumptions (independence and exclusion restriction), each rely upon all possible confounders of the exposure-outcome association (both measured and unmeasured) making it not possible to empirically validate the assumptions; however, it is possible to empirically assess the plausibility of these assumptions with complementary MR methods, sensitivity tests, e.g., negative controls, MVMR, and screening for pleiotropic variants. It is not currently possible to empirically assess the plausibility of the consistency assumption. More specifically, for this study the psychiatric disorders like schizophrenia, bipolar disorder, and major depression are complex and multifactorial,[77] with different potential interventions (e.g., pharmaceutical, behavioral, environmental) that might either induce or prevent these disorders could have varying impacts on longevity and biological aging. Given that the broad genetic liabilities underlying these disorders are not able to distinguish intervention-specific differences, there may be deviations of the MR results due to

© 2024 Rosoff DB et al. *JAMA Psychiatry.*

the consistency assumption. Further, these genetic liabilities likely do not capture the complexity and variability of treatments and outcomes in actual clinical settings, suggesting that the estimated effect might not align with the effect of any specific, real-world intervention. As there are currently no sufficiently well-powered GWAS data that would enable an intervention-specific analysis for these psychiatric outcomes due to trait complexity, and given the primary goal of this study to use the results from the SVMR and MVMR to guide further multi-omics analyses, we underscore interpretation of these analyses following the current recommended guidelines by Burgess et al.[74]: the genetic instruments for the psychiatric disorders do not reflect specific interventions but instead are used to find the set of genetic instruments that plausibly satisfy the instrumental variable assumptions (to the extent possible with the collection of complementary MR methods, sensitivity analyses, and MVMR models) and evaluate their relationships with longevity and biological aging using the largest available datasets.[74]

Case-control cohorts. It has been shown that MR using data from retrospective case-control studies may generate estimates subject to ascertainment bias (where the retrospective cohort is ascertained upon disease status).[78] While the GWAS data sourced for this study were derived primarily from large meta-analyses with the majority of the included cohorts coming from prospective large biobank data (i.e., UK Biobank, MVP, and FinnGen), several of the smaller studies contributing to these meta-analyses were derived from retrospective case-control analyses (e.g., the Collaborative Study on the Genetics of Alcoholism [COGA] study[79] included in the PAU meta-analysis by Zhou et al.[15] and ~6 000 of the ~25 400 comprising the exceptional longevity GWAS[80] included in the larger multivariate longevity outcome [N=709 709]).

We sourced these GWAS meta-analyses as the largest-available GWASs for the traits. However, we have incorporated several sensitivity analyses aimed at addressing potential biases related to inclusion of the retrospective case-control studies in the GWAS meta-analyses, including replication of the analyses using GWAS that do not include retrospective case-control cohorts (i.e., the FinnGen Release 10 diagnoses of schizophrenia, bipolar disorder, major depression, and alcohol use disorder), and comparing the main multivariate longevity estimates with analyses assessing the impact of the exposures on the univariate GWAS of lifespan, which does not include case-control cohorts.

Assessing potential collider bias due to heritable covariate adjustment for the included GWASs. Adjusting for heritable covariates (e.g., body mass index) in GWAS construction may bias MR studies if the heritable covariate is a collider for the trait under evaluation.[81] For example, Hartwig et al.[81] demonstrated in simulations that covariate adjustment in the source GWAS data for two-sample MR studies may eliminate bias from horizontal pleiotropy, but also result in bias when there is residual confounding.[81] The GWAS summary statistics sourced for this study only adjusted for age, sex, and principal components, which are the conventional covariates in GWAS analysis[82] and did not include additional heritable covariates in their construction, which suggests minimal potential for this source of bias in these analyses. See **eTable 1** for additional details regarding covariate adjustment for the included GWASs.

Time-varying exposures in MR studies. In MR, SNVs comprising the exposure instruments are generally assumed to be consistently related to the exposure throughout an individual's life. However, in reality, the influence of these genetic variants on behaviors or conditions like

© 2024 Rosoff DB et al. *JAMA Psychiatry.*

substance use or psychiatric disorders can fluctuate over time, resulting in potentially biased MR estimates if the genetic instruments' relationship to the exposures changes over time.[83] Additionally, while the use of MVMR allows for the consideration of multiple exposures simultaneously, it does not inherently correct for this issue, and MVMR may also produce biased estimates if the gene-exposure relationships are not constant over time or if the exposures interact with each other.[84,85] This complexity further complicates the interpretation of MR findings in the context of time-varying exposures like psychiatric disorders or substance use behaviors. Therefore, we underscore the importance of interpreting the estimates for these MR analyses as reflecting the genetic liability (and lifelong risk associated with them) for the analyzed psychiatric disorders and substance use behaviors rather than the effect of the exposures themselves. Put another way, we emphasize that these MR exposures reflect the innate genetic predisposition to their respective psychiatric disorders and substance use behaviors, rather than their direct impact, and estimating the impact of these genetic liabilities differs from estimating the impact of the exposure itself: the former relates to an individual's inherent risk, while the latter pertains to the actual impact of the behavior or condition.[83,85]

Interpretation of MVMR results. In our final MVMR models simultaneously adjusting for multiple variables such as the genetic liabilities for smoking, major depression, schizophrenia, bipolar disorder, and weekly alcohol consumption, each variable included serves as a covariate to account for potential confounding effects. The primary aim of including these variables in the fully adjusted models is to control for potential confounding factors.[54] Adjusting for these variables, we aim to isolate the specific causal relationship between the exposures (e.g., a genetic variant associated with a particular trait) and outcomes, minimizing the influence of other factors that could independently affect the outcome.[54]

Importantly, when adjusting for multiple traits, it is essential to consider pleiotropy (i.e., a single genetic variant affecting multiple traits).[47] In MVMR, the inclusion of multiple traits as covariates facilitates identification and accounts for pleiotropic effects, which improves inferences that the estimated causal effect is specific to the exposure of interest.[54] Therefore, the MVMR estimates associated with the exposure variables in the MVMR model may be interpreted as the direct causal role after accounting for the specified covariates.[54] In other words, these coefficients represent the estimated causal impact of the genetic variants on the longevity outcomes, independent of the potential confounding effects of the other exposures included in the MVMR models, e.g., the role of genetically predisposed smoking, independent of major depression, schizophrenia, bipolar disorder, and alcohol consumption.

Additional methods information for the cis-instrument MR screen of cortical proteins

Colocalization for proteins identified by cis-instrument MR screen. We used colocalization implemented in the *coloc* package[86] (using default priors) to assess whether the genes identified in the cis-instrument MR stage share one or more causal variants with the respective alcohol consumption behavior. We performed colocalization analysis testing for evidence of a single causal variant between the brain protein level and smoking behavior at the cis genomic locus of the respective encoding gene. We included all SNVs, i.e., no P-value or LD filtering, within the cis locus (that is, all SNVs within ±100 kb of the gene's genomic start- and end positions). We considered a posterior probability >0.8 as suggestive that the cortical proteins or cell-type genes

© 2024 Rosoff DB et al. *JAMA Psychiatry.*

and the respective alcohol consumption behavior share one or more causal variants in the gene region. We took the cortical proteins with cis-MR estimates (either IVW or Wald ratio depending upon the number of instrument SNVs) and also demonstrating evidence of a shared causal variant between the brain protein level and smoking behavior at the cis genomic locus of the encoding gene forward for further characterization (described below).

Replication in whole blood gene expression data. The cortical brain protein QTL data are derived from postmortem tissues from 3 major brain banks (ROS/MAP, Banner Health, and Mt. Sinai Brain Bank).[33] As outlined in several reviews and discussed in the **eDiscussion** (described below in the subsection with extended information on study limitations), postmortem brain tissue may be impacted by several sources of bias and other experimental factors that impact estimates derived from this type of data source.[87-89] Therefore, we aimed to replicate the cortical proteins from the initial cis-MR screen with QTL data derived from living donors. To our knowledge, there is currently no brain expression or protein QTL data derived from living donors.[90] Therefore, we used expression QTL derived from ~31 000 individuals in studies comprising the eQTLGen Consortium (described previously).[91] Using the same cis-instrumentation methods as for the initial cis-MR screen (SNVs within ±100 kb of the locus; P-values of association $< 5\times10^{-8}$; LD $R^2 < 0.1$), we were able to cis-instrument 18 of the 27 brain proteins and performed cis-MR using the same methods as described in the above **eMethods** subsection ("*MR statistical and sensitivity analyses*").

Phenome-wide MR to assess potential side-effect profiles of cortical proteins associated with smoking behavior. Due to the role that adverse side effects play in the failure of therapeutics during drug development,[92] we aimed to enhance our understanding of the therapeutic potential of the 27 cortical proteins associated with smoking behavior (surpassing correction for multiple comparisons in the cis-instrument MR screen and demonstrating evidence of colocalization, i.e., posterior probability of a shared causal variant >0.8). To achieve this, we conducted a phenome-wide MR study involving 368 diseases and biomarkers (**eTable 1**). Inclusion criteria comprised studies conducted in cohorts of European ancestry, sample sizes of at least 1 000 participants, with a minimum of 100 cases for binary variables, and availability of summary statistics (betas, standard errors, effect alleles) for 100 000 SNVs. Cis-instrument MR analysis, as outlined in the preceding sections, was used. We used a Bonferroni-corrected P-value threshold of 1.36×10^{-4} (0.05/368 outcomes) to determine associations of the with biomarkers or diseases, and compared the directions of the cis-instrument MR estimates with the indicated direction of the cis-instrument MR estimate that would be therapeutically indicated for reducing smoking behavior.

Transcriptomic imputation statistical methods

We were next interested in evaluating the transcriptomic underpinnings of the smoking-longevity relationships identified in the SVMR and MVMR analyses performed in the first part of the study. Because available eQTL data are from cross-sectional studies, like the Genotype-Tissue Expression (GTEx) Project and The Cancer Genome Atlas (TCGA), and does not reflect changes in gene expression over time, there are inherent challenges in determining the directionality of the observed associations for all transcriptomic imputation studies.

Because there are no longitudinal smoking behavior GWAS data available, to address the complexities arising from the temporal variability of both gene expression and smoking

© 2024 Rosoff DB et al. *JAMA Psychiatry.*

behavior, we employed a lifetime smoking behavior index as our main endpoint for tobacco smoking assessment.[93] It is a comprehensive measure designed to reflect an individual's smoking behavior over their life course, rather than solely focusing on aspects of current smoking behavior (e.g., current smoking status) (see ref.[93] for information on the construction of the lifetime smoking index GWAS).

We prepared each GWAS summary-level data file for transcriptomic imputation using the *munge_sumstats.py* script in LD Score Regression.[94] We performed transcriptomic imputation using the TWAS FUSION method following the FUSION protocol default settings on autosomal chromosomes.[95] The primary FUSION pipeline constitutes three steps: (1) identify gene expression features that are cis-heritable; (2) construct a linear predictor for each cis-heritable gene, i.e., a SNV-based prediction weight of the gene feature; and (3) calculate both TWAS test-statistics incorporating these SNV-based prediction weights and summary-level GWAS Z-scores.[96] FUSION uses several penalized linear regression and Bayesian sparse linear mixed models (e.g., GBLUP, LASSO, Elastic Net, BLSMM) and computes an out-of-sample R^2 statistics to identify the best model via a cross-validation of each model.[95] A cross-panel Bonferroni corrected P-value threshold of 8.31×10^{-7} [0.05/60 114 total tests across the panels]) was used to assess genes associated with lifetime smoking behavior.

For all genes surpassing Bonferroni correction for multiple comparisons, we assessed whether they were captured by the genetic signature of lifetime smoking (as represented by the lifetime smoking behavior genomic loci, i.e., the lead independent variants comprising the smoking instrument). We constructed 1 Megabase (Mb) windows around these lead variants (1 Mb upstream and 1 Mb downstream) and compared the TWAS-identified genes with these 2 Mb windows. If the TWAS-identified gene was located within these windows, then we considered it to be captured by the smoking GWAS signature, and if it was located > 1Mb from any smoking loci, then it was considered a novel association with smoking not captured by the input smoking GWAS.

Next, colocalization analysis statistics were generated for functional features whose TWAS P-value surpassed Bonferroni correction for multiple comparisons. PP.H4 measurements indicate the posterior probability that the functional feature and the GWAS share the same causal variant,[97] and were used to further screen the smoking-associated genes. Of the functional features eligible for colocalization analysis, only the features whose colocalization analysis against smoking exceeded a PP4 index of 0.6 were prioritized for further analysis.

Comparison of smoking-associated genes with aging phenotypes

As we aimed to provide gene-level resolution of the smoking-aging relationships identified in the polygenic SVMR and MVMR analyses, we took forward the colocalized smoking-associated genes and performed transcriptomic imputation of these features (gene-tissue pairs) in the multivariate longevity, GrimAge, and PhenoAge outcomes. We compared the FUSION Z scores (the primary association statistics of the FUSION method) between smoking and the aging-related outcomes for these genes and looked for Z scores that were consistent with the observed smoking SVMR and MVMR estimate, i.e., we looked for features that had opposing Z scores between smoking and multivariate longevity and concordant Z scores between smoking and EAA. In addition to comparing individual gene-tissue features, we also assess the tissue-level

transcriptome-wide correlations between smoking and the aging-related traits using the full TWAS summary statistics.

Bio-annotation of the smoking-associated genes

We performed bio-annotation of the high-confidence, colocalized genes associated with smoking to provide further biological characterization.

Gene ontology and pathway analysis: We used EnrichR[98] to perform gene ontology and pathway enrichment analyses using the high confidence TWAS genes associated with smoking behavior. We analyzed the smoking-associated genes for all tissues together and used the Gene Ontology (GO),[99] Reactome,[100] and KEGG[101] gene sets. 201 of the 241 genes were found in the gene sets and used for analysis. We report all gene sets with EnrichR enrichment P-values < 0.05.

Open Genes lookup of prioritized smoking-associated genes. The Open Genes database was developed to facilitate the cross-disciplinary integration of evidence supporting the involvement potential gene targets in aging-related processes.[102] The developers collected data for 2 402 genes with evidence of associations with aging and provided a comprehensive annotation of the genes relating to the strength of existing evidence for their involvement in lifespan-extending interventions, age-related changes, longevity associations, gene evolution, associations with diseases and hallmarks of aging, and functions of gene products.[102] We screened the genes using the Open Genes database scoring system regarding the strength of the evidence for their relationships with aging (i.e., high, moderate, low, lowest).[102]

Cell-type enrichment. We next aimed to identify cell-type enrichment of the colocalized smoking-associated genes. We took a tissue-agnostic approach and aimed to screen as many cell-types as possible for enrichment. Therefore, we used the WebCSEA package,[103] which has curated more than 5.5 million cells across 111 human tissue panels as well as 1 355 tissue-cell types from 61 human adult and fetal tissues comprising 12 organ systems.[103] 190 of the 202 unique (i.e., we removed duplicated genes across FUSION tissue weights) smoking-associated colocalized genes were available in WebCSEA for enrichment analysis and we used P-values to assess cell-type enrichment.

GTEx aging signatures for genes with associations with smoking and aging-related outcomes. For smoking-associated genes demonstrating FUSION Z that were directionally consistent with the MR analyses (e.g., increased smoking and decreased longevity/increased EAA), we aimed to see if there was evidence that these genes were differentially expressed with age. Therefore, we performed a differential expression analyses with these genes using the PrismEXP method (https://appyters.maayanlab.cloud/#/PrismEXP) (Prediction of gene Insights from Stratified Mammalian gene co-EXPression), implemented in its Python package[104] that is available as part of a suite of Appyters developed by the Ma'ayan Laboratory. PrismEXP uses the ARCHS4 gene expression resource[105] to calculate predicted gene functions from gene set data available as part of its catalog.[98,105,106] We investigated each gene individually using the GTEx transcriptomic aging signatures for the GTEx tissues from a recent study that looked for up- or down-regulated genes between the old and young donors in the GTEx data,[107] and defined statistical significance using the PrismEXP adjustment for P-values.

© 2024 Rosoff DB et al. *JAMA Psychiatry.*

eRESULTS

Cross-trait genetic correlation analyses suggest strong shared genetics across psychiatric disorders and substance use behaviors

We first performed cross-trait genetic correlation analysis to assess the genetic relationships among the psychiatric disorder and substance use behaviors used as exposures for the subsequent MR. Broadly, we found strong genetic correlations linking the psychiatric disorders and substance use behaviors. Full results are presented in **eTable 11** and **eFigure 3**. Bipolar disorder exhibited a substantial positive genetic correlation with schizophrenia ($r_g = 0.70$, standard error [SE] = 0.03, P-value = 8.87×10^{-147}), emphasizing shared genetic factors. Additionally, bipolar disorder demonstrated a modest positive correlation with smoking (r_g = 0.17, SE = 0.02, P-value = 1.89×10^{-14}), suggesting a potential genetic link. DPW and PAU exhibited a robust positive genetic correlation ($r_g = 0.78$, SE = 0.04, P-value = 4.89×10^{-87}), indicating shared genetic factors. DPW also showed a positive genetic correlation with smoking ($r_g = 0.34$, SE = 0.02, P-value = 6.59×10^{-63}). Moreover, major depression displayed positive genetic correlations with smoking ($r_g = 0.31$, SE = 0.03, P-value = 9.64×10^{-31}) and other psychiatric disorders. These results illustrate the complex genetic architecture underlying the connections between psychiatric disorders, alcohol use, and smoking, providing valuable insights into their shared genetic influences.

Transcriptomic imputation prioritizes gene-level mediators of the genetic liability for smoking behavior

Full results for the TWAS on lifetimes smoking behavior are presented in **eFigures 6-10, eTables 26-30**. 470 gene-tissue features (representing 249 unique genes) surpassed Bonferroni correction for multiple comparisons (**eTable 31**). Of these features, 46 were found to be novel (representing 37 genes), i.e., located more than 1 Mb from the genomic loci comprising the lifetime smoking genetic signature. Further, we found evidence of shared causal variants (posterior probability [PP.H4] > 0.8) between the respective genes and lifetime smoking behavior in 241 of the 470 gene-tissue features (150 of the 249 unique genes) (**eTable 32**). This indicates that the same genetic variants are driving the associations with the tissue-level genes expression and with smoking behaviors and suggests that these genes are transcriptome-levels mediators of the genetic liability for lifetime smoking behavior. Excitingly, 27 of the 37 novel genes were also among the high-confidence genes, including *SHC1* (SHC adaptor protein 1), *COQ5* (Coenzyme Q5 methyltransferase), *ARL17B* (ADP Ribosylation factor Like GTPase 17B), and *TOP2B* (DNA Topoisomerase 2 Beta). The high-confidence genes were implicated in a range of biological processes, including DNA repair, chromatin remodeling, and telomere assembly/maintenance (**eTable 33**). GO molecular function gene sets and Reactome pathways corroborated the involvement in chromatin and telomere functioning (e.g., Reactome: Chromatin Modifying Enzymes R-HSA-3247509 and Telomere Extension by Telomerase R-HSA-171319).

Our screen of more than 1 355 cell types in 12 human organ systems to assess the single cell landscape of the high-confidence genes found strong evidence of enrichment (defined here as enrichment P-values surpassing Bonferroni correction for all cells tested) in several white blood cell types (i.e., classical monocytes, bone marrow erythroid progenitors, and natural killer T-cells) and also cardiac muscle cells (**eFigures 11-12**, **eTable 34**). Notably, there was no

© 2024 Rosoff DB et al. *JAMA Psychiatry.*

evidence of enrichment for the smoking-associated genes among fetal organ systems, suggesting an age-dependent enrichment relationship. There was some evidence for enrichment in pulmonary microvascular endothelial cells (P-value=0.0018); however, it did not surpass correction for multiple testing.

Look-up of the high-confidence smoking-associated genes for involvement in aging processes. To further characterize gene targets that may link smoking with aging, we screened the colocalized, smoking-associated genes in the Open Genes database. 21 of the 150 colocalized genes were found in the Open Genes database curated gene list (2 402 total genes) and available for analysis. Three of the 21 genes demonstrated moderate or high levels of confidence for their involvement with aging processes: the novel smoking-associated gene *SHC1* ((high confidence); *XRCC6* (X-ray repair complementing defective repair in Chinese hamster cells 6) (moderate confidence); and *DGKZ* (diacylglycerol kinase zeta) (moderate confidence), supporting their potential link between smoking and aging processes (**eTable 35**).

Colocalized genes are shared between smoking and longevity

145 of the 150 high-confidence genes were available for comparison with our primary multivariate longevity outcome and the two epigenetic clocks that demonstrated relationships with smoking in the SVMR and MVMR analyses (GrimAge and PhenoAge) (**eTable 36**). 31 of these genes demonstrated TWAS P-values with either the longevity or EAA outcomes surpassing correction for the total number of colocalized smoking features (241 total features) with TWAS Z scores that were directionally consistent with the smoking-longevity MR estimates (e.g., if the gene demonstrated a positive relationship with smoking [TWAS Z score >0], then it also demonstrated an adverse relationship with longevity [TWAS Z scores <0]). For example, increased *PRMT6* expression in lung, whole blood, and squamous cell lung carcinoma with lower smoking behavior (TWAS Z scores for smoking = ~-5.5 for all tissues) and positively associated with longevity in these tissues (TWAS Z scores for longevity = ~4.95 for all tissues). These genes were taken forward to assess whether they demonstrate evidence of differential expression related to aging in the GTEx tissues.[107] Four of the 31 genes were not found in the GTEx aging signature data (*C14orf153*, *ATP5G1*, *ATP5J2*, and *FAM150B*). 25 of the 27 genes available for analysis demonstrated evidence for aging-related changes in expression (**eTable 37**). These genes were differentially expressed across several tissues, including whole blood, muscle, brain, heart, and adipose tissue, and several of the age-related differences in expression aligned with the TWAS direction of associations for smoking and longevity. For example, the TWAS analysis found that lower *PRMT6* (Protein Arginine N-Methyltransferase 6) was associated with increased smoking behavior and reduced longevity. Correspondingly, *PRMT6* expression was lower in older individuals in 3 tissues (muscle, blood, and stomach). Other genes had different aging-related expression patterns, e.g., increased *XRCC3* (X-ray repair complementing defective repair in Chinese hamster cells 3 – not to be confused with *XRCC6*, also a high-confidence smoking behavior gene) was associated with increased smoking behavior in lung tissue and reduced longevity and also was lower in older individuals in muscle, blood, and stomach tissues. There were also 46 high-confidence genes related to smoking behavior that also had directionally consistent relationships with longevity-related outcomes with TWAS P-values < 0.05 that did not surpass correction for multiple comparisons (e.g., *PSMA4*, *TOP2B*, and *ADD1*) (**Figure 4b** in the main manuscript, **eFigure 10**, **eTable 36**).

eDISCUSSION

Discussion of the lack of adverse schizophrenia and bipolar disorder associations with longevity

The lack of adverse genetics-based associations of SCZ and BD on longevity-related outcomes raises intriguing questions regarding the interplay between mental health disorders and overall well-being. One explanation for these null findings could be survival bias, wherein individuals who carry genetic predispositions for SCZ and BD but do not manifest the disorders may comprise a subset of resilient individuals. It is plausible that the genetic factors associated with SCZ and BD may exert complex effects on cognitive and emotional regulation, potentially fostering adaptive mechanisms that contribute to enhanced resilience and overall health. For example, previous work found that BD genes are associated with increased intelligence,[108] which may offset the shared genetics with smoking behavior (**eFigure 3, eTable 11**), resulting in an overall neutral association. Another explanation may be selection bias, wherein individuals with more severe SCZ and BD may be underrepresented in the GWAS source cohorts. Relatedly, it is possible that different symptoms within the spectrum of SCZ and BD may have varying impacts on aging and longevity, with certain aspects of these disorders more closely linked to negative health outcomes, and other aspects, less influential. Given the strong genetic correlation of SCZ and BD with smoking behavior, it is also possible that the strong genetic signature of smoking (i.e., more genetic variants than either SCZ or BD) captures part of the symptomology attributable to reduced longevity related to these disorders. Nevertheless, these findings emphasize future research is essential to unravel the intricate relationships between mental health, genetics, and longevity and inform the nuanced mechanisms that underlie these complex interactions.

Discussion of high-confidence genes associated with smoking behavior and longevity

We also extend the discussion for several of the prioritized smoking-associated genes from the TWAS and colocalization analyses.

First, *XRCC3* (X-ray repair complementing defective repair in Chinese hamster cells 3) is an important enzyme involved in the homologous recombination repair pathway, and plays a crucial role in maintaining genomic integrity.[109] As cells age, DNA damage accumulates, leading to increased susceptibility to diseases and impaired cellular function, and DNA repair genes like XRCC3 may have important roles in protecting individuals from aging and disease-causing agents, such as smoking.[110] Several human-based studies have linked *XRCC3* and other XRCC members (*XRCC4* and *XRCC6* – also one of the high-confidence genes associated with smoking in our TWAS and colocalization screen) with smoking and several cancer types[111-113] (e.g., lung cancer in the Han Chinese population[112]), including finding that *XRCC3* polymorphisms may act as modifiers of the effects smoking on pancreatic cancer[113] and bladder cancer.[111] More broadly, there is a DNA damage theory of aging[114] and investigating the therapeutic potential of *XRCC3* in the context of aging could unveil novel strategies to mitigate age-related genomic instability and promote healthier aging. By enhancing the efficiency of DNA repair through *XRCC3* modulation and other DNA repair agents, it may be possible to counteract the detrimental effects of accumulated DNA damage, potentially slowing down the aging process and reducing the risk of age-related diseases.[114]

© 2024 Rosoff DB et al. *JAMA Psychiatry.*

PRMT6 is an enzyme that belongs to the PRMT family and is involved in post-translational modification by catalyzing the methylation of arginine residues in proteins; previous work has shown that *PRMT6* promotes cell growth and prevents senescence, and its expression levels are elevated in cancer patients, suggesting it as a promising cancer drug target.[80] This role in epigenetic regulation and chromatin remodeling suggests potential implications in age-related changes in gene expression as epigenetic modifications, such as arginine methylation, contribute to the regulation of cellular functions and may influence the aging process.[115] In addition, PRMT6 was found to mediate inflammation (by the NF-κB/p65 pathway) caused by cigarette smoking in a mouse model of emphysema.[116] Exploring how *PRMT6* activity is modulated during aging and its specific targets in the context of age-related cellular changes could provide valuable insights into the molecular mechanisms underlying aging.

In addition to the high-confidence genes associated with smoking behaviors with corresponding estimates surpassing the stringent correction for multiple comparisons with the longevity-related outcomes, there were 46 high-confidence genes related to smoking behavior that also had directionally consistent relationships with longevity-related outcomes, with TWAS P-values < 0.05 that did not surpass the correction for multiple comparisons (adjusted P-value threshold = 0.000207). Among these potential targets, we will highlight *TOP2B* (DNA Topoisomerase 2 Beta) given its role in maintaining genomic stability,[117] which is considered a main causal factor for aging.[118] *TOP2B* is involved in DNA replication, repair, and recombination, crucial mechanisms for maintaining the integrity of the genome. As cells age, the cumulative effects of environmental exposures, such as cigarette smoke, can impact DNA integrity and repair mechanisms.[117,118] As cells age, the cumulative effects of environmental exposures, such as cigarette smoke, can impact DNA integrity and repair mechanisms.[117,118] *TOP2B* is widely-expressed, and considered an anti-cancer target,[117] and cells treated with TOP2B inhibitors die with chromosomal alterations.[119] The associations of *TOP2B* with smoking behavior and longevity that we found suggests a potential link between exposure to tobacco and alterations in genomic stability mediated by this enzyme.

We also highlight several of the novel smoking-associated genes. First, *SHC1*, which our TWAS and colocalization screen identified as a novel, high-confidence smoking-related gene, is an important target in aging-related research[120] based upon early mice studies finding that knockouts improved oxidative stress response and extended lifespans.[121] *SHC1* encodes a multifaceted signaling adaptor protein, which holds significant promise in both aging-related research.[122] In the context of aging, *SHC1* is known to play a crucial role in cellular pathways influencing proliferation, survival, and responses to stress.[122] More recently, it has been shown that increased *SHC1* and the dysregulation of the epigenetic signature within the SHC1 locus promotes lung cancer metastasis.[123,124] Further, a genome-wide DNA methylation study looking at the epigenetic impact of smoking identified SHC1 as one of the differentially methylated genes, providing a potential mechanistic link (and therapeutic target) between smoking and SHC1.[125] As cells age, dysregulation of these pathways can contribute to cellular senescence and the development of age-related diseases. By understanding how *SHC1* modulates these processes, researchers aim to uncover potential therapeutic strategies to mitigate age-associated cellular dysfunction and enhance stress resistance, ultimately promoting healthier aging.

© 2024 Rosoff DB et al. *JAMA Psychiatry.*

Finally, we discuss the novel smoking target, ARL17B, that also demonstrated directionally consistent TWAS associations with longevity and differential expression with age in the GTEx tissues. ARL17B is a member of the ADP-ribosylation factor-like (ARL) family, which plays a role in intracellular vesicle trafficking and cellular signaling pathways.[126] Intracellular vesicle trafficking plays an important role in various cellular functions, such as protein and lipid transport, organelle dynamics, and signal transduction, which are critical for maintaining cellular homeostasis[126] – a hallmark of aging.[127] Smoking impairs cellular homeostasis, potentially though inflammation and increased oxidative stress,[128] which suggests that ARL17B and other homeostatic response genes may be viable targets to reduce the impact of smoking.

Discussion of the potential pathways modeled by MVMR

Strengths of this study include the use of complementary SVMR and MVMR methods, each relying on related assumptions that assess the validity of the MR framework, providing confidence in the robustness of the results and strengthening causal inference.[129] Another strength is the use of MVMR, facilitating the simultaneous evaluation of both psychiatric disorders and substance use behaviors.[52,130]

Prior genetics-based analyses using SVMR to separately investigate the impact of substance use behaviors or psychiatric disorders on longevity outcomes[8] are limited in interpretation due to potential pleiotropy from related confounders,[52,130] i.e. vertical pleiotropy, present if the SNVs instrumenting their respective psychiatric disorder (or substance use behavior) impact longevity first via their impact on substance use behaviors, which would, in turn, impact longevity (**eFigure 17a**); or horizontal pleiotropy, present if SNV instruments for psychiatric disorders directly impact longevity by a corresponding role in substance use behaviors without mediation, violating also the third core assumption of MR (**eFigure 17b**).[31] Alternatively, psychiatric SNV instruments would potentially be subject to confounding bias if they impact the genetic propensity for psychiatric disorders and various substance use behaviors, but do not impact substance use behaviors on longevity (**eFigure 17c**). In these cases, MVMR more accurately models potential comorbidity between psychiatric disorders and substance use behaviors and provides unconfounded estimates of their impact on longevity, needed to guide comprehensive and targeted treatment programs aimed at treating the causal risk factors for reduced longevity in these populations.

Extended discussion of strengths & limitations

Strengths & limitations of the longevity-related outcomes. Here we expand upon the strengths and limitations of the longevity-relate outcomes discussed in the main manuscript. As this study aimed to assess the relationships of psychiatric disorders and aging related to chronic disease, and our multivariate aging outcome incorporates the genetics related to healthspan and lifespan, these results may capture a broad healthy aging liability that is important to inform potential prevention and intervention strategies aimed at improving healthy aging. However, despite the incorporation of data related to exceptional longevity, our results should not be interpreted through the lens of lifespan extension and instead with an emphasis of reduced burden of chronic illness. There are additional considerations important for study interpretation regarding the univariate input GWAS data comprising the multivariate aging outcome. For example, the univariate GWAS of healthspan incorporated into the multivariate aging outcome is dependent

© 2024 Rosoff DB et al. *JAMA Psychiatry.*

upon the sample composition and selection protocols in the UKB. Because the UKB is comprised of adults between 40-69 years, and is enriched in cardiovascular disease and cancer, but has relatively few cases of Alzheimer's disease,[66] there may be important genetic signals related to healthspan that are missed in this dataset. However, as cardiovascular disease is the leading cause of globally,[131] this cardiovascular disease enrichment in the UKB may have important implications for the generalizability of the healthspan outcome. Similarly, because risk factors and leading causes of death change over time,[132] the parental lifespan data may reflect the pattens of the causes of death in the United Kingdom from several decades ago, which may not fully approximate the current demographic characteristics among the UKB participants. Nevertheless, as healthy aging relates to the burden of chronic illness among psychiatric populations, addressing healthy aging in these populations and the healthspan-lifespan gap in these populations is particularly important.[133] For example, efforts to promote smoking cessation and improve mental health outcomes would complement interventions aimed at extending the period of healthy aging. This includes tailored approaches that address the unique challenges faced by individuals with psychiatric disorders, such as integrated care models that simultaneously target smoking cessation and mental well-being.

Outlined in the original GWAS by Zenin et al.,[5] the healthspan GWAS was constructed using Cox proportional hazards models for one of the 8 most common disease outcomes, including death, in the UKB. Zenin et al.,[5] found strong genetic relationships between the healthspan and lifespan GWAS data (rg=0.82), and were concerned about whether this relationship was driven by death events in the healthspan definition. Therefore, they performed a sensitivity analyses removing death from their healthspan definition and re-analyzed the relationship with lifepan, finding the relationships to be robust (rg=0.80), which suggests little impact of death being included in the definition of healthspan.[5]

More broadly, in study designs using Cox proportional hazards models, there is the potential for bias related to censoring by death, often termed competing events, where an participant may die before developing the disease or trait outcome of interest that they might otherwise have developed had they not died.[134] For example, studies of survival evaluating the risk for dementia or death by dementia may be susceptible to this type of bias as individuals who die by other chronic diseases (e.g., cardiovascular disease or cancer) would be unable to develop or die by dementia, which would bias the dementia-focused analyses.[134] While the broadly defined healthspan outcome may also be susceptible to this form of bias, because it is constructed of the 8 most common diseases in the UK Biobank, it may be more robust to competing event bias than a more narrow definition with only one or two age-related disease categories, or one that does not include death in its construction. For example, unlike a survival analysis focused solely on dementia that will misclassify participants if they die by cardiovascular disease or cancer prior to being diagnosed with dementia, these participants would, under the definition of healthspan in the GWAS constructed by Zenin et al., still be correctly identified as having a completed healthspan because these disease categories were included in its definition.

Nevertheless, despite the definition of healthspan encompassing the 8 most common diseases, there may still be some residual bias related to competing events. However, we also performed sensitivity analyses using the lifespan GWAS univariate GWAS data, one of the component longevity-related GWASs comprising the multivariate longevity outcome. These analyses were

© 2024 Rosoff DB et al. *JAMA Psychiatry.*

consistent with the main MR findings for the multivariate longevity outcome, suggesting robust estimates for the psychiatric disorders and substance use behaviors.

Regarding the epigenetic clocks, we emphasize that the epigenetic clocks from whole blood may not capture tissue-specific aging patterns. Different tissues in the body can age at different rates due to variations in cellular turnover, exposure to environmental factors, and the presence of tissue-specific epigenetic marks.[133] Whole blood epigenetic clocks provide a systemic overview of aging processes but may not accurately reflect the aging dynamics of specific tissues or organs.[8,9] Consequently, findings derived from whole blood epigenetic clocks may not fully represent the aging trajectories of particular tissues relevant to certain diseases or conditions, which highlights the need for follow up studies incorporating tissue-specific epigenetic data or developing specialized clocks for specific tissues when the data becomes available. Nevertheless, as they may reflect an overall system-level assessment of aging[8,9] and given the relative ease in obtaining blood samples from participants in clinical studies, they offer an exciting clinical endpoint to easily and accurately measure possible changes in biological age related to the interventions of interest (e.g., a hypothetical clinical trial evaluating the impact of smoking cessation on EAA among substance use disorder patients).

Finally, our findings do not directly investigate the potential for reduced longevity in psychiatric populations via suicide completions. Because of high mortality rates in psychiatric populations due to increased risk for chronic physical health conditions,[135,136] the aim of our study was to disentangle the impact of major psychiatric disorders and substance use behaviors on longevity outcomes related to chronic and age-related diseases and evaluate whether or not the genetic predisposition for psychiatric disorders is directly related to reduced aspects of longevity from chronic and age-related diseases. Risk for suicide ideation and completion is increased among those with psychiatric disorders relative to the general population.[137,138] Suicide accounts for ~800 000 annual worldwide deaths,[139] representing a cause of mortality in psychiatric populations not directly linked to chronic health and age-related diseases. Future studies are needed to further investigate the biological pathways linking psychiatric disorders and suicidality.

Extended discussion of genetic liability for time varying exposures including psychiatric disorders and substance use behaviors. As outlined in the **eMethods**, these genetics-based findings should be interpreted as the genetic liabilities for the psychiatric disorders and substance use behaviors and not the impact of the exposures themselves due to their time-varying nature.[140-143] For example, in the SVMR and MVMR models, we identified strong associations for the role of inherent genetic factors in predisposing individuals to smoking and its associations with longevity and epigenetic aging. These may be important to inform the preventive strategies targeting those with a higher genetic risk for smoking. The distinction between genetic liability and actual exposure interpretations is critical for both understanding the results and informing public health interventions. If the results suggest that genetic liability for smoking reduces longevity, it highlights the role of inherent genetic factors in predisposing individuals to smoking and its associated health risks. This knowledge can guide preventive strategies targeting those with a higher genetic risk in the context of this stable, lifelong genetic liability.

© 2024 Rosoff DB et al. *JAMA Psychiatry.*

By contrast, if the focus is on actual smoking exposure, interventions might be more directed towards smoking cessation and controlling environmental and social factors that influence smoking behavior, which may vary over time (e.g., patterns of smoking initiation, intensity, cessation, and relapse differing significantly among individuals). This variability would then profoundly affect the health outcomes associated with smoking, including longevity.[140]

For example, a population-based prospective study of ~68 000 Japanese adults found that among those who continued to smoke throughout the duration of the study, the overall mortality was more than doubled in both men and women (rate ratios versus never smokers: men 2.21, 95% confidence interval [CI], 1.97-2.48), women 2.61 (95% CI 1.98-3.44)) and that life expectancy was reduced by 8 years among men and 10 years among women,[144] suggesting a substantial reduction in mortality and morbidity for smoking cessation. However, the study also found that the benefit of smoking on mortality and morbidity was greater among those who stopped before age 35 (avoided all excess risk), which contrasted to those who stopped smoking with the next decade (before age 45): these individuals still reduced much of the excess risk, but slightly less than their counterparts who stopped earlier in life.[144] Similar results were observed by Jha et al. who analyzed ~200 000 adults in the United States National Health Interview Survey and found that an approximate 10 year reduction in life expectancy among the current smokers (versus never smokers), and also that smoking cessation led to life expectancy gains that differed some by age of smoking cessations: quitting smoking at ages 25 to 34, 35 to 44, or 45 to 54 resulted in 10, 9, and 6 years of life, respectively (versus adults who continued smoking).[145]

Importantly, as Jha et al. noted, these life expectancy gains from smoking cessation should not be interpreted as it being safe to smoke until ~25-40 years of age.[145] Among adults in the United States National Health Interview Surveys, they showed that there remains a substantial excess risk of (~20%), which is interpreted that among former smokers who die before the age of 80, one in six would have survived if they had experienced death rates similar to people of the same educational background, body fat, and alcohol usage who had never smoked.[145] This underscores the importance of considering both genetic predispositions and actual behavioral patterns in public health strategies aimed at reducing smoking-related mortality and morbidity.

Limitations related to homogeneity assumption. While the estimates for the psychiatric disorders and substance use behaviors were robust in both MVMR models accounting for socioeconomic factors and risk factors, and sensitivity analyses performed aimed at assessing the validity of the homogeneity assumption for the exposures suggested that the results are not biased by violations of the homogeneity assumption, we still cannot rule out that there may be violations of the homogeneity assumption (that the impact on the exposure resulting from changes in the level of the instrumental variable should be consistent in direction across all individuals),[19,39] that would bias the results; however, it is unclear in which direction the biases would impact the estimates.

Limitations related to selection bias. While MR estimates for the psychiatric disorder and substance use behaviors were consistent in MVMR models adjusting for potential predictors of selection (i.e., educational attainment), the psychiatric disorder estimates were robust across repeated analyses with the FinnGen cohort, and negative control analyses with self-reported tanning ability were null, altogether suggesting that results were not biased by potential predictors of selection, we are still unable to fully rule out that selection bias may be present,

which would impact these results. As discussed by Gkatsionis et al., selection bias may impact MR studies, but its potential impact may be less than that of other bias sources, including pleiotropy and population stratification.[146] Importantly, results from the negative control analyses used to assess population stratification[70] and also from the sensitivity analyses used to assess the plausibility of the core MR assumptions (i.e., complementary MR methods, screening for pleiotropic variants, MVMR, etc.) suggest minimal impact of these sources of biases in these analyses. However, along the lines of the limitation that the analyses were performed in participants of only European ancestry, we still emphasize that caution is warranted before generalization to other populations.

Retrospective case-control cohorts. Another potential limitation of the study is the inclusion of retrospective case-control cohorts as part of some of the GWAS meta-analyses used in this study. As discussed in the **eMethods**, it has been shown that MR using data from retrospective case-control studies may produce estimates subjected to ascertainment bias (where the retrospective cohort is ascertained upon disease status).[78] Fortunately, the case-control cohorts only comprised small percentages of the much larger meta-analyses, and we performed sensitivity analyses to assess potential biases that may be related to these cohorts being in the larger GWAS data, including leveraging exposure and outcome datasets that did not include retrospective case-control cohorts in their overall sample. Importantly, in this study, MR estimates derived using these meta-analyses were consistent with other analyses that did not have retrospective case-cohort studies among the included cohorts. For example, the multivariate longevity estimates were consistent with both the estimates from the epigenetic clocks and also sensitivity analyses using only the lifespan GWAS data from the UK Biobank.[1] Similarly, MR estimates for PAU aligned with the alcoholic drinks per week variable, which did not include a retrospective case-control cohort. We were also able to replicate the analyses of the main psychiatric disorders, including alcohol use disorder using clinical diagnoses from the FinnGen cohort,[27] a prospective population-based study. This replication was particularly important since the analyses using psychiatric disorder exposures comprised, in part, of retrospective case-control study data, which provides further support that the MR-based relationships were not artifacts of the case-control studies included in several of the large GWAS meta-analyses. Further, the GWASs themselves constructed using these case-control cohorts performed sensitivity tests as part of the GWAS analysis pipeline to assess the suitability of integrating these cohorts into the larger meta-analyses (e.g., comparing frequencies of each genetic variant are compared between cases and controls and testing to determine whether any specific SNVs are found more frequently in individuals with the disease compared to those without[147,148]). More broadly, we emphasize that all genomics-based studies are subject to forms of ascertainment bias (unless the entire genome of every individual in a population is sequenced) because common SNV-phenotype relationships are more likely to be captured in small samples than rare SNV-phenotype relationships.[148]

Potential bias due to additional two-sample MR assumptions. There may also be bias related to demographic differences for several of the exposure-outcome pairs (i.e., the MVP cohort and UK Biobank cohort), which may not fully meet the same sample assumption for two-sample MR studies.[72] Importantly, for the MVP data, which was used for the PAU exposure, Zhou et al. found strong genetic correlation between AUD diagnoses in the MVP cohort and AUDIT-P scores in the UK Biobank (the two traits and cohorts used for their PAU GWAS meta-analysis), and we found that the MVP PAU diagnoses has a strong genetic correlation with weekly alcohol

© 2024 Rosoff DB et al. *JAMA Psychiatry.*

consumption in the UK Biobank (**eFigure 3**), suggesting overlapping genetic architectures of these traits in these two cohorts. The MR estimates for PAU were in line with those from the weekly alcohol drinks consumed outcome derived from the UK Biobank. Additionally, replication analyses using diagnoses of alcohol use disorder (AUD) from electronic health records in the FinnGen cohort (latest release, N=412 181). Results using these FinnGen exposures aligned with the primary exposure datasets, suggesting robust relationships of AUD, schizophrenia, and bipolar disorder, which together suggests that any bias here may be minimal.

Comment about postmortem brain data. We would also like to acknowledge the potential limitations regarding the use of QTL data reflecting the genetic component of brain cortex proteins[33] used in this study to perform the cis-MR analyses to identify brain protein signatures of smoking behavior and potential therapeutic targets for future investigation.

Necessarily, these data are derived from postmortem tissues from 3 major brain banks (ROS/MAP, Banner Health, and Mt. Sinai Brain Bank).[33] As outlined in several reviews, postmortem brain tissue (versus, for example, biopsy tissue from living donors, which is only available under exceptional circumstances/conditions that require neurosurgical interventions[87]), which is an integral data source for neuropsychiatric research because the brain is the etiologically most important tissue,[87-89] may be impacted by several sources of bias and confounders. For example, there may be selection bias of the postmortem tissue[88] due to the non-random nature of the sample collection process. Individuals whose tissues are available for postmortem studies often have specific characteristics or conditions that led to their death and subsequent inclusion in the study. For instance, these individuals might have had certain neurological or psychiatric conditions, leading to an overrepresentation of these conditions in the sample. As a result, the genetic associations observed in postmortem QTL data may not accurately reflect the general population or other subpopulations.[87-89] We underscore that this is an important limitation of both the current cis-MR analyses presented in this study, and also the growing body of neuropsychiatric literature linking genomics with transcriptomic and proteomic levels in the brains, including several previously reported studies using these cortical protein data sources,[33,149-151] as well as the large body of literature leveraging the rich GTEx datasource.[152]

Another experimental factor important to consider postmortem brain tissue issue (and more generally any tissue type collected postmortem) is the series of complex changes the body undergoes at death, e.g., upon death the tissue will be subject to a wide range of biological changes, such as fluid shifts in both the intracellular and extracellular matrices, biomolecule degradation, intracellular vacuolization, apoptosis, and necrosis[88] (see Krassner et al. for a comprehensive review of cellular changes in brain-related tissue[88]).

Because there are currently brain QTL data sources not derived from postmortem samples, we attempted to replicate and validate our brain QTL findings using QTLs derived from living donors. We were able to successfully cis-instrument 18 of the 27 cortical proteins associated with smoking behavior from the initial screen. We found strong replication of the targets (10 of 18) in the whole blood, suggesting robust associations with smoking behavior. Notably, despite previous work finding that peripheral tissues are less suited for target discovery than brain tissue,[89] expression between whole blood and brain has been shown to be correlated,[153,154] and in the context as replication analyses/sensitivity tests using samples from living donors, these

© 2024 Rosoff DB et al. *JAMA Psychiatry.*

findings, when combined with the initial screen of brain proteins in relevant tissue for the predisposition for smoking behavior,[155-157] provide important support for these targets. Nevertheless, we underscore the importance of interpreting the cis-instrument MR analyses and findings through the lens of the experimental factors present in the underlying data and cohort composition.

Comment regarding diversity in genetics-based research

As discussed in the main manuscript, this study uses genetics-based data from participants of European ancestry, and therefore, we emphasize caution in generalizing the findings to non-European populations and also the need for replication of these findings in non-European cohorts when outcomes related to longevity (e.g., lifespan, healthspan, and exceptional longevity) become available. More generally, there exists a need to improve race/ancestry representation in genetics-based studies across all clinical disciplines.[158-164] As outlined by Fatumo et al.,[161] the imperative for increased genetic diversity in genomic studies is underscored by the prevailing imbalance, where the majority of data comes from individuals of European ancestry, leaving other populations underrepresented.[161] This European-centric bias not only raises ethical concerns but also results in missed scientific opportunities and health disparities.[161] For example, inadequate representation impedes the identification of population-specific variants and, in the application of Mendelian randomization studies, potential ancestry-specific differences in the causal roles of important risk factors and biomarkers in disease risk. It also limits the accuracy of polygenic risk scores for diverse populations, and overlooks clinically important variants discovered exclusively in underrepresented groups.[161] Addressing the inequalities in genomic studies requires a concerted global effort to implement a roadmap for increased diversity.[161] These initiatives should leverage existing research infrastructure, capacity, expertise, and leadership within local institutions. Further, overcoming historical injustices, building trust, and considering ethical, legal, and social implications in study design are essential for engaging diverse populations in genomic research.[158-164] Ultimately, fostering genetic diversity is not only an ethical imperative but also crucial for advancing scientific understanding, reducing health disparities, and ensuring the applicability of genetic insights across a broad spectrum of populations.

eCHECKLIST: STROBE-MR Reporting Guidelines.

1. TITLE and ABSTRACT
Indicate Mendelian randomization as the study's design in the title and/or the abstract.
Title and abstract

INTRODUCTION
2. Background
Explain the scientific background and rationale for the reported study. Is causality between exposure and outcome plausible? Justify why MR is a helpful method to address the study question.
Addressed in the Introduction and Methods

3. Objectives

© 2024 Rosoff DB et al. *JAMA Psychiatry.*

State specific objectives clearly, including pre-specified causal hypotheses (if any).
Addressed in the Introduction.

METHODS
4. Study design and data sources
Present key elements of study design early in the paper. Consider including a table listing sources of data for all phases of the study. For each data source contributing to the analysis, describe the following:
a) Describe the study design and the underlying population from which it was drawn. Describe also the setting, locations, and relevant dates, including periods of recruitment, exposure, follow-up, and data collection, if available.
b) Give the eligibility criteria, and the sources and methods of selection of participants.
c) Explain how the analyzed sample size was arrived at.
d) Describe measurement, quality and selection of genetic variants.
e) For each exposure, outcome and other relevant variables, describe methods of assessment and, in the case of diseases, the diagnostic criteria used.
f) Provide details of ethics committee approval and participant informed consent, if relevant.
Addressed in the Methods and eMethods.

5. Assumptions
Explicitly state assumptions for the main analysis (e.g. relevance, exclusion, independence, homogeneity) as well assumptions for any additional or sensitivity analysis.
Addressed in the eMethods.

6. Statistical methods: main analysis
Describe statistical methods and statistics used.
a) Describe how quantitative variables were handled in the analyses (i.e., scale, units, model).
b) Describe the process for identifying genetic variants and weights to be included in the analyses (i.e, independence and model). Consider a flow diagram.
c) Describe the MR estimator, e.g. two-stage least squares, Wald ratio, and related statistics. Detail the included covariates and, in case of two-sample MR, whether the same covariate set was used for adjustment in the two samples.
d) Explain how missing data were addressed.
e) If applicable, say how multiple testing was dealt with.
Addressed in the Methods and eMethods.

7. Assessment of assumptions
Describe any methods used to assess the assumptions or justify their validity.
Addressed in the eMethods.

8. Sensitivity analyses
Describe any sensitivity analyses or additional analyses performed.
Addressed in the Methods and eMethods.

9. Software and pre-registration
a) Name statistical software and package(s), including version and settings used.

© 2024 Rosoff DB et al. *JAMA Psychiatry.*

Addressed in the Methods.

b) State whether the study protocol and details were pre-registered (as well as when and where).
Addressed in the eMethods.

RESULTS
10. Descriptive data
a) Report the numbers of individuals at each stage of included studies and reasons for exclusion. Consider use of a flow-diagram.
b) Report summary statistics for phenotypic exposure(s), outcome(s) and other relevant variables (e.g. means, standard deviations, proportions).
c) If the data sources include meta-analyses of previous studies, provide the number of studies, their reported ancestry, if available, and assessments of heterogeneity across these studies. Consider using a supplementary table for each data source.
d) For two-sample Mendelian randomization:
i. Provide information on the similarity of the genetic variant-exposure associations between the exposure and outcome samples.
ii. Provide information on extent of sample overlap between the exposure and outcome data sources.
Addressed in the Methods, Results and eTables.

11. Main results
a) Report the associations between genetic variant and exposure, and between genetic variant and outcome, preferably on an interpretable scale (e.g. comparing 25th and 75th percentile of allele count or genetic risk score, if individual-level data available).
b) Report causal effect estimate between exposure and outcome, and the measures of uncertainty from the MR analysis. Use an intuitive scale, such as odds ratio, or relative risk, per standard deviation difference.
c) If relevant, consider translating estimates of relative risk into absolute risk for a meaningful time-period.
d) Consider any plots to visualize results (e.g. forest plot, scatterplot of associations between genetic variants and outcome versus between genetic variants and exposure).
Addressed in the Results and eTables.

12. Assessment of assumptions
a) Assess the validity of the assumptions.
b) Report any additional statistics (e.g., assessments of heterogeneity, such as I2, Q statistic).
Addressed in the Results, eTables and Discussion.

13. Sensitivity and additional analyses
a) *Use sensitivity analyses to assess the robustness of the main results to violations of the assumptions.*
b) Report results from other sensitivity analyses (e.g., replication study with different dataset, analyses of subgroups, validation of instrument(s), simulations, etc.).
c) Report any assessment of direction of causality (e.g., bidirectional MR).

© 2024 Rosoff DB et al. *JAMA Psychiatry.*

d) When relevant, report and compare with estimates from non-MR analyses.
e) Consider any additional plots to visualize results (e.g., leave-one-out analyses).
Addressed in the Results and eTables.

DISCUSSION
14. Key results
Summarize key results with reference to study objectives.
Addressed in the Discussion.

15. Limitations
Discuss limitations of the study, taking into account the validity of the MR assumptions, other sources of potential bias, and imprecision. Discuss both direction and magnitude of any potential bias, and any efforts to address them.
Addressed in the Discussion.

16. Interpretation
a) Give a cautious overall interpretation of results considering objectives and limitations. Compare with results from other relevant studies.
b) Discuss underlying biological mechanisms that could be modelled by using the genetic variants to assess the relationship between the exposure and the outcome.
c) Discuss whether the results have clinical or policy relevance, and whether interventions could have the same size effect.
Addressed in the Discussion.

17. Generalizability
Discuss the generalizability of the study results (a) to other populations (i.e. external validity), (b) across other exposure periods/timings, and (c) across other levels of exposure.
Addressed in the Discussion and eDiscussion.

OTHER INFORMATION
18. Funding
Give the source of funding and the role of the funders for the present study and, if applicable, for the original study or studies on which the present article is based.
Addressed in the Funding.

19. Data and data sharing
Present data used to perform all analyses or report where and how the data can be accessed. State whether statistical code is publicly accessible and if so, where.
Addressed in the Methods.

20. Conflicts of Interest
All authors should declare all potential conflicts of interest.
Addressed in the Conflicts of interest.

© 2024 Rosoff DB et al. *JAMA Psychiatry.*

eFIGURES

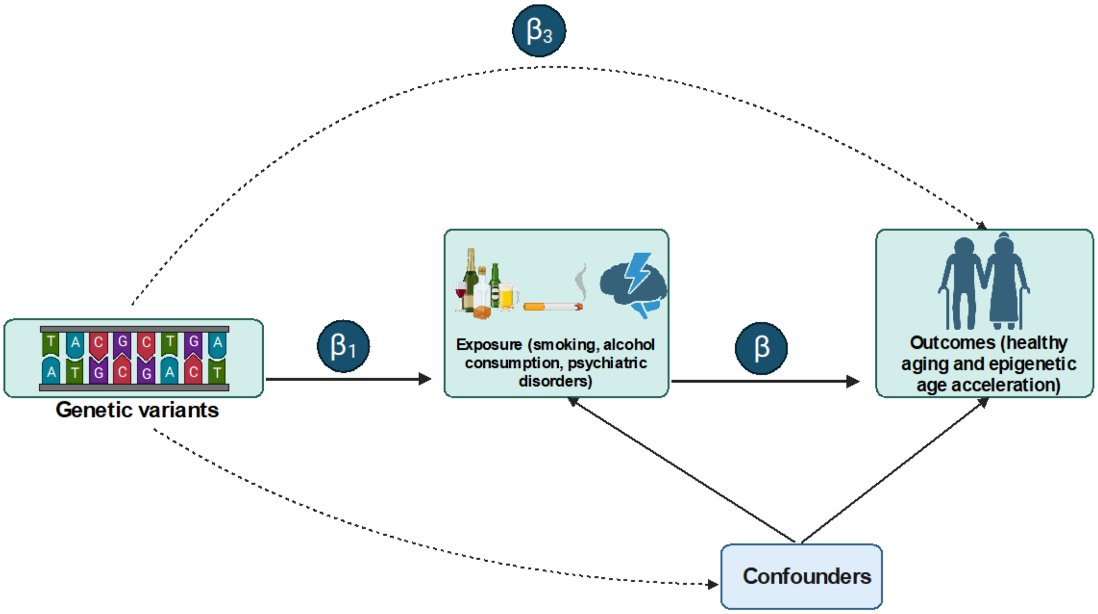

eFigure 1. Mendelian Randomization Model and Assumptions. β_2 is the genetic association of interest, estimated by $\beta_2 = \beta_1 / \beta_3$. β_1 and β_3 are the associations of the genetic variants with the exposure and the outcome. MR assumes that the genetic variants comprising the instrument for the exposure only impact the outcome of interest via the exposure and not directly, or via confounders (dotted lines).[19]

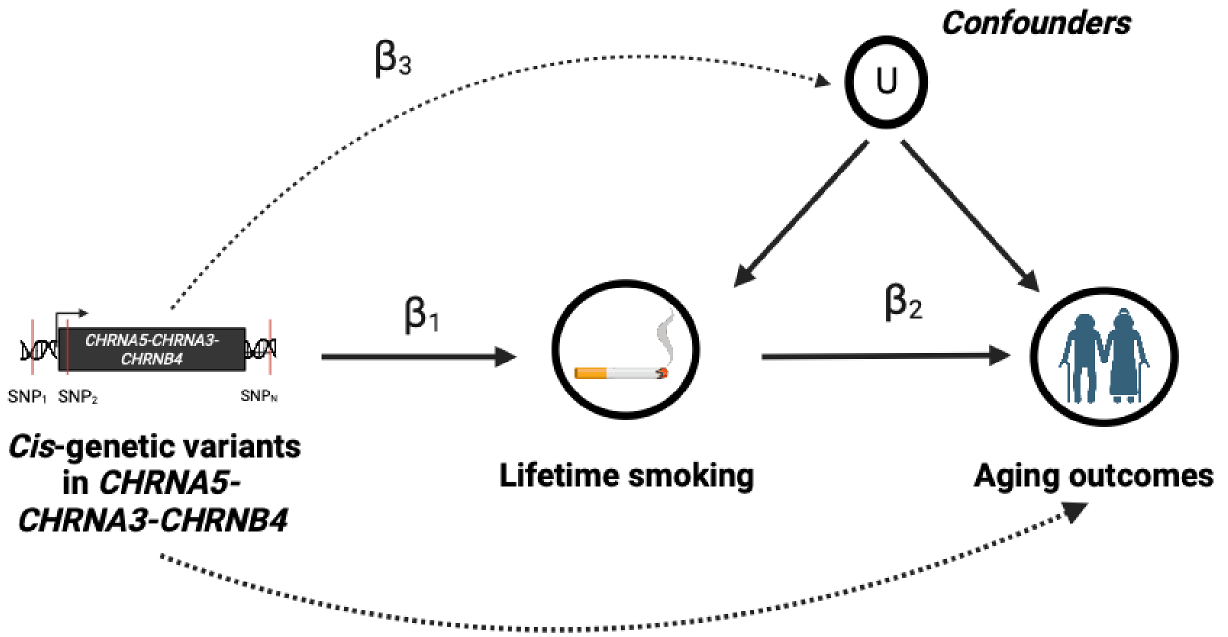

eFigure 2. Mendelian Randomization (MR) Model and Assumptions applied to *CHRNA5-CHRNA3-CHRNB4* gene cluster. As with conventional polygenic MR, β_2 is the genetic association of interest, estimated by $\beta_2 = \beta_1 / \beta_3$. β_1 and β_3 are the associations of the genetic variants with the exposure and the outcome. In this cis-instrument MR application, we constructed a genetic instrument for lifetime smoking using variants only located within or near (within 100 kilobases) of the *CHRNA5-CHRNA3-CHRNB4* gene cluster which encodes nicotinic acetylcholine receptors. We then performed cis-instrument MR analysis assessing the impact of smoking on aging outcomes (healthy aging and epigenetic aging). Because cis-instrument MR variants are located within the locus of genes with well-known effects on the exposure of interest (here mediating the effects of nicotine), they are less prone to pleiotropy and provide important addition support of the polygenic MR analyses.

© 2024 Rosoff DB et al. *JAMA Psychiatry.*

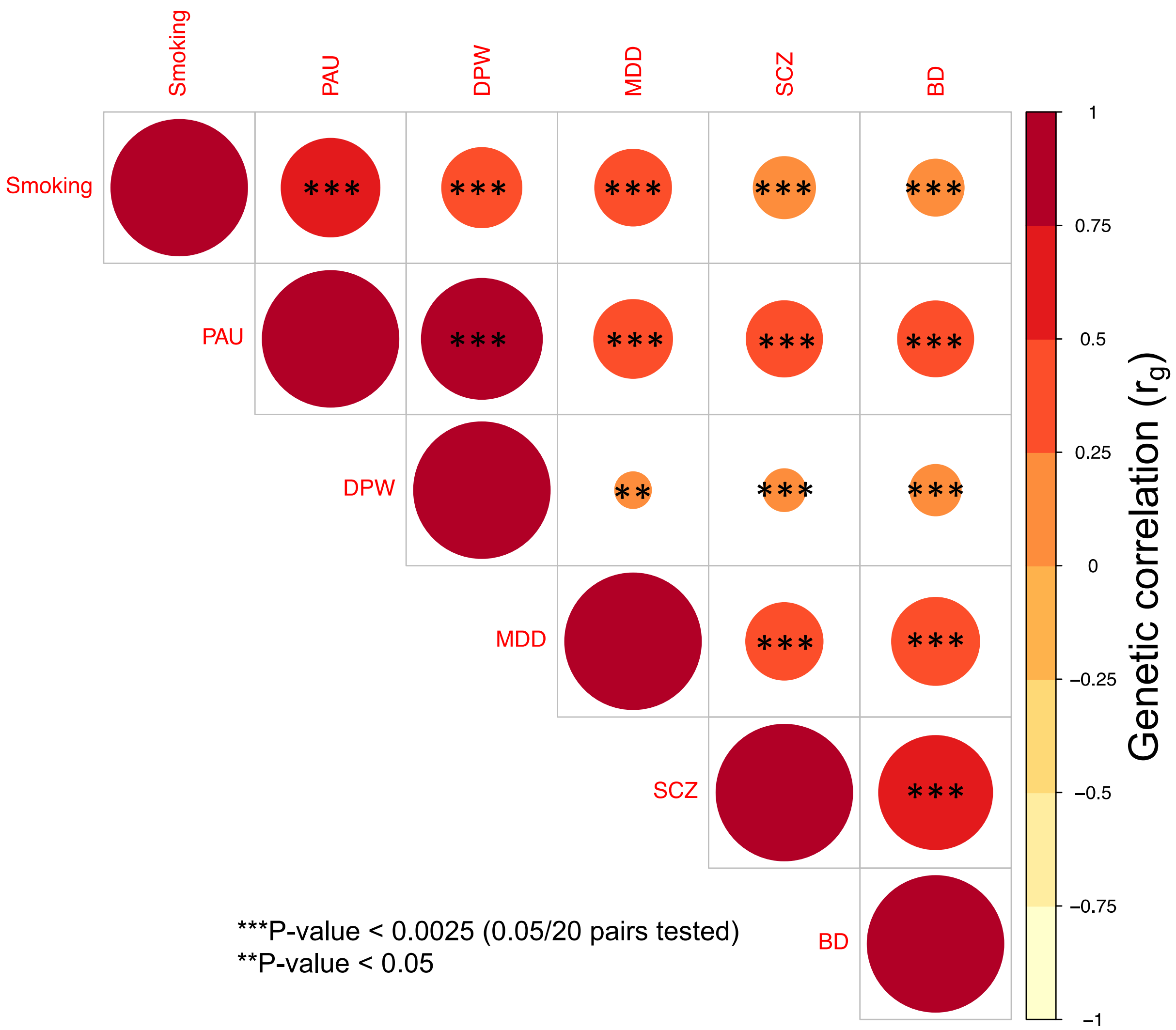

eFigure 3. Cross-trait genetic correlations between the psychiatric disorders and substance use behaviors used as Mendelian randomization exposures. All genetic correlations were positive (e.g., increased SCZ was genetically correlated to increased smoking, etc.).

Abbreviations: SCZ: schizophrenia, BD: bipolar disorder: MDD: major depression; PAU: problematic alcohol use; DPW: drinks per week.

© 2024 Rosoff DB et al. *JAMA Psychiatry.*

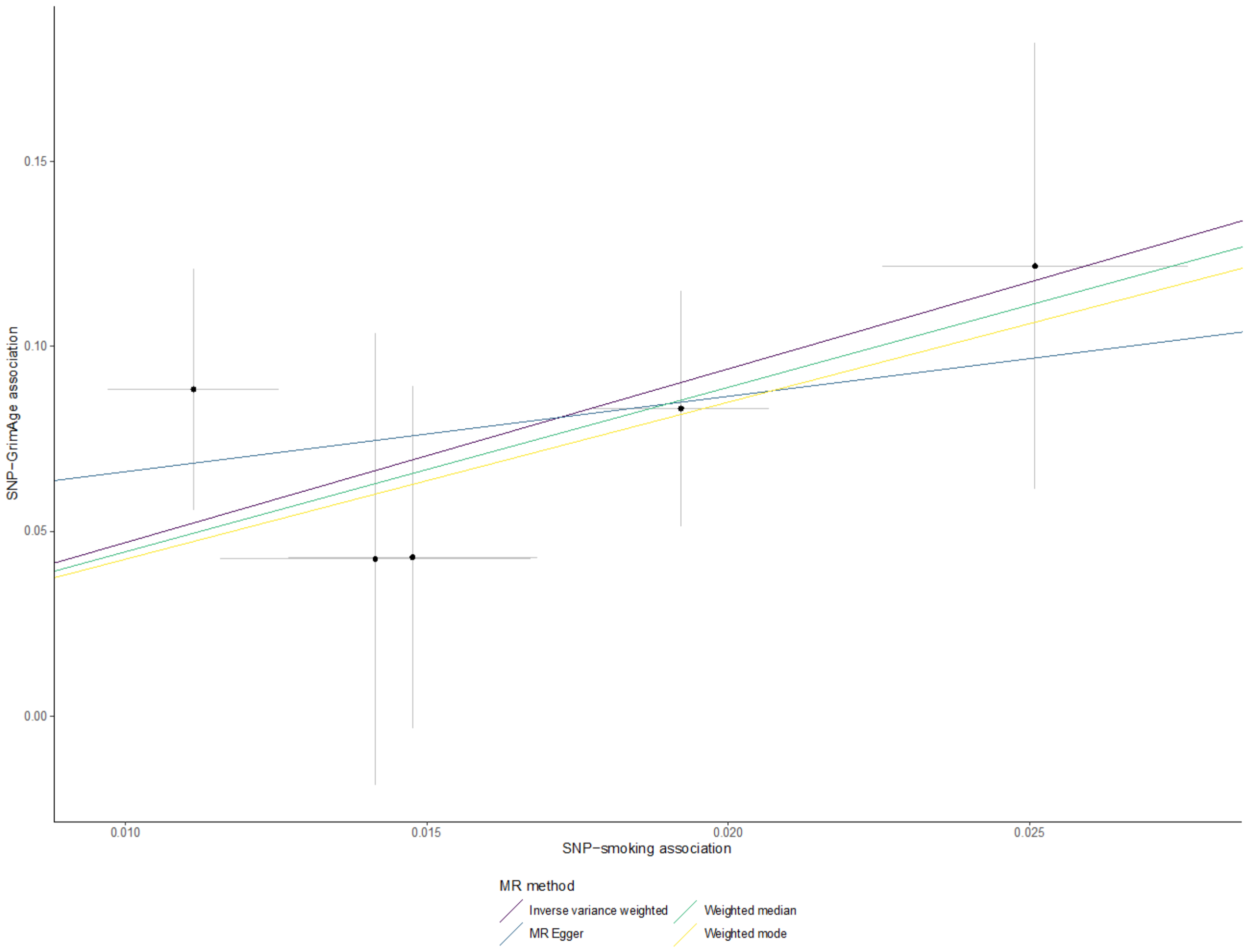

eFigure 4. SNV-SNV plot for Mendelian Randomization (MR) results assessing the relationship of smoking on *GrimAge* using the smoking instrument comprised of variants in the *CHRNA5-CHRNA3-CHRNB4* gene cluster. Points plotted are the associations statistics for the 5 variants comprising the *CHRNA5-CHRNA3-CHRNB4* gene cluster smoking instrument (x-axis are the SNV-smoking association statistics; y-axis are the corresponding SNV-aging association statistics form the multivariate longevity data). The regression lines correspond to the main inverse variance weighted and complementary MR methods.

© 2024 Rosoff DB et al. *JAMA Psychiatry.*

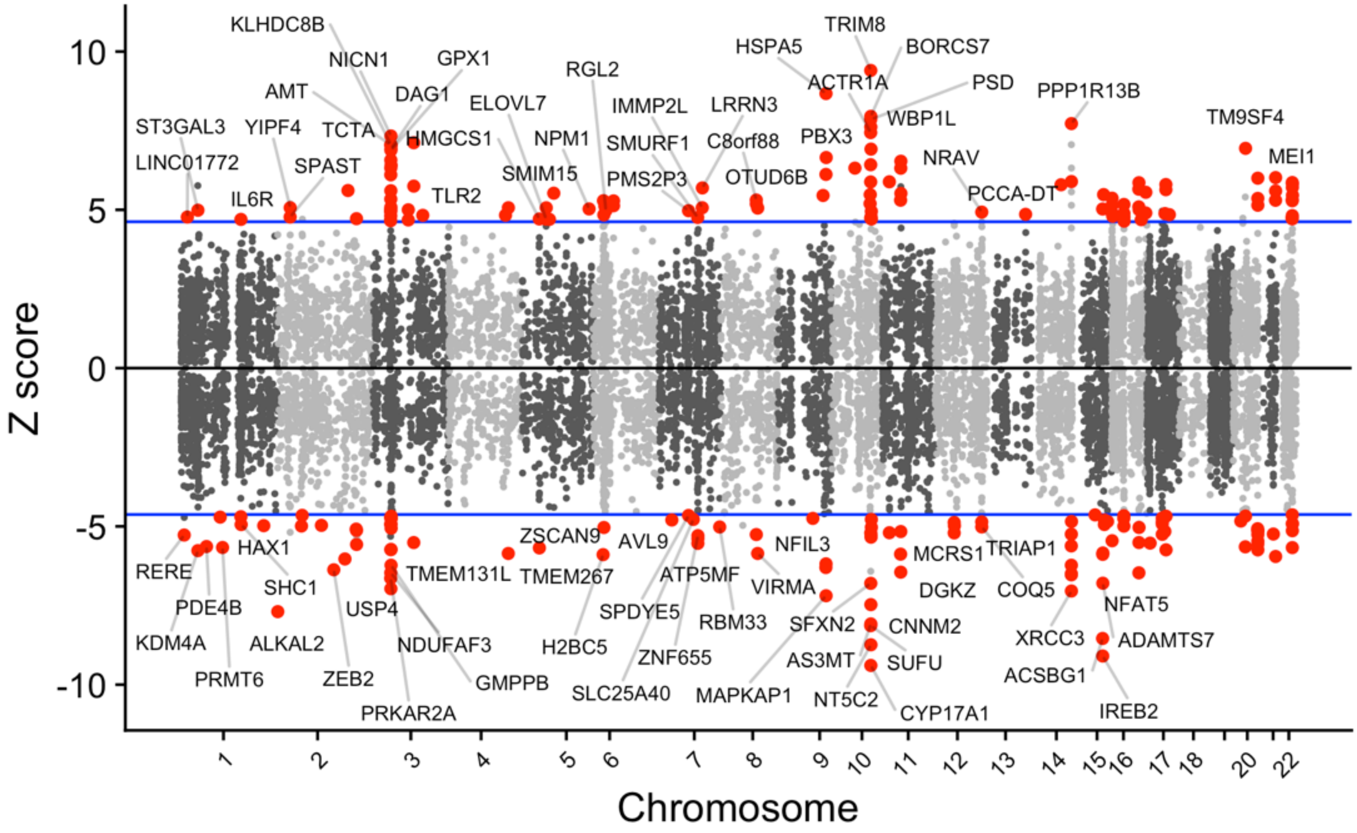

eFigure 5. FUSION transcriptome-wide association study (TWAS) of lifetime smoking using the sparse canonical correlation GTEx weights. Plotted are TWAS Z-scores. Highlighted genes surpass correction for multiple comparisons. Highlighted genes surpass correction for multiple comparisons for genes analyzed in the individual tissue (37 917 genes analyzed in the sparse canonical correlation GTEx weights).

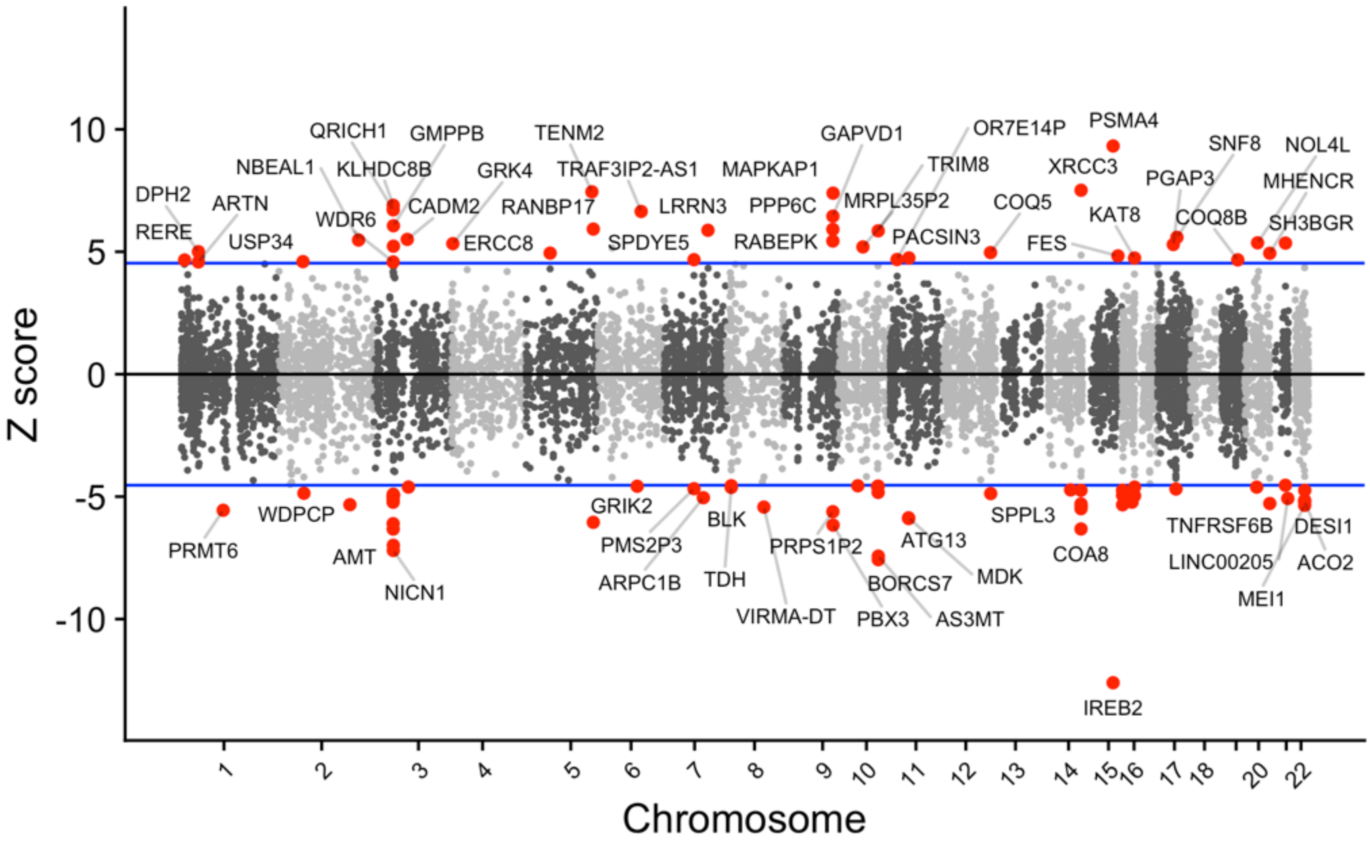

eFigure 6. FUSION transcriptome-wide association study (TWAS) of lifetime smoking using weights from GTEx lung tissue. TWAS Z-scores are plotted. Highlighted genes surpass correction for multiple comparisons for genes analyzed in the individual tissue (8,573 genes analyzed in the GTEx lung tissue).

© 2024 Rosoff DB et al. *JAMA Psychiatry.*

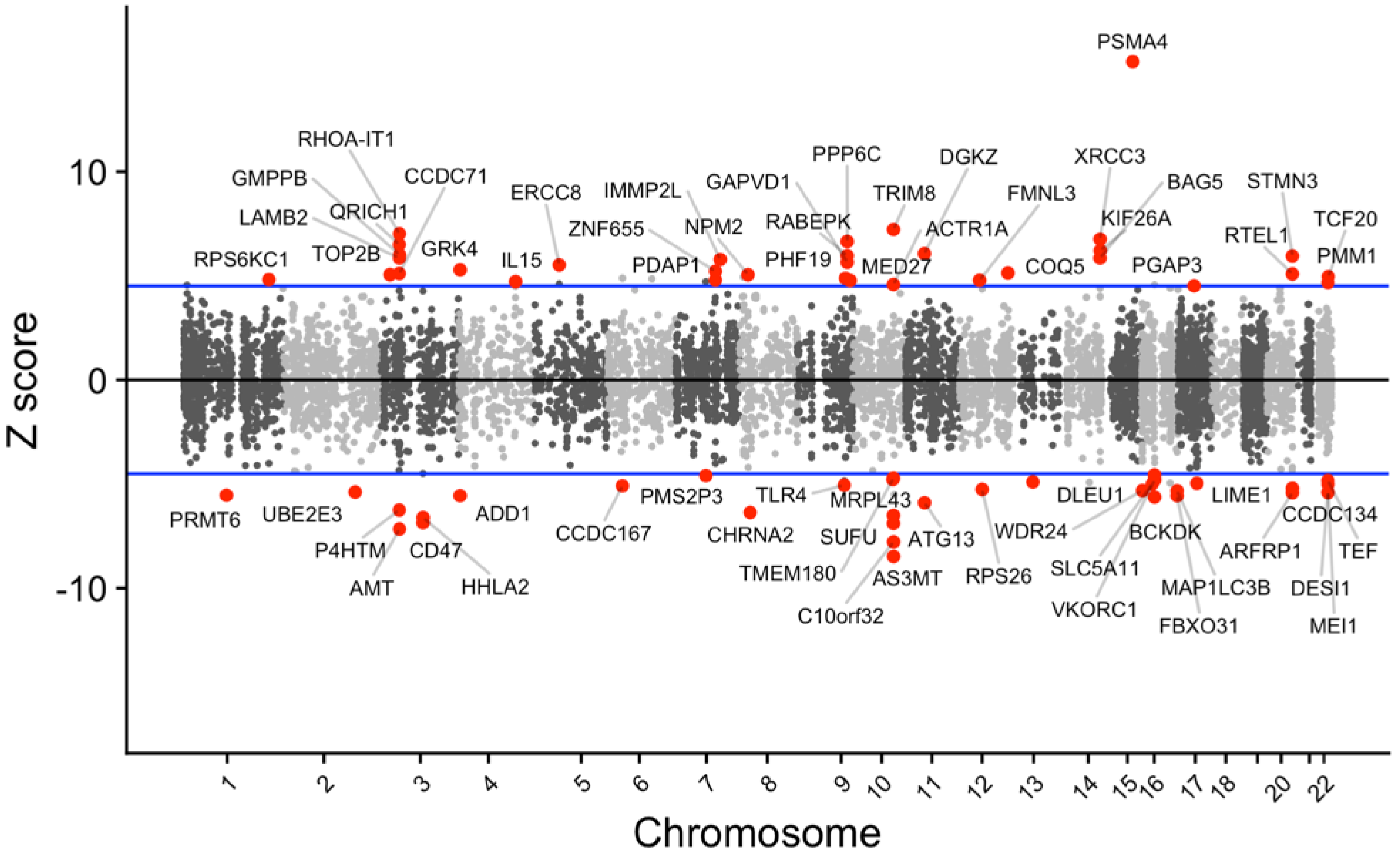

eFigure 7. FUSION transcriptome-wide association study (TWAS) of lifetime smoking using whole blood gene expression (from GTEx). TWAS Z-scores are plotted. Highlighted genes surpass correction for multiple comparisons for genes analyzed in the individual tissue (7 981 genes analyzed in the GTEx whole blood).

© 2024 Rosoff DB et al. *JAMA Psychiatry.*

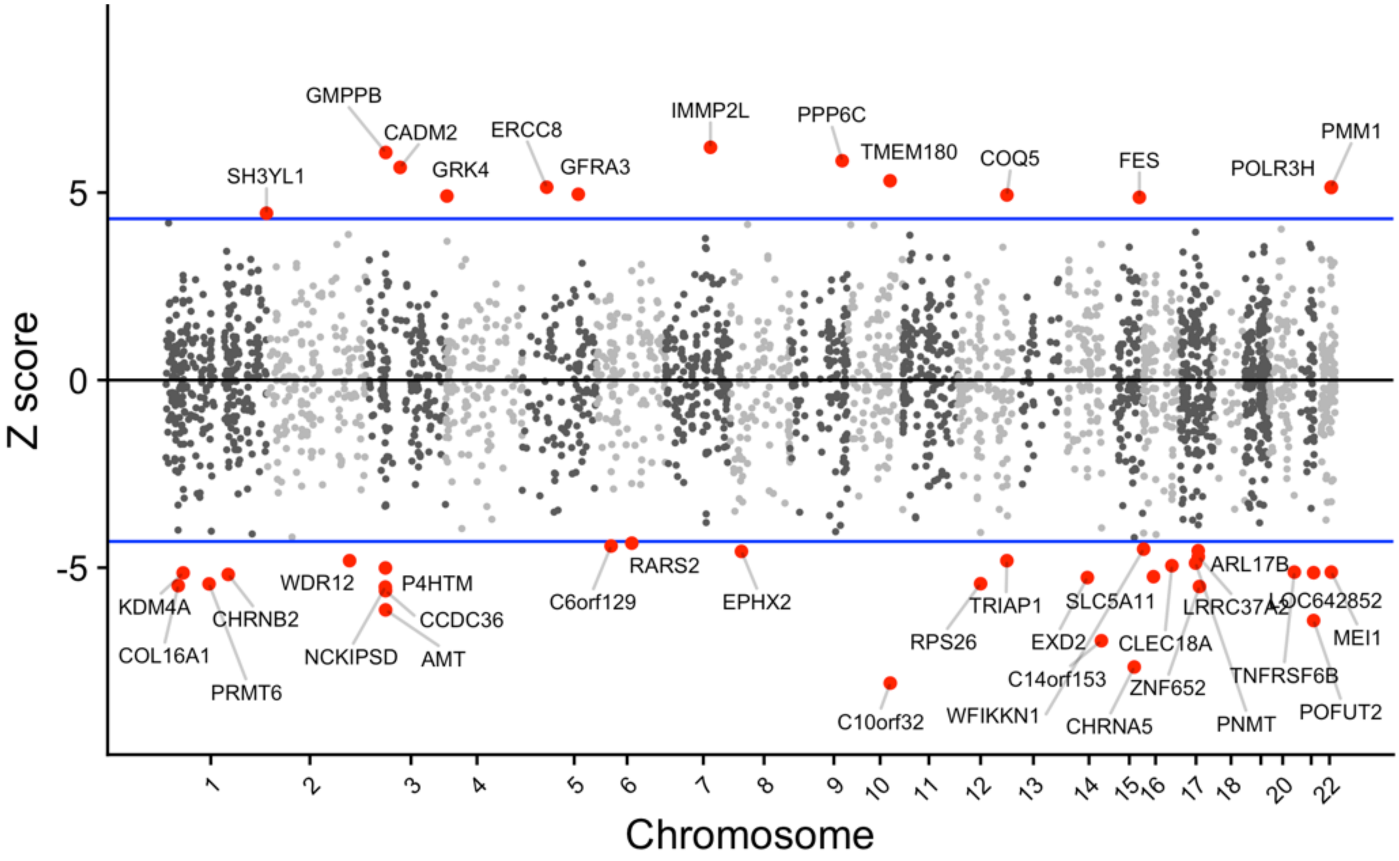

eFigure 8. FUSION transcriptome-wide association study (TWAS) of lifetime smoking using The Cancer Genome Atlas (TCGA) lung adenocarcinoma tissue. TWAS Z-scores are plotted. Highlighted genes surpass correction for multiple comparisons for genes analyzed in the individual tissue (2 948 genes analyzed in the TGCA lung adenocarcinoma tissue).

© 2024 Rosoff DB et al. *JAMA Psychiatry.*

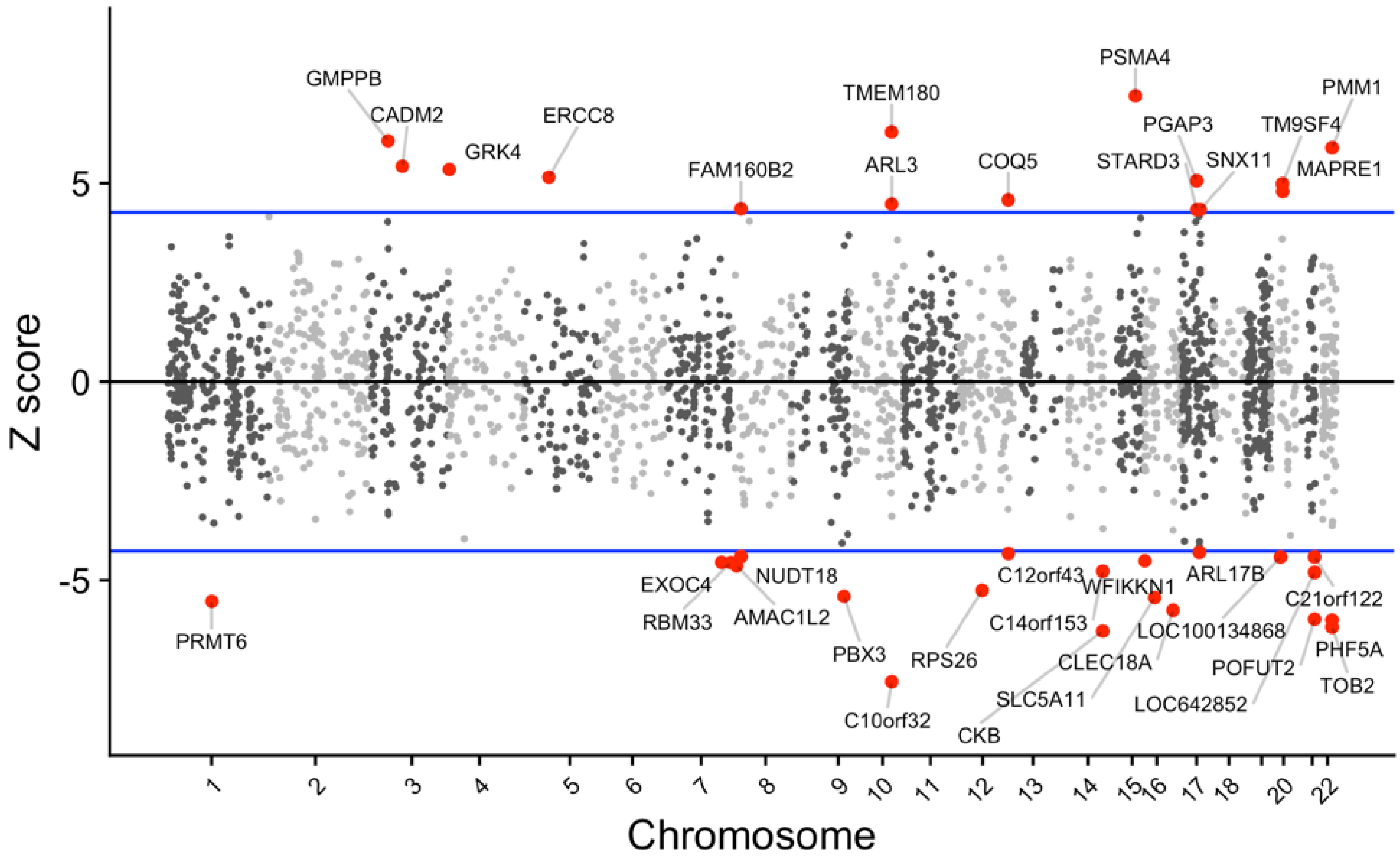

eFigure 9. FUSION transcriptome-wide association study (TWAS) of lifetime smoking using The Cancer Genome Atlas (TCGA) lung squamous cell carcinoma tissue. TWAS Z-scores are plotted. Highlighted genes surpass correction for multiple comparisons for genes analyzed in the individual tissue (2 515 genes analyzed in the TGCA lung squamous cell carcinoma tissue).

© 2024 Rosoff DB et al. *JAMA Psychiatry.*

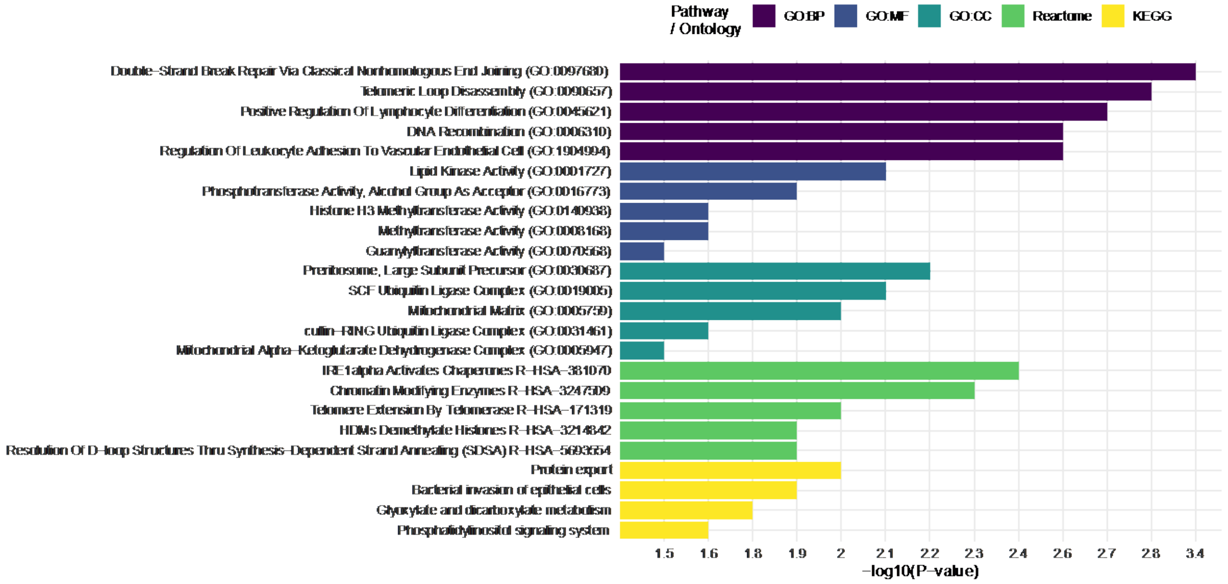

eFigure 10. Gene set enrichment results for smoking-associated genes. Presented are bar plots (-log10[P-values] for enrichment) of the Gene Ontology (GO) and pathway enrichment results for the top five ontologies or pathways of the high-confidence genes associated with smoking behavior. The KEGG pathway only had four gene-sets with enrichment P < 0.05, and therefore, only four KEGG pathways were plotted. They x-axis is the -log10(P-value) for the enrichment test.

Abbreviations: BP: biological processes; CC: cellular component; MF: molecular function

© 2024 Rosoff DB et al. *JAMA Psychiatry.*

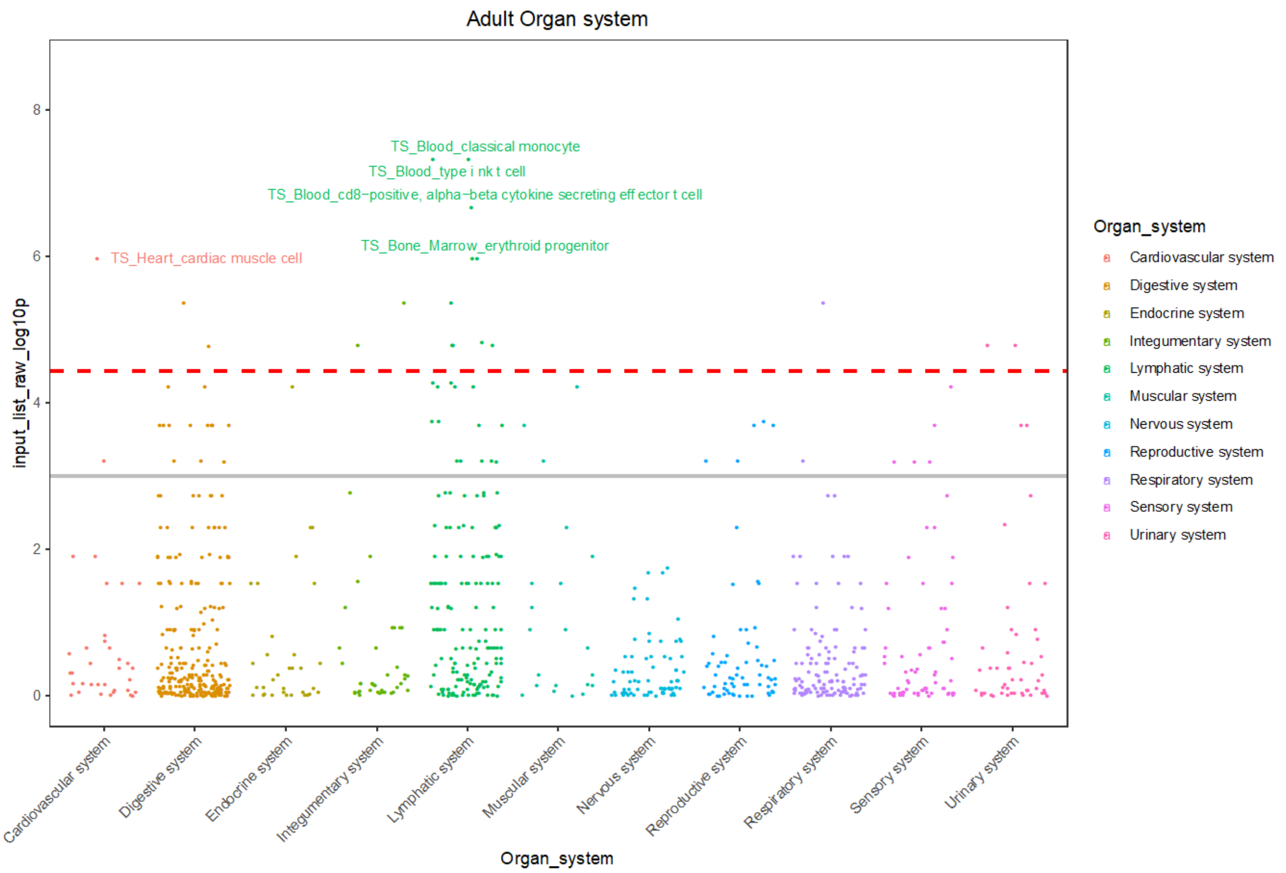

eFigure 11. Adult organ system cell-type enrichment of the high-confidence genes associated with smoking behavior. Manhattan plot presents the -log10(P-values) for the enrichment of the gene-level signatures for smoking behavior (TWAS genes that surpassed correction for multiple comparisons and also demonstrated evidence of a shared causal variant in the gene locus). Labeled cell types surpass Bonferroni correction for multiple comparisons.

© 2024 Rosoff DB et al. *JAMA Psychiatry.*

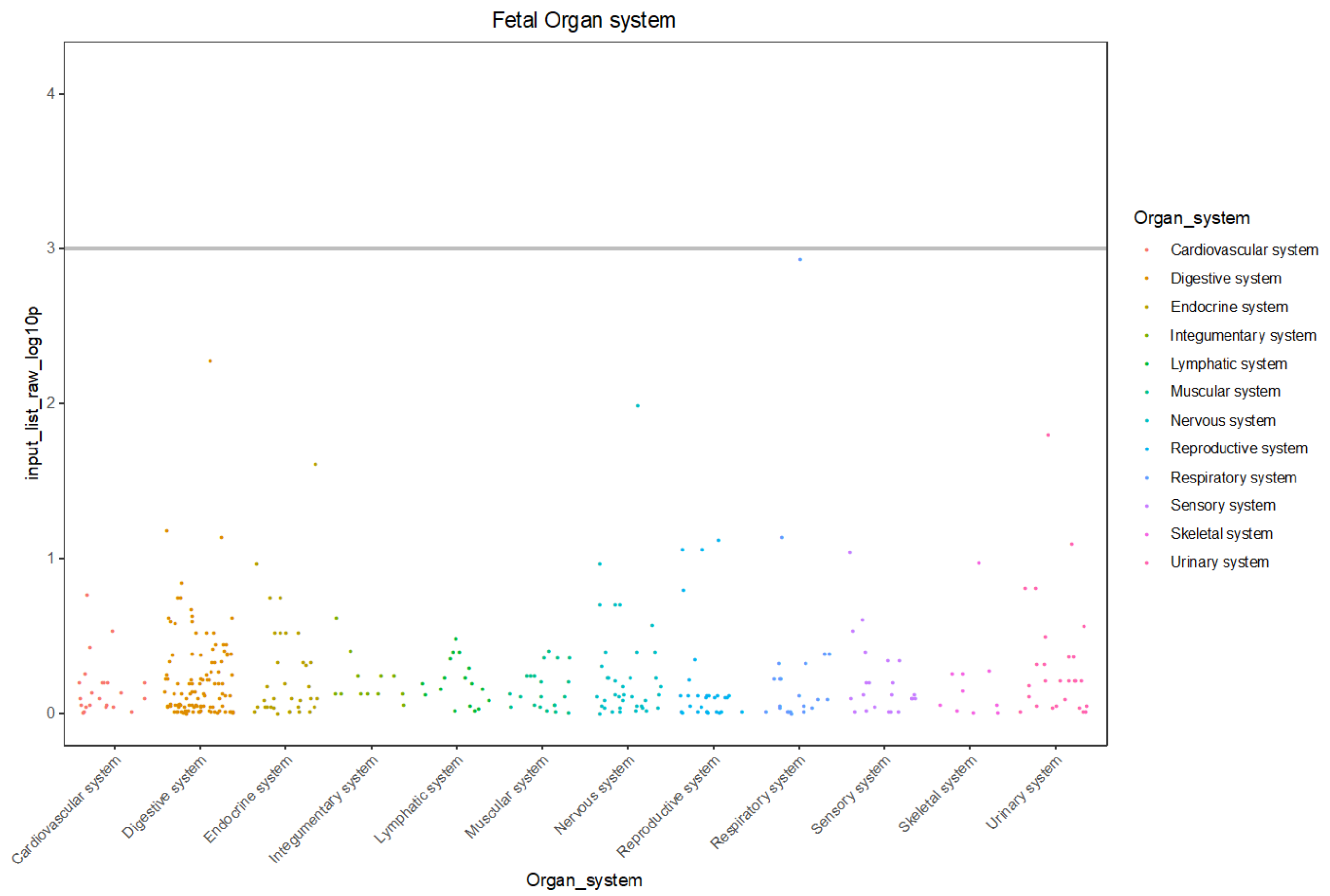

eFigure 12. Fetal organ system cell-type enrichment of the high-confidence genes associated with smoking behavior. Manhattan plot presents the -log10(P-values) for the enrichment of the gene-level signatures for smoking behavior (TWAS genes that surpassed correction for multiple comparisons and also demonstrated evidence of a shared causal variant in the gene locus.

© 2024 Rosoff DB et al. *JAMA Psychiatry.*

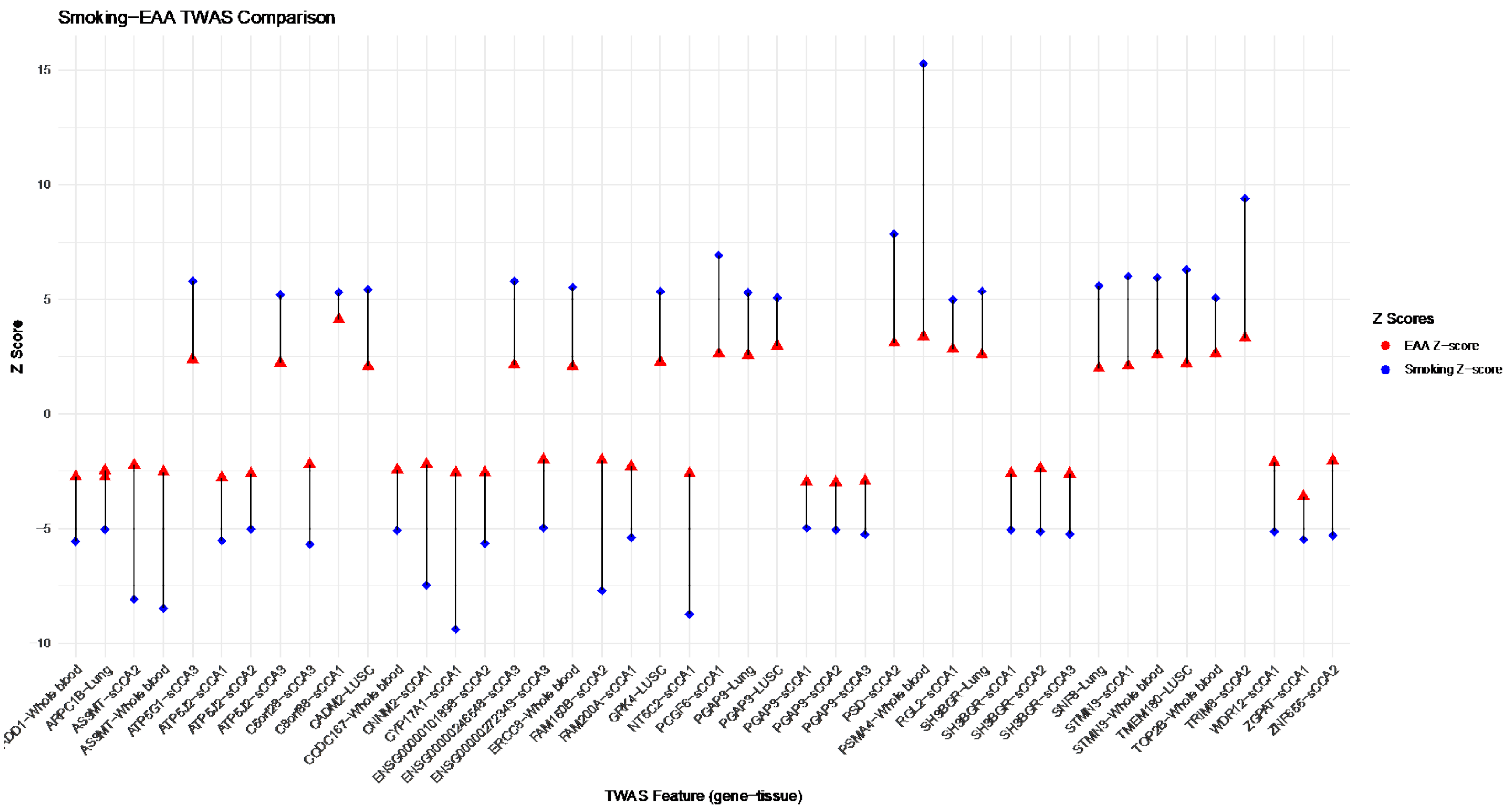
Smoking−EAA TWAS Comparison
Z Score
15
10
5
0
−5
−10
TWAS Feature (gene−tissue)
Z Scores
EAA Z−score
Smoking Z−score
DD1−Whole blood
ARPC1B−Lung
AS3MT−sCCA2
AS3MT−Whole blood
ATP5G1−sCCA3
ATP5J2−sCCA1
ATP5J2−sCCA2
ATP5J2−sCCA3
C5orf28−sCCA3
C8orf88−sCCA1
CADM2−LUSC
CCDC167−Whole blood
CNNM2−sCCA1
CYP17A1−sCCA1
ENSG00000101898−sCCA2
ENSG00000246548−sCCA3
ENSG00000272343−sCCA3
ERCC8−Whole blood
FAM150B−sCCA2
FAM200A−sCCA1
GRK4−LUSC
NT5C2−sCCA1
PCGF6−sCCA1
PGAP3−Lung
PGAP3−LUSC
PGAP3−sCCA1
PGAP3−sCCA2
PGAP3−sCCA3
PSD−sCCA2
PSMA4−Whole blood
RGL2−sCCA1
SH3BGR−Lung
SH3BGR−sCCA1
SH3BGR−sCCA2
SH3BGR−sCCA3
SNF8−Lung
STMN3−sCCA1
STMN3−Whole blood
TMEM180−LUSC
TOP2B−Whole blood
TRIM8−sCCA2
WDR12−sCCA1
ZGPAT−sCCA1
ZNF555−sCCA2

eFigure 13. Transcriptomic imputation comparison of the smoking-associated genes with epigenetic age acceleration. Plotted is the comparison of the high-confidence smoking-associated TWAS features (gene-tissue combinations) with the second-generation EAA clocks (*GrimAge* and *PhenoAge*) that were directionally consistent with the Mendelian randomization analyses (e.g., both increased smoking and EAA or vice versa) and also had TWAS P-values < 0.05 for the EAA models (**eMethods**). The y-axis is the TWAS Z-scores for smoking behavior and EAA for each gene-tissue feature analyzed.

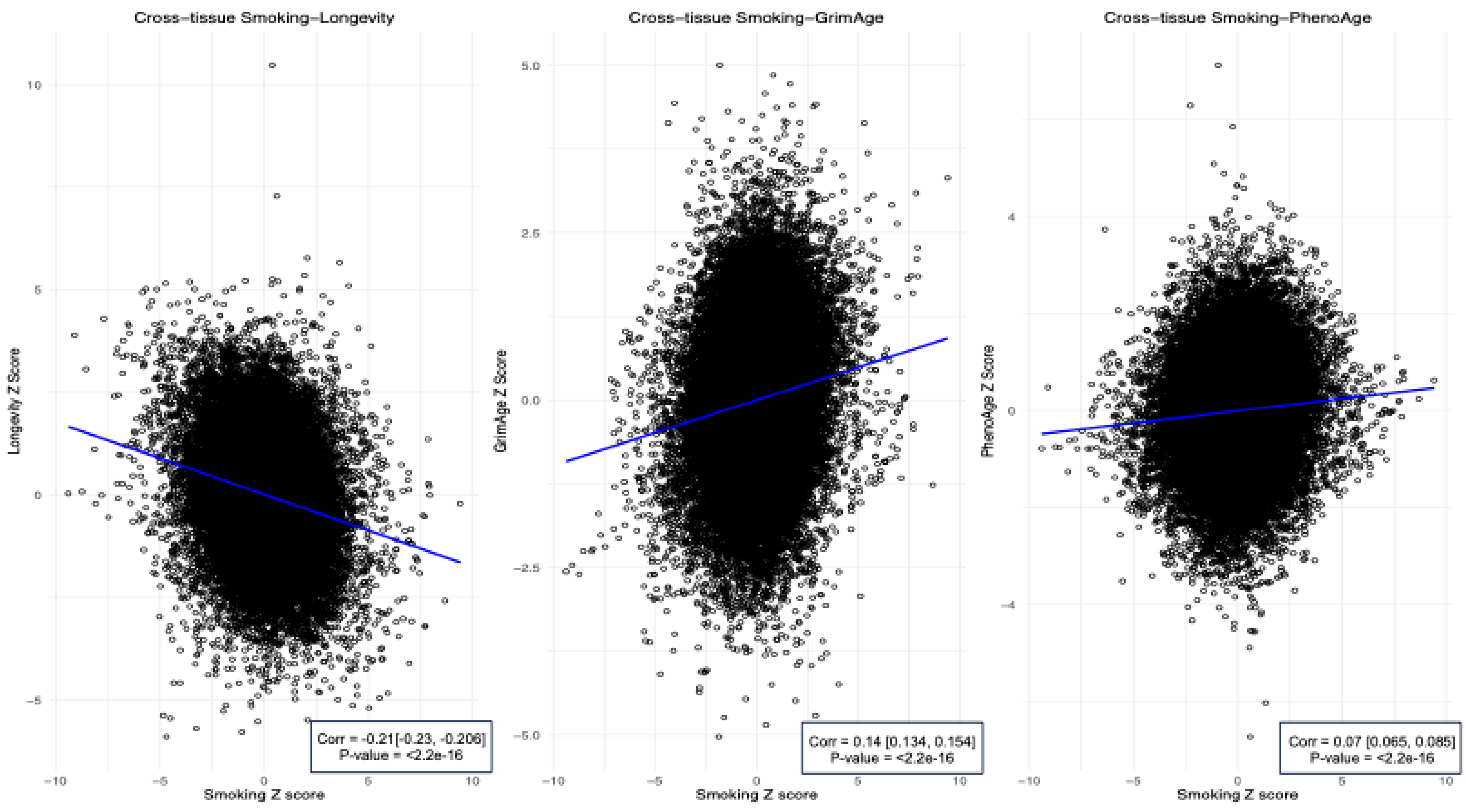

eFigure 14. Correlations of the transciptomic imputation results for smoking, multivariate longevity, GrimAge, and PhenoAge. Each panel presents correlations between the primary association statistics for the FUSION method (Z scores) for the full FUSION TWAS results using the FUSION gene expression weights derived from the Genotype-Tissue Expression project (GTEx) cross-tissue weights.

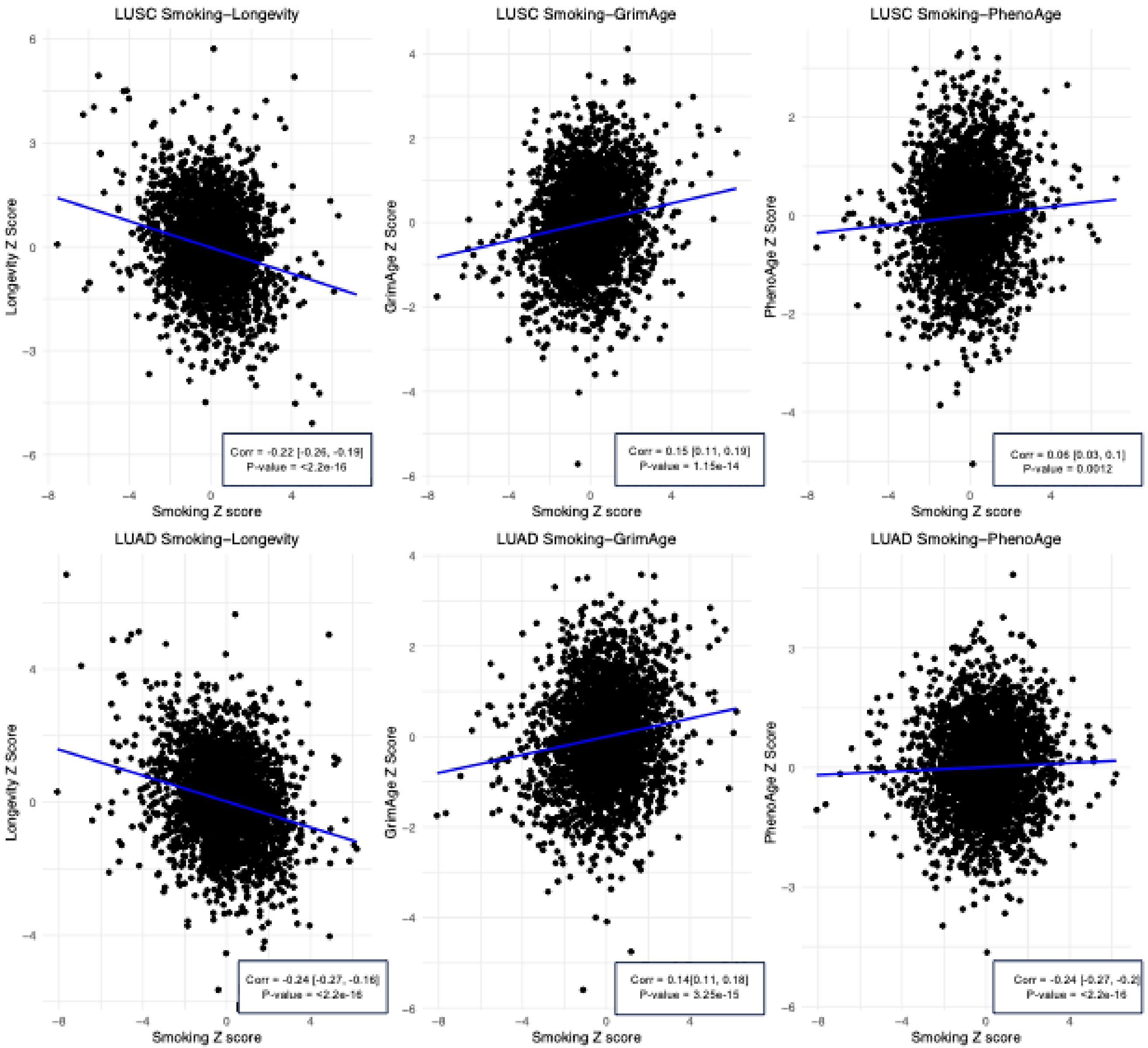
LUSC Smoking–Longevity
Longevity Z Score
Smoking Z score
Corr = -0.22 [-0.26, -0.19]
P-value = <2.2e-16
LUSC Smoking–GrimAge
GrimAge Z Score
Smoking Z score
Corr = 0.15 [0.11, 0.19]
P-value = 1.15e-14
LUSC Smoking–PhenoAge
PhenoAge Z Score
Smoking Z score
Corr = 0.06 [0.03, 0.1]
P-value = 0.0012
LUAD Smoking–Longevity
Longevity Z Score
Smoking Z score
Corr = -0.24 [-0.27, -0.16]
P-value = <2.2e-16
LUAD Smoking–GrimAge
GrimAge Z Score
Smoking Z score
Corr = 0.14[0.11, 0.18]
P-value = 3.25e-15
LUAD Smoking–PhenoAge
PhenoAge Z Score
Smoking Z score
Corr = -0.24 [-0.27, -0.2]
P-value = <2.2e-16

eFigure 15. Correlations of the transciptomic imputation results for smoking, multivariate longevity, GrimAge, and PhenoAge. Each panel presents correlations between the primary association statistics for the FUSION method (Z scores) for the full FUSION TWAS results using the FUSION gene expression weights derived from the The Cancer Genome Atlas (TGCA) squamus-cell carcinoma (LUSC) and adenocarcinoma (LUAD).

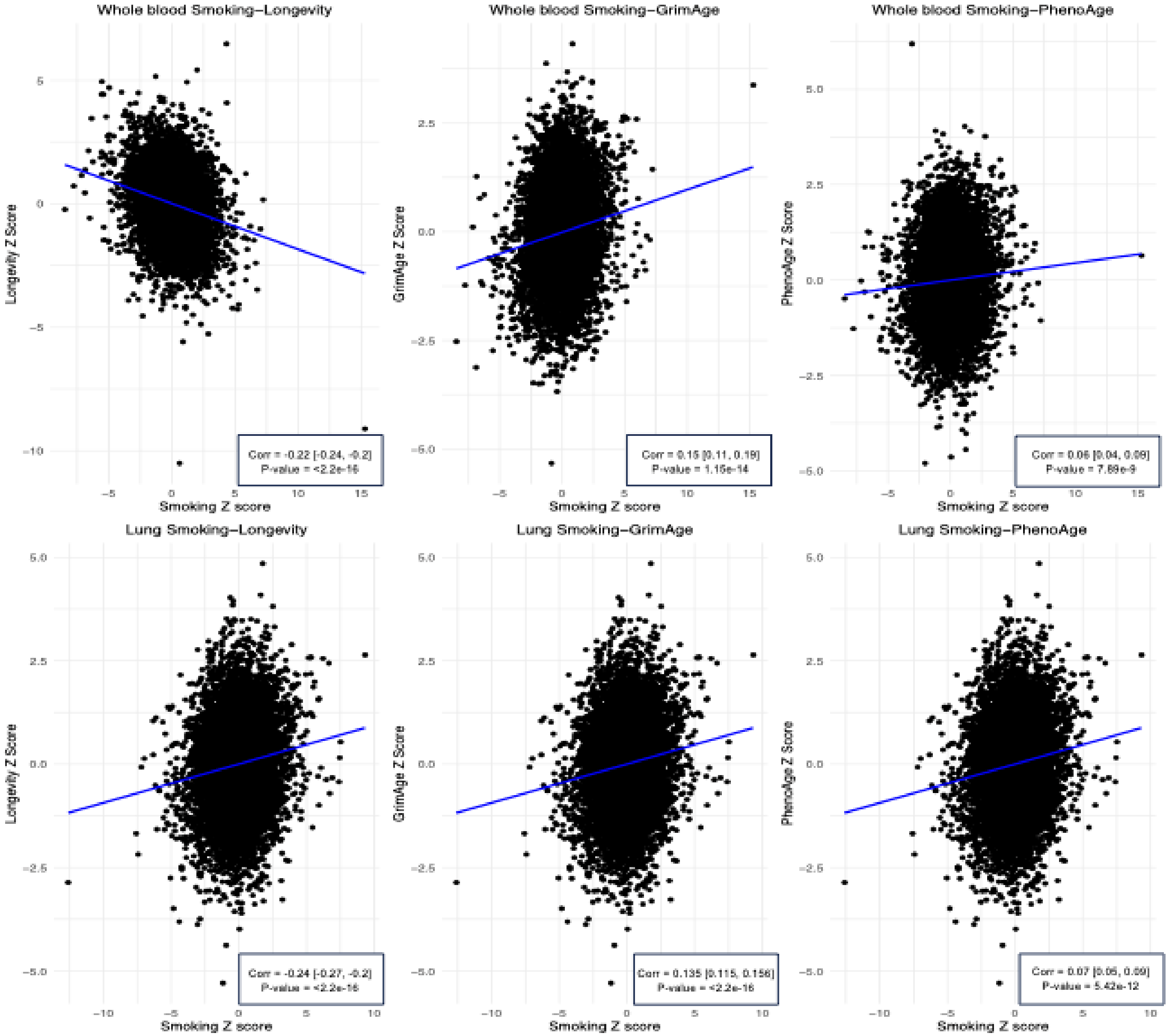

Whole blood Smoking–Longevity
Longevity Z Score
Smoking Z score
Corr = -0.22 [-0.24, -0.2]
P-value = <2.2e-16
Whole blood Smoking–GrimAge
GrimAge Z Score
Smoking Z score
Corr = 0.15 [0.11, 0.19]
P-value = 1.15e-14
Whole blood Smoking–PhenoAge
PhenoAge Z Score
Smoking Z score
Corr = 0.06 [0.04, 0.09]
P-value = 7.89e-9
Lung Smoking–Longevity
Longevity Z Score
Smoking Z score
Corr = -0.24 [-0.27, -0.2]
P-value = <2.2e-16
Lung Smoking–GrimAge
GrimAge Z Score
Smoking Z score
Corr = 0.135 [0.115, 0.156]
P-value = <2.2e-16
Lung Smoking–PhenoAge
PhenoAge Z Score
Smoking Z score
Corr = 0.07 [0.05, 0.09]
P-value = 5.42e-12

eFigure 16. Correlations of the transciptomic imputation results for smoking, multivariate longevity, GrimAge, and PhenoAge. Each panel presents correlations between the primary association statistics for the FUSION method (Z scores) for the full FUSION TWAS results using the FUSION gene expression weights derived from the Genotype-Tissue Expression project (GTEx) lung and whole blood tissues.

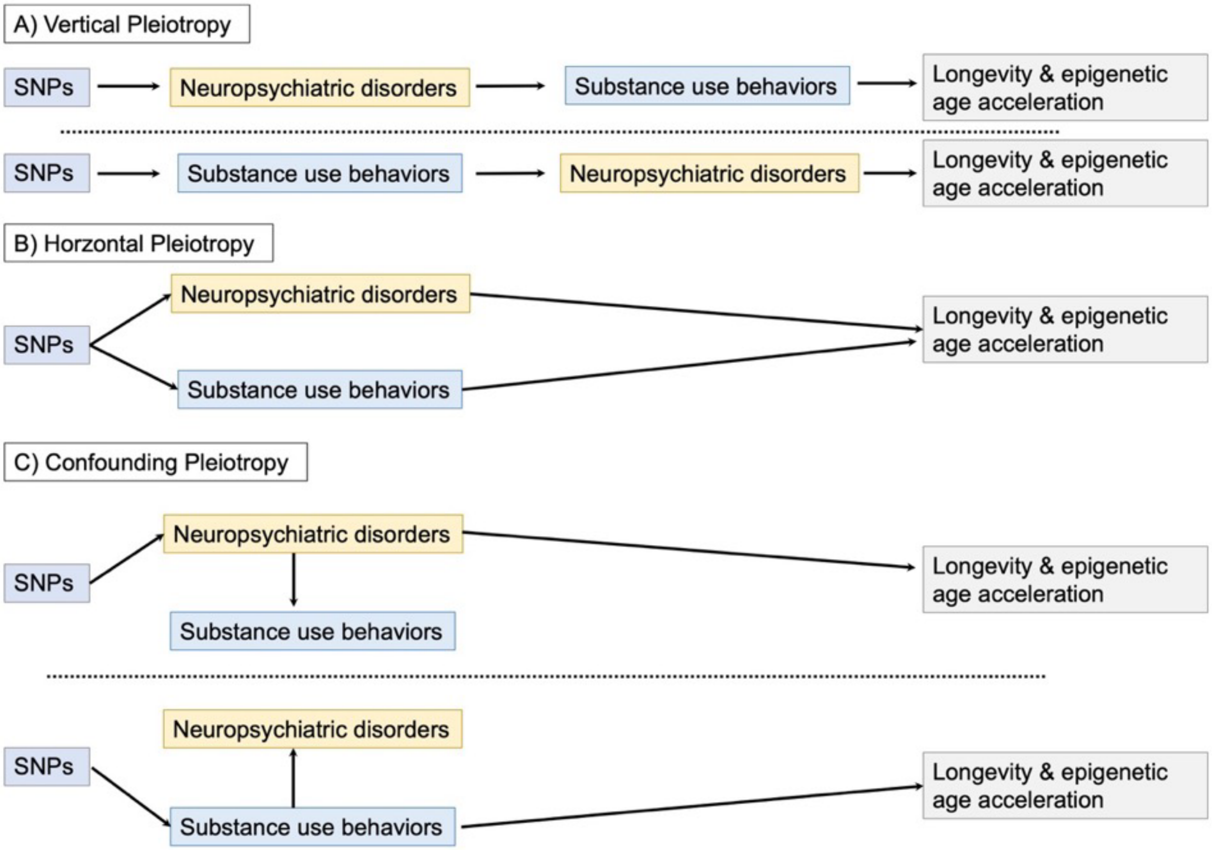

eFigure 17. Potential models explaining SNV associations for neuropsychiatric disorders, substance use behaviors, and longevity endpoints. (A) depicts potential vertical pleiotropy for two possible single variable MR study scenarios. Here SNVs (single nucleotide polymorphisms) act as instruments for genetic propensity for either neuropsychiatric disorders (i.e., Schizophrenia (SCZ), Major depressive disorder (MDD), etc.) or substance use behaviors (i.e., smoking and alcohol consumption); they would demonstrate vertical pleiotropy if all the genetic effects of their respective outcomes are mediated subsequently by their corresponding downstream impact (i.e., SCZ SNV instruments impacting smoking, which, in turn, impacts longevity). (B) depicts potential horizontal pleiotropy where SNVs have an impact on longevity via their associations with neuropsychiatric disorders and substance use behaviors lacking any downstream mediation. Confounding pleiotropy is depicted in (C): SNVs may be associated with longevity due to their impact on neuropsychiatric disorders and substance use behaviors; however, the genetic propensity for substance use behaviors has no corresponding direct impact on longevity.

SUPPLEMENTARY REFERENCES

1. Timmers PRHJ, Mounier N, Lall K, et al. Genomics of 1 million parent lifespans implicates novel pathways and common diseases and distinguishes survival chances. *eLife*. 2019/01/15 2019;8:e39856. doi:10.7554/eLife.39856
2. Sathyan S, Verghese J. Genetics of frailty: A longevity perspective. *Transl Res*. Jul 2020;221:83-96. doi:10.1016/j.trsl.2020.03.005
3. Timmers PRHJ, Wilson JF, Joshi PK, Deelen J. Multivariate genomic scan implicates novel loci and haem metabolism in human ageing. *Nature Communications*. 2020/07/16 2020;11(1):3570. doi:10.1038/s41467-020-17312-3
4. Palliyaguru DL, Moats JM, Di Germanio C, Bernier M, de Cabo R. Frailty index as a biomarker of lifespan and healthspan: Focus on pharmacological interventions. *Mech Ageing Dev*. Jun 2019;180:42-48. doi:10.1016/j.mad.2019.03.005
5. Zenin A, Tsepilov Y, Sharapov S, Getmantsev E, Menshikov LI, Fedichev PO, Aulchenko Y. Identification of 12 genetic loci associated with human healthspan. *Communications Biology*. 2019/01/30 2019;2(1):41. doi:10.1038/s42003-019-0290-0
6. Garmany A, Yamada S, Terzic A. Longevity leap: mind the healthspan gap. *npj Regenerative Medicine*. 2021/09/23 2021;6(1):57. doi:10.1038/s41536-021-00169-5
7. Beard JR, Officer A, de Carvalho IA, et al. The World report on ageing and health: a policy framework for healthy ageing. *Lancet*. May 21 2016;387(10033):2145-2154. doi:10.1016/s0140-6736(15)00516-4
8. McCartney DL, Min JL, Richmond RC, et al. Genome-wide association studies identify 137 loci for DNA methylation biomarkers of ageing. *bioRxiv*. 2020:2020.06.29.133702. doi:10.1101/2020.06.29.133702
9. Horvath S, Raj K. DNA methylation-based biomarkers and the epigenetic clock theory of ageing. *Nature Reviews Genetics*. 2018/06/01 2018;19(6):371-384. doi:10.1038/s41576-018-0004-3
10. Luo A, Jung J, Longley M, et al. Epigenetic aging is accelerated in alcohol use disorder and regulated by genetic variation in APOL2. *Neuropsychopharmacology*. Jan 2020;45(2):327-336. doi:10.1038/s41386-019-0500-y
11. McCartney DL, Min JL, Richmond RC, et al. Genome-wide association studies identify 137 genetic loci for DNA methylation biomarkers of aging. *Genome Biol*. Jun 29 2021;22(1):194. doi:10.1186/s13059-021-02398-9
12. Saunders GRB, Wang X, Chen F, et al. Genetic diversity fuels gene discovery for tobacco and alcohol use. *Nature*. 2022/12/01 2022;612(7941):720-724. doi:10.1038/s41586-022-05477-4
13. Kranzler HR, Zhou H, Kember RL, et al. Genome-wide association study of alcohol consumption and use disorder in 274,424 individuals from multiple populations. *Nature Communications*. 2019/04/02 2019;10(1):1499. doi:10.1038/s41467-019-09480-8
14. Sullivan PF, Agrawal A, Bulik CM, et al. Psychiatric Genomics: An Update and an Agenda. *Am J Psychiatry*. Jan 1 2018;175(1):15-27. doi:10.1176/appi.ajp.2017.17030283
15. Zhou H, Sealock JM, Sanchez-Roige S, et al. Genome-wide meta-analysis of problematic alcohol use in 435,563 individuals yields insights into biology and relationships with other traits. *Nature Neuroscience*. 2020/07/01 2020;23(7):809-818. doi:10.1038/s41593-020-0643-5
16. Deelen J, Evans DS, Arking DE, et al. A meta-analysis of genome-wide association studies identifies multiple longevity genes. *Nature communications*. 2019;10(1):3669-3669. doi:10.1038/s41467-019-11558-2

17. Hemani G, Tilling K, Smith GD. Orienting the causal relationship between imprecisely measured traits using GWAS summary data. *Plos Genetics*. Nov 2017;13(11)doi:ARTN e1007081
10.1371/journal.pgen.1007081
18. Burgess S, Thompson SG. Avoiding bias from weak instruments in Mendelian randomization studies. *Int J Epidemiol*. Jun 2011;40(3):755-64. doi:10.1093/ije/dyr036
19. Sanderson E, Glymour MM, Holmes MV, et al. Mendelian randomization. *Nature Reviews Methods Primers*. 2022/02/10 2022;2(1):6. doi:10.1038/s43586-021-00092-5
20. Sanderson E, Spiller W, Bowden J. Testing and Correcting for Weak and Pleiotropic Instruments in Two-Sample Multivariable Mendelian Randomisation. *bioRxiv (preprint)*. 2020:2020.04.02.021980. doi:10.1101/2020.04.02.021980
21. Rosoff DB, Davey Smith G, Mehta N, Clarke T-K, Lohoff FW. Evaluating the relationship between alcohol consumption, tobacco use, and cardiovascular disease: A multivariable Mendelian randomization study. *PLOS Medicine*. 2020;17(12):e1003410. doi:10.1371/journal.pmed.1003410
22. Yuan S, Larsson SC. A causal relationship between cigarette smoking and type 2 diabetes mellitus: A Mendelian randomization study. *Scientific Reports*. 2019/12/18 2019;9(1):19342. doi:10.1038/s41598-019-56014-9
23. Dare S, Mackay DF, Pell JP. Relationship between smoking and obesity: a cross-sectional study of 499,504 middle-aged adults in the UK general population. *PLoS One*. 2015;10(4):e0123579-e0123579. doi:10.1371/journal.pone.0123579
24. Berndt SI, Gustafsson S, Mägi R, et al. Genome-wide meta-analysis identifies 11 new loci for anthropometric traits and provides insights into genetic architecture. *Nat Genet*. May 2013;45(5):501-12. doi:10.1038/ng.2606
25. van der Harst P, Verweij N. Identification of 64 Novel Genetic Loci Provides an Expanded View on the Genetic Architecture of Coronary Artery Disease. *Circ Res*. Feb 2 2018;122(3):433-443. doi:10.1161/circresaha.117.312086
26. Xue A, Wu Y, Zhu Z, et al. Genome-wide association analyses identify 143 risk variants and putative regulatory mechanisms for type 2 diabetes. *Nature Communications*. 2018/07/27 2018;9(1):2941. doi:10.1038/s41467-018-04951-w
27. Kurki MI, Karjalainen J, Palta P, et al. FinnGen provides genetic insights from a well-phenotyped isolated population. *Nature*. 2023/01/01 2023;613(7944):508-518. doi:10.1038/s41586-022-05473-8
28. Schmidt AF, Finan C, Gordillo-Marañón M, et al. Genetic drug target validation using Mendelian randomisation. *Nature communications*. 2020;11(1):3255-3255. doi:10.1038/s41467-020-16969-0
29. Lassi G, Taylor AE, Timpson NJ, Kenny PJ, Mather RJ, Eisen T, Munafò MR. The CHRNA5-A3-B4 Gene Cluster and Smoking: From Discovery to Therapeutics. *Trends Neurosci*. Dec 2016;39(12):851-861. doi:10.1016/j.tins.2016.10.005
30. Jones SK, Wolf BJ, Froeliger B, Wallace K, Carpenter MJ, Alberg AJ. A systematic review of genetic variation within nicotinic acetylcholine receptor genes and cigarette smoking cessation. *Drug and Alcohol Dependence*. 2022/10/01/ 2022;239:109596. doi:https://doi.org/10.1016/j.drugalcdep.2022.109596
31. Hemani G, Zheng J, Elsworth B, et al. The MR-Base platform supports systematic causal inference across the human phenome. *Elife*. May 30 2018;7doi:10.7554/eLife.34408

32. Wootton RE, Jones HJ, Sallis HM. Mendelian randomisation for psychiatry: how does it work, and what can it tell us? *Molecular Psychiatry*. 2022/01/01 2022;27(1):53-57. doi:10.1038/s41380-021-01173-3
33. Wingo TS, Liu Y, Gerasimov ES, et al. Shared mechanisms across the major psychiatric and neurodegenerative diseases. *Nat Commun*. Jul 26 2022;13(1):4314. doi:10.1038/s41467-022-31873-5
34. Auton A, Brooks LD, Durbin RM, et al. A global reference for human genetic variation. *Nature*. Oct 1 2015;526(7571):68-74. doi:10.1038/nature15393
35. Yavorska OO, Burgess S. MendelianRandomization: an R package for performing Mendelian randomization analyses using summarized data. *International journal of epidemiology*. 2017;46(6):1734-1739. doi:10.1093/ije/dyx034
36. Davey Smith G, Hemani G. Mendelian randomization: genetic anchors for causal inference in epidemiological studies. *Human molecular genetics*. 2014;23(R1):R89-R98.
37. Smith GD. Use of genetic markers and gene-diet interactions for interrogating population-level causal influences of diet on health. *Genes & nutrition*. 2011;6(1):27-43.
38. Smith GD, Ebrahim S. 'Mendelian randomization': can genetic epidemiology contribute to understanding environmental determinants of disease? *Int J Epidemiol*. Feb 2003;32(1):1-22.
39. Davies NM, Holmes MV, Smith GD. Reading Mendelian randomisation studies: a guide, glossary, and checklist for clinicians. *Bmj*. 2018;362
40. Hemani G, Tilling K, Davey Smith G. Orienting the causal relationship between imprecisely measured traits using GWAS summary data. *PLOS Genetics*. 2017;13(11):e1007081. doi:10.1371/journal.pgen.1007081
41. Burgess S, Thompson SG, Collaboration CCG. Avoiding bias from weak instruments in Mendelian randomization studies. *International journal of epidemiology*. 2011;40(3):755-764.
42. Bowden J, Smith GD, Burgess S. Mendelian randomization with invalid instruments: effect estimation and bias detection through Egger regression. *International Journal of Epidemiology*. Apr 2015;44(2):512-525. doi:10.1093/ije/dyv080
43. Davey Smith G, Bowden J, Del Greco M F, Minelli C, Thompson JR, Sheehan NA. Assessing the suitability of summary data for two-sample Mendelian randomization analyses using MR-Egger regression: the role of the I2 statistic. *International Journal of Epidemiology*. 2016;45(6):1961-1974. doi:10.1093/ije/dyw220
44. Bowden J, Davey Smith G, Haycock PC, Burgess S. Consistent Estimation in Mendelian Randomization with Some Invalid Instruments Using a Weighted Median Estimator. *Genet Epidemiol*. May 2016;40(4):304-14. doi:10.1002/gepi.21965
45. Hartwig FP, Davey Smith G, Bowden J. Robust inference in summary data Mendelian randomization via the zero modal pleiotropy assumption. *Int J Epidemiol*. Dec 1 2017;46(6):1985-1998. doi:10.1093/ije/dyx102
46. Schmidt AF, Swerdlow DI, Holmes MV, et al. PCSK9 genetic variants and risk of type 2 diabetes: a mendelian randomisation study. *Lancet Diabetes Endocrinol*. Feb 2017;5(2):97-105. doi:10.1016/s2213-8587(16)30396-5
47. Bowden J, Del Greco MF, Minelli C, Smith GD, Sheehan N, Thompson J. A framework for the investigation of pleiotropy in two-sample summary data Mendelian randomization. *Statistics in Medicine*. May 20 2017;36(11):1783-1802. doi:10.1002/sim.7221
48. Bowden J, Del Greco MF, Minelli C, et al. Improving the accuracy of two-sample summary-data Mendelian randomization: moving beyond the NOME assumption. *Int J Epidemiol*. Jun 1 2019;48(3):728-742. doi:10.1093/ije/dyy258

49. Yavorska OO, Burgess S. MendelianRandomization: an R package for performing Mendelian randomization analyses using summarized data. *Int J Epidemiol*. Dec 1 2017;46(6):1734-1739. doi:10.1093/ije/dyx034
50. Rees JMB, Wood AM, Burgess S. Extending the MR-Egger method for multivariable Mendelian randomization to correct for both measured and unmeasured pleiotropy. *Stat Med*. Dec 20 2017;36(29):4705-4718. doi:10.1002/sim.7492
51. Rees JMB, Wood AM, Dudbridge F, Burgess S. Robust methods in Mendelian randomization via penalization of heterogeneous causal estimates. *PLoS One*. 2019;14(9):e0222362. doi:10.1371/journal.pone.0222362
52. Burgess S, Thompson SG. Multivariable Mendelian randomization: the use of pleiotropic genetic variants to estimate causal effects. *Am J Epidemiol*. Feb 15 2015;181(4):251-60. doi:10.1093/aje/kwu283
53. Sanderson E. Multivariable Mendelian Randomization and Mediation. *Cold Spring Harb Perspect Med*. Feb 1 2021;11(2)doi:10.1101/cshperspect.a038984
54. Sanderson E, Davey Smith G, Windmeijer F, Bowden J. An examination of multivariable Mendelian randomization in the single-sample and two-sample summary data settings. *International Journal of Epidemiology*. 2018;48(3):713-727. doi:10.1093/ije/dyy262
55. Hernán MA, Robins JM. Instruments for causal inference: an epidemiologist's dream? *Epidemiology*. Jul 2006;17(4):360-72. doi:10.1097/01.ede.0000222409.00878.37
56. Labrecque J, Swanson SA. Understanding the Assumptions Underlying Instrumental Variable Analyses: a Brief Review of Falsification Strategies and Related Tools. *Curr Epidemiol Rep*. 2018;5(3):214-220. doi:10.1007/s40471-018-0152-1
57. Burgess S, Bowden J, Fall T, Ingelsson E, Thompson SG. Sensitivity Analyses for Robust Causal Inference from Mendelian Randomization Analyses with Multiple Genetic Variants. *Epidemiology*. Jan 2017;28(1):30-42. doi:10.1097/ede.0000000000000559
58. Matthew SL, Louise ACM, George Davey S, Fernando H, Tom RG, Kate T. Examining the evidence for Mendelian randomization homogeneity assumption violation using instrument association with exposure variance. *medRxiv*. 2022:2022.09.12.22279854. doi:10.1101/2022.09.12.22279854
59. Kamat MA, Blackshaw JA, Young R, et al. PhenoScanner V2: an expanded tool for searching human genotype-phenotype associations. *Bioinformatics*. Nov 1 2019;35(22):4851-4853. doi:10.1093/bioinformatics/btz469
60. Burgess S, Davies NM, Thompson SG. Bias due to participant overlap in two-sample Mendelian randomization. *Genet Epidemiol*. Nov 2016;40(7):597-608. doi:10.1002/gepi.21998
61. Mounier N, Kutalik Z. Bias correction for inverse variance weighting Mendelian randomization. *Genet Epidemiol*. Jun 2023;47(4):314-331. doi:10.1002/gepi.22522
62. Minelli C, Del Greco MF, van der Plaat DA, Bowden J, Sheehan NA, Thompson J. The use of two-sample methods for Mendelian randomization analyses on single large datasets. *Int J Epidemiol*. Nov 10 2021;50(5):1651-1659. doi:10.1093/ije/dyab084
63. Mounier N, Kutalik Z. Bias correction for inverse variance weighting Mendelian randomization. *bioRxiv*. 2022:2021.03.26.437168. doi:10.1101/2021.03.26.437168
64. Riehm KE, Keyes KM, Susser ES. Social determinants of health and selection bias in genome-wide association studies. *World Psychiatry*. Feb 2023;22(1):160-161. doi:10.1002/wps.21047

65. Schoeler T, Speed D, Porcu E, Pirastu N, Pingault J-B, Kutalik Z. Participation bias in the UK Biobank distorts genetic associations and downstream analyses. *Nature Human Behaviour*. 2023/07/01 2023;7(7):1216-1227. doi:10.1038/s41562-023-01579-9
66. Fry A, Littlejohns TJ, Sudlow C, et al. Comparison of Sociodemographic and Health-Related Characteristics of UK Biobank Participants With Those of the General Population. *American Journal of Epidemiology*. Nov 1 2017;186(9):1026-1034. doi:10.1093/aje/kwx246
67. Gaziano JM, Concato J, Brophy M, et al. Million Veteran Program: A mega-biobank to study genetic influences on health and disease. *Journal of Clinical Epidemiology*. 2016/02/01/ 2016;70:214-223. doi:https://doi.org/10.1016/j.jclinepi.2015.09.016
68. Gemma LC, Ana G, Soares, et al. A framework for assessing selection and misclassification bias in mendelian randomisation studies: an illustrative example between body mass index and covid-19. *BMJ*. 2023;381:e072148. doi:10.1136/bmj-2022-072148
69. Arnold BF, Ercumen A, Benjamin-Chung J, Colford JM, Jr. Brief Report: Negative Controls to Detect Selection Bias and Measurement Bias in Epidemiologic Studies. *Epidemiology*. Sep 2016;27(5):637-41. doi:10.1097/ede.0000000000000504
70. Sanderson E, Richardson TG, Hemani G, Davey Smith G. The use of negative control outcomes in Mendelian randomization to detect potential population stratification. *International Journal of Epidemiology*. 2021;50(4):1350-1361. doi:10.1093/ije/dyaa288
71. Ben E, Matthew L, Tessa A, et al. The MRC IEU OpenGWAS data infrastructure. *bioRxiv*. 2020:2020.08.10.244293. doi:10.1101/2020.08.10.244293
72. Lawlor DA. Commentary: Two-sample Mendelian randomization: opportunities and challenges. *International Journal of Epidemiology*. 2016;45(3):908-915. doi:10.1093/ije/dyw127
73. Sudlow C, Gallacher J, Allen N, et al. UK biobank: an open access resource for identifying the causes of a wide range of complex diseases of middle and old age. *PLoS Med*. Mar 2015;12(3):e1001779. doi:10.1371/journal.pmed.1001779
74. Burgess S, Davey Smith G, Davies NM, et al. Guidelines for performing Mendelian randomization investigations: update for summer 2023. *Wellcome Open Res*. 2019;4:186. doi:10.12688/wellcomeopenres.15555.3
75. Didelez V, Sheehan N. Mendelian randomization as an instrumental variable approach to causal inference. *Stat Methods Med Res*. Aug 2007;16(4):309-30. doi:10.1177/0962280206077743
76. Hernán MA, Taubman SL. Does obesity shorten life? The importance of well-defined interventions to answer causal questions. *Int J Obes (Lond)*. Aug 2008;32 Suppl 3:S8-14. doi:10.1038/ijo.2008.82
77. Gandal MJ, Haney JR, Parikshak NN, et al. Shared molecular neuropathology across major psychiatric disorders parallels polygenic overlap. *Science*. 2018/02/09 2018;359(6376):693-697. doi:10.1126/science.aad6469
78. Bowden J, Vansteelandt S. Mendelian randomization analysis of case-control data using structural mean models. *Stat Med*. Mar 15 2011;30(6):678-94. doi:10.1002/sim.4138
79. Benca-Bachman CE, Bubier J, Syed RA, Romero Villela PN, Palmer RHC. Polygenic influences on the behavioral effects of alcohol withdrawal in a mixed-ancestry population from the collaborative study on the genetics of alcoholism (COGA). *Mol Cell Neurosci*. Jun 2023;125:103851. doi:10.1016/j.mcn.2023.103851
80. Deelen J, Evans DS, Arking DE, et al. A meta-analysis of genome-wide association studies identifies multiple longevity genes. *Nat Commun*. Aug 14 2019;10(1):3669. doi:10.1038/s41467-019-11558-2

81. Hartwig FP, Tilling K, Davey Smith G, Lawlor DA, Borges MC. Bias in two-sample Mendelian randomization when using heritable covariable-adjusted summary associations. *International Journal of Epidemiology*. 2021;50(5):1639-1650. doi:10.1093/ije/dyaa266
82. Uffelmann E, Huang QQ, Munung NS, et al. Genome-wide association studies. *Nature Reviews Methods Primers*. 2021/08/26 2021;1(1):59. doi:10.1038/s43586-021-00056-9
83. Labrecque JA, Swanson SA. Interpretation and Potential Biases of Mendelian Randomization Estimates With Time-Varying Exposures. *Am J Epidemiol*. Jan 1 2019;188(1):231-238. doi:10.1093/aje/kwy204
84. Shi J, Swanson SA, Kraft P, Rosner B, De Vivo I, Hernán MA. Mendelian Randomization With Repeated Measures of a Time-varying Exposure: An Application of Structural Mean Models. *Epidemiology*. Jan 1 2022;33(1):84-94. doi:10.1097/ede.0000000000001417
85. Labrecque Jeremy A, Swanson SA. Commentary: Mendelian randomization with multiple exposures: the importance of thinking about time. *International Journal of Epidemiology*. 2020;49(4):1158-1162. doi:10.1093/ije/dyz234
86. Tissink E, Werme J, de Lange SC, et al. The genetic architectures of functional and structural connectivity properties within cerebral resting-state networks. *eneuro*. 2023:ENEURO.0242-22.2023. doi:10.1523/ENEURO.0242-22.2023
87. Kretzschmar H. Brain banking: opportunities, challenges and meaning for the future. *Nat Rev Neurosci*. Jan 2009;10(1):70-8. doi:10.1038/nrn2535
88. Krassner MM, Kauffman J, Sowa A, et al. Postmortem changes in brain cell structure: a review. *Free Neuropathol*. Jan 2023;4doi:10.17879/freeneuropathology-2023-4790
89. Vornholt E, Luo D, Qiu W, et al. Postmortem brain tissue as an underutilized resource to study the molecular pathology of neuropsychiatric disorders across different ethnic populations. *Neurosci Biobehav Rev*. Jul 2019;102:195-207. doi:10.1016/j.neubiorev.2019.04.015
90. Wang L, Xia Y, Chen Y, et al. Brain Banks Spur New Frontiers in Neuropsychiatric Research and Strategies for Analysis and Validation. *Genomics Proteomics Bioinformatics*. Aug 2019;17(4):402-414. doi:10.1016/j.gpb.2019.02.002
91. Võsa U, Claringbould A, Westra HJ, et al. Large-scale cis- and trans-eQTL analyses identify thousands of genetic loci and polygenic scores that regulate blood gene expression. *Nat Genet*. Sep 2021;53(9):1300-1310. doi:10.1038/s41588-021-00913-z
92. Sun D, Gao W, Hu H, Zhou S. Why 90% of clinical drug development fails and how to improve it? *Acta Pharm Sin B*. Jul 2022;12(7):3049-3062. doi:10.1016/j.apsb.2022.02.002
93. Wootton RE, Richmond RC, Stuijfzand BG, et al. Evidence for causal effects of lifetime smoking on risk for depression and schizophrenia: a Mendelian randomisation study. *Psychol Med*. Oct 2020;50(14):2435-2443. doi:10.1017/s0033291719002678
94. Bulik-Sullivan BK, Loh P-R, Finucane HK, et al. LD Score regression distinguishes confounding from polygenicity in genome-wide association studies. *Nature Genetics*. 2015/03/01 2015;47(3):291-295. doi:10.1038/ng.3211
95. Gusev A, Ko A, Shi H, et al. Integrative approaches for large-scale transcriptome-wide association studies. *Nat Genet*. Mar 2016;48(3):245-52. doi:10.1038/ng.3506
96. Feng H, Mancuso N, Gusev A, Majumdar A, Major M, Pasaniuc B, Kraft P. Leveraging expression from multiple tissues using sparse canonical correlation analysis and aggregate tests improve the power of transcriptome-wide association studies. *bioRxiv*. 2020:2020.07.03.186247. doi:10.1101/2020.07.03.186247

97. Giambartolomei C, Vukcevic D, Schadt EE, Franke L, Hingorani AD, Wallace C, Plagnol V. Bayesian test for colocalisation between pairs of genetic association studies using summary statistics. *PLoS Genet*. May 2014;10(5):e1004383. doi:10.1371/journal.pgen.1004383
98. Kuleshov MV, Jones MR, Rouillard AD, et al. Enrichr: a comprehensive gene set enrichment analysis web server 2016 update. *Nucleic Acids Res*. Jul 8 2016;44(W1):W90-7. doi:10.1093/nar/gkw377
99. Ashburner M, Ball CA, Blake JA, et al. Gene Ontology: tool for the unification of biology. *Nature Genetics*. 2000/05/01 2000;25(1):25-29. doi:10.1038/75556
100. Gillespie M, Jassal B, Stephan R, et al. The reactome pathway knowledgebase 2022. *Nucleic Acids Research*. 2022;50(D1):D687-D692. doi:10.1093/nar/gkab1028
101. Kanehisa M, Furumichi M, Sato Y, Kawashima M, Ishiguro-Watanabe M. KEGG for taxonomy-based analysis of pathways and genomes. *Nucleic Acids Res*. Jan 6 2023;51(D1):D587-d592. doi:10.1093/nar/gkac963
102. Rafikova E, Nemirovich-Danchenko N, Ogmen A, et al. Open Genes—a new comprehensive database of human genes associated with aging and longevity. *Nucleic Acids Research*. 2023:gkad712. doi:10.1093/nar/gkad712
103. Dai Y, Hu R, Liu A, et al. WebCSEA: web-based cell-type-specific enrichment analysis of genes. *Nucleic Acids Research*. 2022;50(W1):W782-W790. doi:10.1093/nar/gkac392
104. Clarke DJB, Jeon M, Stein DJ, et al. Appyters: Turning Jupyter Notebooks into data-driven web apps. *Patterns*. 2021;2(3)doi:10.1016/j.patter.2021.100213
105. Lachmann A, Torre D, Keenan AB, et al. Massive mining of publicly available RNA-seq data from human and mouse. *Nature Communications*. 2018/04/10 2018;9(1):1366. doi:10.1038/s41467-018-03751-6
106. Lachmann A, Schilder BM, Wojciechowicz ML, Torre D, Kuleshov MV, Keenan AB, Ma'ayan A. Geneshot: search engine for ranking genes from arbitrary text queries. *Nucleic Acids Research*. 2019;47(W1):W571-W577. doi:10.1093/nar/gkz393
107. Zeng L, Yang J, Peng S, Zhu J, Zhang B, Suh Y, Tu Z. Transcriptome analysis reveals the difference between "healthy" and "common" aging and their connection with age-related diseases. *Aging Cell*. Mar 2020;19(3):e13121. doi:10.1111/acel.13121
108. Smeland OB, Bahrami S, Frei O, et al. Genome-wide analysis reveals extensive genetic overlap between schizophrenia, bipolar disorder, and intelligence. *Molecular Psychiatry*. 2020/04/01 2020;25(4):844-853. doi:10.1038/s41380-018-0332-x
109. Dashti S, Taherian-Esfahani Z, Keshtkar A, Ghafouri-Fard S. Associations between XRCC3 Thr241Met polymorphisms and breast cancer risk: systematic-review and meta-analysis of 55 case-control studies. *BMC Med Genet*. May 10 2019;20(1):79. doi:10.1186/s12881-019-0809-8
110. Matullo G, Palli D, Peluso M, et al. XRCC1, XRCC3, XPD gene polymorphisms, smoking and (32)P-DNA adducts in a sample of healthy subjects. *Carcinogenesis*. Sep 2001;22(9):1437-45. doi:10.1093/carcin/22.9.1437
111. Stern MC, Umbach DM, Lunn RM, Taylor JA. DNA repair gene XRCC3 codon 241 polymorphism, its interaction with smoking and XRCC1 polymorphisms, and bladder cancer risk. *Cancer Epidemiol Biomarkers Prev*. Sep 2002;11(9):939-43.
112. He F, Chang S-C, Wallar GM, Zhang Z-F, Cai L. Association of XRCC3 and XRCC4 gene polymorphisms, family history of cancer and tobacco smoking with non-small-cell lung cancer in a Chinese population: a case–control study. *Journal of Human Genetics*. 2013/10/01 2013;58(10):679-685. doi:10.1038/jhg.2013.78

113. Jiao L, Hassan MM, Bondy ML, Wolff RA, Evans DB, Abbruzzese JL, Li D. XRCC2 and XRCC3 Gene Polymorphismand Risk of Pancreatic Cancer. *Official journal of the American College of Gastroenterology | ACG*. 2008;103(2):360-367.
114. Schumacher B, Pothof J, Vijg J, Hoeijmakers JHJ. The central role of DNA damage in the ageing process. *Nature*. 2021/04/01 2021;592(7856):695-703. doi:10.1038/s41586-021-03307-7
115. Pal S, Tyler JK. Epigenetics and aging. *Science Advances*. 2(7):e1600584. doi:10.1126/sciadv.1600584
116. He X, Li T, Luo L, Zeng H, Chen Y, Cai S. PRMT6 mediates inflammation via activation of the NF-κB/p65 pathway on a cigarette smoke extract-induced murine emphysema model. *Tob Induc Dis*. 2020;18:8. doi:10.18332/tid/116413
117. Austin CA, Lee KC, Swan RL, et al. TOP2B: The First Thirty Years. *Int J Mol Sci*. Sep 14 2018;19(9)doi:10.3390/ijms19092765
118. Vijg J, Suh Y. Genome instability and aging. *Annu Rev Physiol*. 2013;75:645-68. doi:10.1146/annurev-physiol-030212-183715
119. Pommier Y, Nussenzweig A, Takeda S, Austin C. Human topoisomerases and their roles in genome stability and organization. *Nature Reviews Molecular Cell Biology*. 2022/06/01 2022;23(6):407-427. doi:10.1038/s41580-022-00452-3
120. Wang K, Liu H, Hu Q, et al. Epigenetic regulation of aging: implications for interventions of aging and diseases. *Signal Transduction and Targeted Therapy*. 2022/11/07 2022;7(1):374. doi:10.1038/s41392-022-01211-8
121. Migliaccio E, Giorgio M, Mele S, et al. The p66shc adaptor protein controls oxidative stress response and life span in mammals. *Nature*. Nov 18 1999;402(6759):309-13. doi:10.1038/46311
122. Mooijaart SP, van Heemst D, Schreuder J, et al. Variation in the SHC1 gene and longevity in humans1. *Experimental Gerontology*. 2004/02/01/ 2004;39(2):263-268. doi:https://doi.org/10.1016/j.exger.2003.10.001
123. Yang P, Li W, Li X. SHC1 Promotes Lung Cancer Metastasis by Interacting with EGFR. *J Oncol*. 2022;2022:3599832. doi:10.1155/2022/3599832
124. Liang Y, Lei Y, Du M, Liang M, Liu Z, Li X, Gao Y. The increased expression and aberrant methylation of SHC1 in non-small cell lung cancer: Integrative analysis of clinical and bioinformatics databases. *J Cell Mol Med*. Jul 2021;25(14):7039-7051. doi:10.1111/jcmm.16717
125. Besingi W, Johansson Å. Smoke-related DNA methylation changes in the etiology of human disease. *Human Molecular Genetics*. 2013;23(9):2290-2297. doi:10.1093/hmg/ddt621
126. Li T, Guo Y. ADP-Ribosylation Factor Family of Small GTP-Binding Proteins: Their Membrane Recruitment, Activation, Crosstalk and Functions. *Front Cell Dev Biol*. 2022;10:813353. doi:10.3389/fcell.2022.813353
127. Hartl FU. Cellular Homeostasis and Aging. *Annu Rev Biochem*. Jun 2 2016;85:1-4. doi:10.1146/annurev-biochem-011116-110806
128. Cao X, Wang Y, Xiong R, Muskhelishvili L, Davis K, Richter PA, Heflich RH. Cigarette whole smoke solutions disturb mucin homeostasis in a human in vitro airway tissue model. *Toxicology*. 2018/11/01/ 2018;409:119-128. doi:https://doi.org/10.1016/j.tox.2018.07.015
129. Gage SH, Bowden J, Smith GD, Munafo MR. Investigating causality in associations between education and smoking: a two-sample Mendelian randomization study. *Int J Epidemiol*. Aug 2018;47(4):1131-1140. doi:10.1093/ije/dyy131

130. Sanderson E, Davey Smith G, Windmeijer F, Bowden J. An examination of multivariable Mendelian randomization in the single-sample and two-sample summary data settings. *Int J Epidemiol*. Jun 1 2019;48(3):713-727. doi:10.1093/ije/dyy262
131. Roth GA, Mensah GA, Johnson CO, et al. Global Burden of Cardiovascular Diseases and Risk Factors, 1990-2019: Update From the GBD 2019 Study. *J Am Coll Cardiol*. Dec 22 2020;76(25):2982-3021. doi:10.1016/j.jacc.2020.11.010
132. Agency UHS. Chapter 2: major causes of death and how they have changed. Accessed June 10, 2022. https://www.gov.uk/government/publications/health-profile-for-england/chapter-2-major-causes-of-death-and-how-they-have-changed#references
133. Nie C, Li Y, Li R, et al. Distinct biological ages of organs and systems identified from a multi-omics study. *Cell Reports*. 2022/03/08/ 2022;38(10):110459. doi:https://doi.org/10.1016/j.celrep.2022.110459
134. Rojas-Saunero LP, Young JG, Didelez V, Ikram MA, Swanson SA. Considering Questions Before Methods in Dementia Research With Competing Events and Causal Goals. *American Journal of Epidemiology*. 2023;192(8):1415-1423. doi:10.1093/aje/kwad090
135. Osborn DPJ, Levy G, Nazareth I, Petersen I, Islam A, King MB. Relative Risk of Cardiovascular and Cancer Mortality in People With Severe Mental Illness From the United Kingdom's General Practice Research Database. *Archives of General Psychiatry*. 2007;64(2):242-249. doi:10.1001/archpsyc.64.2.242
136. Piatt EE, Munetz MR, Ritter C. An examination of premature mortality among decedents with serious mental illness and those in the general population. *Psychiatr Serv*. Jul 2010;61(7):663-8. doi:10.1176/ps.2010.61.7.663
137. Chesney E, Goodwin GM, Fazel S. Risks of all-cause and suicide mortality in mental disorders: a meta-review. *World Psychiatry*. Jun 2014;13(2):153-60. doi:10.1002/wps.20128
138. Moitra M, Santomauro D, Degenhardt L, Collins PY, Whiteford H, Vos T, Ferrari A. Estimating the risk of suicide associated with mental disorders: A systematic review and meta-regression analysis. *Journal of Psychiatric Research*. 2021/05/01/ 2021;137:242-249. doi:https://doi.org/10.1016/j.jpsychires.2021.02.053
139. Collaborators GBDCoD. Global, regional, and national age-sex-specific mortality for 282 causes of death in 195 countries and territories, 1980-2017: a systematic analysis for the Global Burden of Disease Study 2017. *Lancet*. Nov 10 2018;392(10159):1736-1788. doi:10.1016/S0140-6736(18)32203-7
140. Stephan Y, Sutin AR, Luchetti M, Caille P, Terracciano A. Cigarette smoking and personality change across adulthood: Findings from five longitudinal samples. *Journal of Research in Personality*. 2019/08/01/ 2019;81:187-194. doi:https://doi.org/10.1016/j.jrp.2019.06.006
141. Knudsen AK, Skogen JC. Monthly variations in self-report of time-specified and typical alcohol use: the Nord-Trøndelag Health Study (HUNT3). *BMC Public Health*. Feb 21 2015;15:172. doi:10.1186/s12889-015-1533-8
142. Fancourt D, Steptoe A. The longitudinal relationship between changes in wellbeing and inflammatory markers: Are associations independent of depression? *Brain Behav Immun*. Jan 2020;83:146-152. doi:10.1016/j.bbi.2019.10.004
143. Uher R, Zwicker A. Etiology in psychiatry: embracing the reality of poly-gene-environmental causation of mental illness. *World Psychiatry*. Jun 2017;16(2):121-129. doi:10.1002/wps.20436

144. Sakata R, McGale P, Grant EJ, Ozasa K, Peto R, Darby SC. Impact of smoking on mortality and life expectancy in Japanese smokers: a prospective cohort study. *BMJ : British Medical Journal*. 2012;345:e7093. doi:10.1136/bmj.e7093
145. Jha P, Ramasundarahettige C, Landsman V, et al. 21st-Century Hazards of Smoking and Benefits of Cessation in the United States. *New England Journal of Medicine*. 2013/01/24 2013;368(4):341-350. doi:10.1056/NEJMsa1211128
146. Gkatzionis A, Burgess S. Contextualizing selection bias in Mendelian randomization: how bad is it likely to be? *Int J Epidemiol*. Jun 1 2019;48(3):691-701. doi:10.1093/ije/dyy202
147. Howard DM, Adams MJ, Clarke TK, et al. Genome-wide meta-analysis of depression identifies 102 independent variants and highlights the importance of the prefrontal brain regions. *Nat Neurosci*. Mar 2019;22(3):343-352. doi:10.1038/s41593-018-0326-7
148. Lachance J, Tishkoff SA. SNV ascertainment bias in population genetic analyses: why it is important, and how to correct it. *Bioessays*. Sep 2013;35(9):780-6. doi:10.1002/bies.201300014
149. Wingo TS, Liu Y, Gerasimov ES, et al. Brain proteome-wide association study implicates novel proteins in depression pathogenesis. *Nat Neurosci*. Jun 2021;24(6):810-817. doi:10.1038/s41593-021-00832-6
150. Wingo TS, Gerasimov ES, Liu Y, et al. Integrating human brain proteomes with genome-wide association data implicates novel proteins in post-traumatic stress disorder. *Mol Psychiatry*. Jul 2022;27(7):3075-3084. doi:10.1038/s41380-022-01544-4
151. Robins C, Liu Y, Fan W, et al. Genetic control of the human brain proteome. *Am J Hum Genet*. Mar 4 2021;108(3):400-410. doi:10.1016/j.ajhg.2021.01.012
152. Aguet F, Anand S, Ardlie KG, et al. The GTEx Consortium atlas of genetic regulatory effects across human tissues. *Science*. 2020/09/11 2020;369(6509):1318-1330. doi:10.1126/science.aaz1776
153. Lohoff FW, Sorcher JL, Rosen AD, et al. Methylomic profiling and replication implicates deregulation of PCSK9 in alcohol use disorder. *Mol Psychiatry*. Sep 2018;23(9):1900-1910. doi:10.1038/mp.2017.168
154. Tylee DS, Kawaguchi DM, Glatt SJ. On the outside, looking in: a review and evaluation of the comparability of blood and brain "-omes". *Am J Med Genet B Neuropsychiatr Genet*. Oct 2013;162b(7):595-603. doi:10.1002/ajmg.b.32150
155. Xiang S, Jia T, Xie C, et al. Association between vmPFC gray matter volume and smoking initiation in adolescents. *Nature Communications*. 2023/08/15 2023;14(1):4684. doi:10.1038/s41467-023-40079-2
156. Cheng W, Rolls ET, Robbins TW, et al. Decreased brain connectivity in smoking contrasts with increased connectivity in drinking. *eLife*. 2019/01/08 2019;8:e40765. doi:10.7554/eLife.40765
157. Liu M, Jiang Y, Wedow R, et al. Association studies of up to 1.2 million individuals yield new insights into the genetic etiology of tobacco and alcohol use. *Nat Genet*. Feb 2019;51(2):237-244. doi:10.1038/s41588-018-0307-5
158. Michos ED, Reddy TK, Gulati M, et al. Improving the enrollment of women and racially/ethnically diverse populations in cardiovascular clinical trials: An ASPC practice statement. *Am J Prev Cardiol*. Dec 2021;8:100250. doi:10.1016/j.ajpc.2021.100250
159. Loree JM, Anand S, Dasari A, et al. Disparity of Race Reporting and Representation in Clinical Trials Leading to Cancer Drug Approvals From 2008 to 2018. *JAMA Oncology*. 2019;5(10):e191870-e191870. doi:10.1001/jamaoncol.2019.1870

160. Zheng J, Xu M, Walker V, et al. Evaluating the efficacy and mechanism of metformin targets on reducing Alzheimer's disease risk in the general population: a Mendelian randomization study. *medRxiv*. 2022:2022.04.09.22273625. doi:10.1101/2022.04.09.22273625
161. Fatumo S, Chikowore T, Choudhury A, Ayub M, Martin AR, Kuchenbaecker K. A roadmap to increase diversity in genomic studies. *Nature Medicine*. 2022/02/01 2022;28(2):243-250. doi:10.1038/s41591-021-01672-4
162. Bentley AR, Callier S, Rotimi CN. Diversity and inclusion in genomic research: why the uneven progress? *J Community Genet*. Oct 2017;8(4):255-266. doi:10.1007/s12687-017-0316-6
163. Sirugo G, Williams SM, Tishkoff SA. The Missing Diversity in Human Genetic Studies. *Cell*. Mar 21 2019;177(1):26-31. doi:10.1016/j.cell.2019.02.048
164. Borrell LN, Elhawary JR, Fuentes-Afflick E, et al. Race and Genetic Ancestry in Medicine — A Time for Reckoning with Racism. *New England Journal of Medicine*. 2021;384(5):474-480. doi:10.1056/NEJMms2029562
