# Supplementary material for: Major Psychiatric Disorders, Substance Use Behaviors, and Longevity
Source: JAMA Psychiatry. 2024 Jun 18;81(9):889–901. doi: 10.1001/jamapsychiatry.2024.1429 (PMC11195603; doi:10.1001/jamapsychiatry.2024.1429)
Supplement: Supplement 3. — Data sharing statement [file jamapsychiatry-e241429-s003.pdf]

Data Sharing Statement

Rosoff. Major Psychiatric Disorders, Substance Use Behaviors, and Longevity. *JAMA Psychiatry*. Published June 18, 2024. doi:10.1001/jamapsychiatry.2024.1429

Data
Data available: Yes
Data types: Other (please specify)
Additional Information: Data used in the study is available and we provide links to the sources. Full data to replicate the study findings are presented in the Supplement.
How to access data: We provide URLs to the source data and the data required to replicate the study findings are presented in the Supplement.
When available: With publication

Supporting Documents
Document types: Statistical/analytic code
How to access documents: We have provided URLs for each of the software packages used for the analyses in the study. We also make additional code - e.g., analysis pipelines - available upon request to the corresponding author.
When available: With publication

Additional Information
Who can access the data: Anyone requesting the data
Types of analyses: Any purpose.
Mechanisms of data availability: Investigator support.
